# Youth-associated protein TIMP2 regulates microglial state and function in healthy and aged mice

**DOI:** 10.1038/s41467-026-74906-z

**Published:** 2026-08-12

**Authors:** Brittany M. Hemmer, Sarah M. Philippi, Ana Catarina Ferreira, Samuele F. Petridis, Annie Phan, Joseph M. Castellano

**Affiliations:** 1 Nash Family Department of Neuroscience, Friedman Brain Institute, Icahn School of Medicine at Mount Sinai, New York, NY USA; 2 Ronald M. Loeb Center for Alzheimer’s Disease, Icahn School of Medicine at Mount Sinai, New York, NY USA; 3 Department of Neurology, Icahn School of Medicine at Mount Sinai, New York, NY USA; 4 Black Family Stem Cell Institute, Icahn School of Medicine at Mount Sinai, New York, NY USA; 5 Graduate School of Biomedical Sciences, Icahn School of Medicine at Mount Sinai, New York, NY USA

**Keywords:** Neural ageing, Glial biology, Neuroimmunology, Cellular neuroscience, Neuro-vascular interactions

## Abstract

There is little understanding of how aging serves as the strongest risk factor for several neurodegenerative diseases. Microglia undergo age-related maladaptive changes, including increased inflammation, impaired debris clearance, and cellular senescence, yet specific mediators that regulate these processes remain unclear. The aged brain is rejuvenated by youth-associated plasma factors, including tissue inhibitor of metalloproteinases 2 (TIMP2), which we have shown acts on the extracellular matrix (ECM) to regulate synaptic plasticity. Given emerging roles for microglia in these processes, we examined the impact of TIMP2 on microglial function. We show that TIMP2 deletion in mice exacerbates microglial phenotypes associated with aging, including transcriptomic changes in cell activation, changes in lysosomal-associated markers and phagocytosis, and elevated levels of stress and inflammatory proteins in the brain extracellular space measured by in vivo microdialysis. Deleting specific cellular pools of TIMP2 in vivo increases microglial CD68 and alters myelin phagocytosis. Treating aged mice with TIMP2 reverses several phenotypes observed in our deletion models, resulting in decreased microglial activation, reduced proportions of proinflammatory microglia, and enhanced phagocytosis of physiological substrates. Our results identify TIMP2 as a modulator of age-associated microglia dysfunction. Harnessing its activity may mitigate detrimental effects of age-associated insults on microglia function.

## Abstract (web summary)

Aging disrupts microglial function, but factors that rejuvenate these cells are poorly characterized. Here, the authors show the youth-associated protein TIMP2 revitalizes microglia by altering inflammatory states and restoring phagocytosis.

## Introduction

Aging is accompanied by progressive functional decline across multiple organ systems, but its impact on the brain is especially devastating, given the dysfunction wrought across key cognitive domains and the increased risk it poses for several neurological disorders. These changes reflect cumulative cellular, molecular, and structural perturbations, including loss of synaptic and myelin integrity, and neuroinflammation in vulnerable brain regions like the hippocampus. Understanding mechanisms that regulate aging in the hippocampus is thus essential for identifying new strategies to preserve or restore function across the lifespan and to limit risk for neurological disease. Interestingly, aging and neurodegeneration pose similar challenges to the brain’s immune environment, including aberrant inflammation and elevated exposure to lipid-rich debris from dying cells^1^. The role of microglia, the brain’s innate immune cells, in responding to aging-associated damage has emerged as a promising avenue for investigation, galvanized by the identification of many myeloid-enriched genes associated with Alzheimer’s risk in a series of GWAS over the past decade^2^. While specific states adopted by microglia may initially limit pathology to surrounding brain tissue, chronic challenges and exposure to debris may ultimately lead to maladaptive responses^3–6^ that can manifest as phagocytic ineptitude^7–10^, production of inflammatory cytokines^11,12^, and increased stress responses^6,13^. Thus, identifying processes that rejuvenate responses of these cells to aging-associated challenges may provide a path for novel therapies limiting the impact of aging on neurological disorders.

Modulating the systemic environment has emerged as one promising strategy to counteract age-related decline in cellular function. Exposure to factors present within young blood rejuvenates the aged brain^14,15^, leading to increased synaptic plasticity^16–19^ and dendritic spine integrity^17^, enhanced vascular remodeling^20^, and improved learning and memory performance in aged mice^16–19^. While much of this work has focused on the impact of blood-borne factors directly on neurons or adult neuroblasts in the hippocampus, recent work has suggested that microglia can take up proteins originating in plasma^21,22^, suggesting that they may be responsive to blood-borne factors. Moreover, in response to treatment with plasma or a platelet factor acting on immune cells in the periphery, microglia appear to exhibit altered phagocytosis^22^ and activation state^18^, respectively. Despite these findings, the field lacks a detailed characterization of how specific youth-associated factors affect microglial function and how such factors can be harnessed to revitalize the aged brain.

We previously identified tissue inhibitor of metalloproteinases 2 (TIMP2) as a youth-associated blood-borne factor capable of revitalizing hippocampal function in aged mice^16^. Levels of TIMP2 are elevated in very young human and mouse plasma and rapidly decline into adulthood and old age^16^. Interestingly, a recent study finds that those expressing *TIMP2* variants associated with higher plasma protein levels exhibit higher cognitive performance in a cohort of aged individuals at risk for developing AD^23^. Aged mice treated systemically with TIMP2 exhibit improvements resembling those seen in mice treated with young plasma, including increased hippocampal plasticity and improved memory, likely as a result of direct TIMP2 action on CNS cells, given its entry from blood into brain^16^. While TIMP2 has many canonical and non-canonical targets^24,25^, these studies suggest that TIMP2 has myriad roles within the hippocampal microenvironment, yet its role in modulating innate immune function is understudied. We recently reported that TIMP2 plays a critical role in the young hippocampus in regulating memory, synaptic integrity, and adult neurogenesis through modulation of ECM homeostasis^26^, processes that have been found to be regulated by microglia^27,28^. Given the modulation by TIMP2 in processes linked to microglia function and the rejuvenating capacity of TIMP2 in aging contexts, we sought to determine how TIMP2 affects basic microglial function and whether systemic supplementation can restore protective microglial activities that are lost in the setting of age-related insults.

Here, we show that loss of TIMP2 expression from a variety of sources (i.e., removal from global, microglial, and neuronal sources) affects microglia by altering their inflammatory state and shifting phagocytosis, processes associated with aging. Restoring the extrinsic pool of TIMP2 in aged mice through systemic TIMP2 treatment reversed these age-associated phenotypes by reducing lysosomal-associated markers in microglia, specific inflammatory microglial subpopulations, and by increasing phagocytosis of physiological substrates by microglia in the hippocampus. Our results demonstrate that the youth-associated protein TIMP2 plays a role in modulating microglial function by shifting their responses to accommodate detrimental aging pathologies in a protective fashion. This work firmly positions TIMP2 as a relevant therapeutic target to limit aging pathology, highlighting the need for further mechanistic investigation into how systemic factors modulate innate immune function in the brain.

## Results

### Early deletion of TIMP2 alters microglial state

**Fig. 1:**
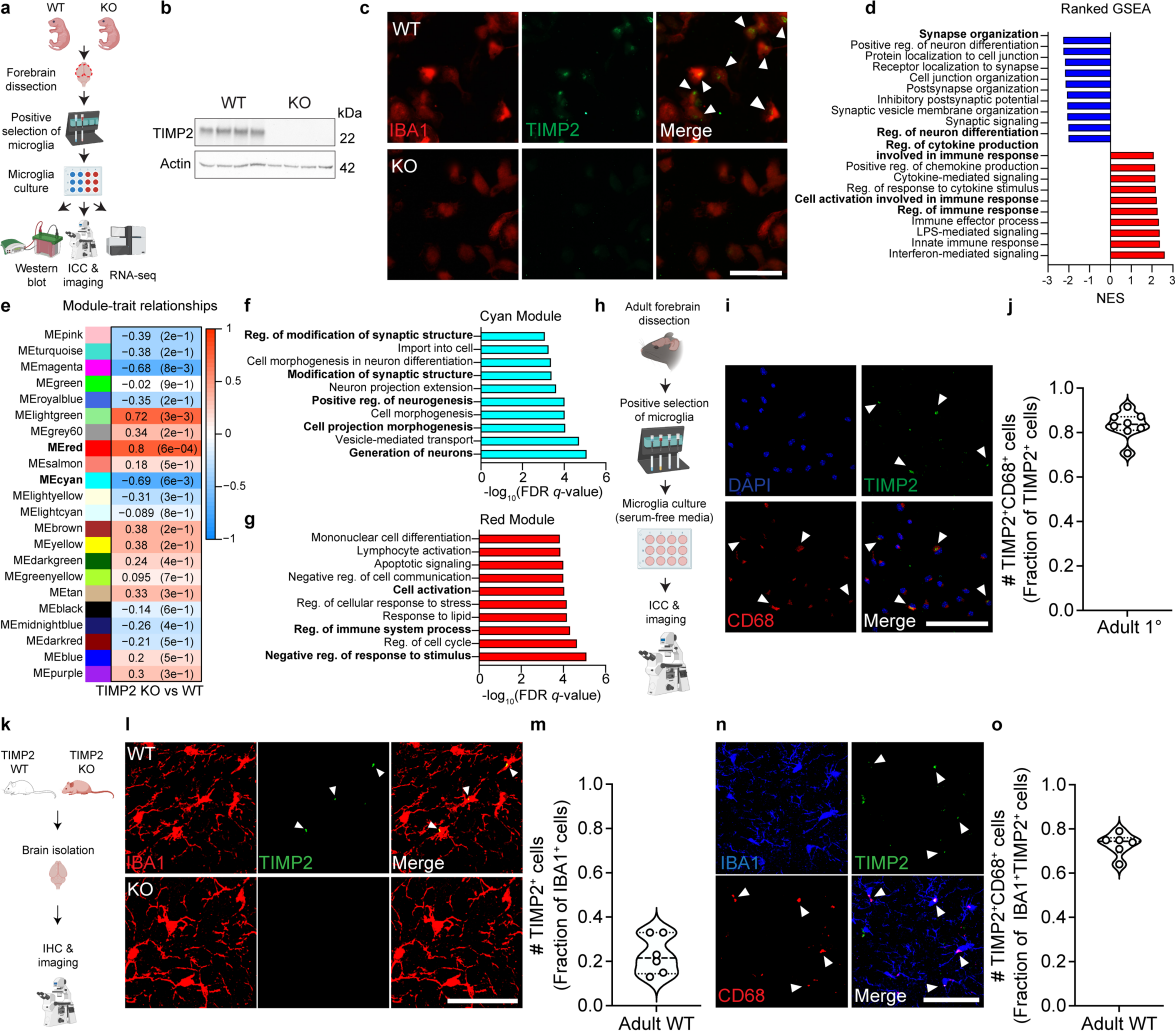
TIMP2 is expressed in microglia and its deletion leads to phenotypes associated with increased activation. **a** Schematic diagram of assays performed on TIMP2 KO and WT primary microglia. **b** TIMP2 immunoblotting from primary microglia lysates isolated from early postnatal TIMP2 WT and KO littermates (*N* = 4 samples per genotype, comprised of 5–6 pups (P5–P8) pooled per sample). **c** Representative images from cultures of TIMP2 KO and WT primary microglia (*N* = 3 pups (P6–P7) pooled per genotype; repeated in 2 additional experiments) stained with anti-TIMP2 and anti-IBA1 antibodies by ICC (scale bar = 100 μm). **d** Ranked GSEA using two-tailed Wald statistic-ranked DEGs from bulk RNAseq of TIMP2 KO and WT primary microglia (*N* = 7 samples per genotype, with each sample representing a pool of 5 pups (P6-P7); sex-matched; mean ± SEM), depicting over-representation analysis of GO Biological Processes (GOBP) pathways (*P*_adj_ < 0.05; Benjamini-Hochberg adjustment) and normalized enrichment score (NES). **e** Module-trait relationships plot based on WGCNA from TIMP2 KO vs. WT bulk RNAseq dataset, with colored module assignments and corresponding membership values and module significance (*P* < 0.05, two-tailed Student’s asymptotic *t*-test) in parentheses, with **f** significant GOBP terms (FDR *q*-value < 0.05) from over-representation analysis on significant “Cyan” and **g** “Red” modules on extracted genes. **h** Schematic diagram of adult microglia isolation, culturing, and imaging. **i** Representative confocal images from WT primary microglia cultures stained by ICC with DAPI, anti-TIMP2 antibody, and anti-CD68 antibody (*N* = 8 mice, 5–6-month-old mice; sex-matched; 2 independent experiments combined; scale bar = 100 μm), with **j** quantification of fraction of TIMP2^+^ cells that are TIMP2^+^CD68^+^; violin plot of median with quartiles. **k** A schematic diagram of the confocal imaging workflow from adult mouse brain sections. **l** Representative confocal images of dentate gyrus (DG) of 6–7-month-old TIMP2 KO and WT mice stained with anti-IBA1 and anti-TIMP2 antibodies (scale bar = 50 µm; arrowheads indicate TIMP2^+^ cells) with **m** quantification of fraction of IBA1^+^ cells that are TIMP2^+^; violin plot of median with quartiles (*N* = 6 mice). **n** Representative confocal images of DG of 6–7-month-old WT mice stained with anti-IBA1, -TIMP2, and -CD68 antibodies (scale bar = 50 µm; arrowheads indicate TIMP2^+^CD68^+^ cells), with **o** corresponding fraction of IBA1^+^TIMP2^+^ cells that are TIMP2^+^CD68^+^; violin plot of median with quartiles (*N* = 6 mice). Source data provided as Source Data file. Created in BioRender: Philippi, S. (2026) https://BioRender.com/6kzosyz.

TIMP2 is expressed in cells of the periphery, contributing to high levels in the plasma^16^, and also in hippocampal neurons^26^, with expression in both compartments declining with age^16^. Interestingly, *Timp2* has been shown to be upregulated in subpopulations of microglia in response to aging and AD pathology as part of a panel of genes termed the disease-associated microglia (DAM) profile^29^. To explore its expression within microglia, we noted its enriched transcript abundance within microglia in published transcriptomic atlases, including in *Tabula Muris Senis*^30^, and in the Allen Brain Cell scRNAseq and MERFISH Atlas^31^, in which *Timp2* is expressed at high levels in the brain, particularly in hippocampal microglia (Supplementary Fig. 1a–c), though it is also expressed in cortical and striatal microglia (Supplementary Fig. 1d–g). To begin to characterize whether microglia express TIMP2 at the protein level and how its loss influences function at early stages, we isolated primary microglia from early postnatal (P5-P8) forebrain from wild-type (WT) and TIMP2 knockout (KO) mice (Fig. 1a) at an age when global TIMP2 levels are high^16^. We find high levels of TIMP2 protein by both immunoblotting of primary microglia lysates and immunocytochemistry (ICC), whereas TIMP2 was not detected in TIMP2 KO microglia (Fig. 1b, c). We next sought to understand what pathways may be disrupted by its loss in microglia by performing bulk RNA-sequencing on WT and KO early postnatal (P6-P7) primary microglia (Fig. 1a). We detected a number of differentially expressed genes (DEGs) in KO microglia relative to WT (Supplementary Fig. 1h), with significantly reduced *Timp2* transcript in KO microglia, as expected (Supplementary Fig. 1i). To gain insight into what pathways are perturbed in microglia by TIMP2 deletion, we first performed a ranked gene set enrichment analysis (GSEA) on the dataset^32^, revealing upregulated processes in KO microglia that include “cell activation involved in immune response” and “regulation of cytokine production” (Fig. 1d), and downregulated processes related to “synapse organization” and “neuron differentiation.” Over-representation analyses^33^ for gene ontology terms related to biological processes performed on DEGs (*P*_adj_ < 0.05) in the dataset largely showed similar pathways (Supplementary Fig. 1j), reflecting perturbations in associated genes, including *H2-Aa, Itgal, Arsg, Csf2ra*, that are linked to myeloid signaling, inflammation, and lysosomal function. Moreover, we also identified enrichment in a gene set for microglial senescence-associated genes (FDR < 0.05; Supplementary Fig. 1k). We next applied a weighted gene correlation network analysis (WGCNA)-based approach to examine correlated sets of altered genes from which pathways could be identified, revealing several significant modules associated with TIMP2 deletion (Fig. 1e). Pathways from genes extracted from the downregulated Cyan and Magenta modules were related to neurogenesis, synaptic structure, organization, and morphogenesis (Fig. 1f, Supplementary Fig. 1l). Pathways from the upregulated Red module were related to “cell activation” and “regulation of the immune system process”, among others (Fig. 1g). Overall, the transcriptomic profile of KO microglia appears to reflect a potentially more activated and inflammatory state relative to WT microglia that may affect their interactions with neurons and ability to promote plasticity.

Given TIMP2’s potential relationship with activation state, we stained cultures of primary microglia isolated from adult (5–6-month-old) mice and found that the majority of TIMP2-expressing cells co-express CD68, a lysosomal-associated protein found enriched in myeloid cells (Fig. 1h–j), pointing to a potential role for TIMP2 in regulating lysosomal function and its links to activation state. While not all microglia were found to express TIMP2, a sizeable fraction of CD68-expressing microglia co-expressed TIMP2 (Supplementary Fig. 1m–o). To examine whether similar patterns of TIMP2 expression are observed in the adult mouse brain, we immunostained brain sections from adult (6–7-month-old) WT mice with antibodies for TIMP2 and microglia marker IBA1 for confocal imaging, which revealed that ~21% of microglia in the hippocampus express TIMP2 protein, expression that was absent in KO mice (Fig. 1k–m). TIMP2 protein was also detected in microglia imaged in the striatum, and to a lesser extent in the cortex (Supplementary Fig. 1p–r), broadly reflecting its gene expression pattern (Supplementary Fig. 1b–g). Similar to the pattern in primary microglia culture, a majority of TIMP2^+^ microglia in WT hippocampus co-expressed CD68 (Fig. 1n, o).

### TIMP2 deletion alters microglial phenotypes and increases extracellular inflammatory proteins

**Fig. 2:**
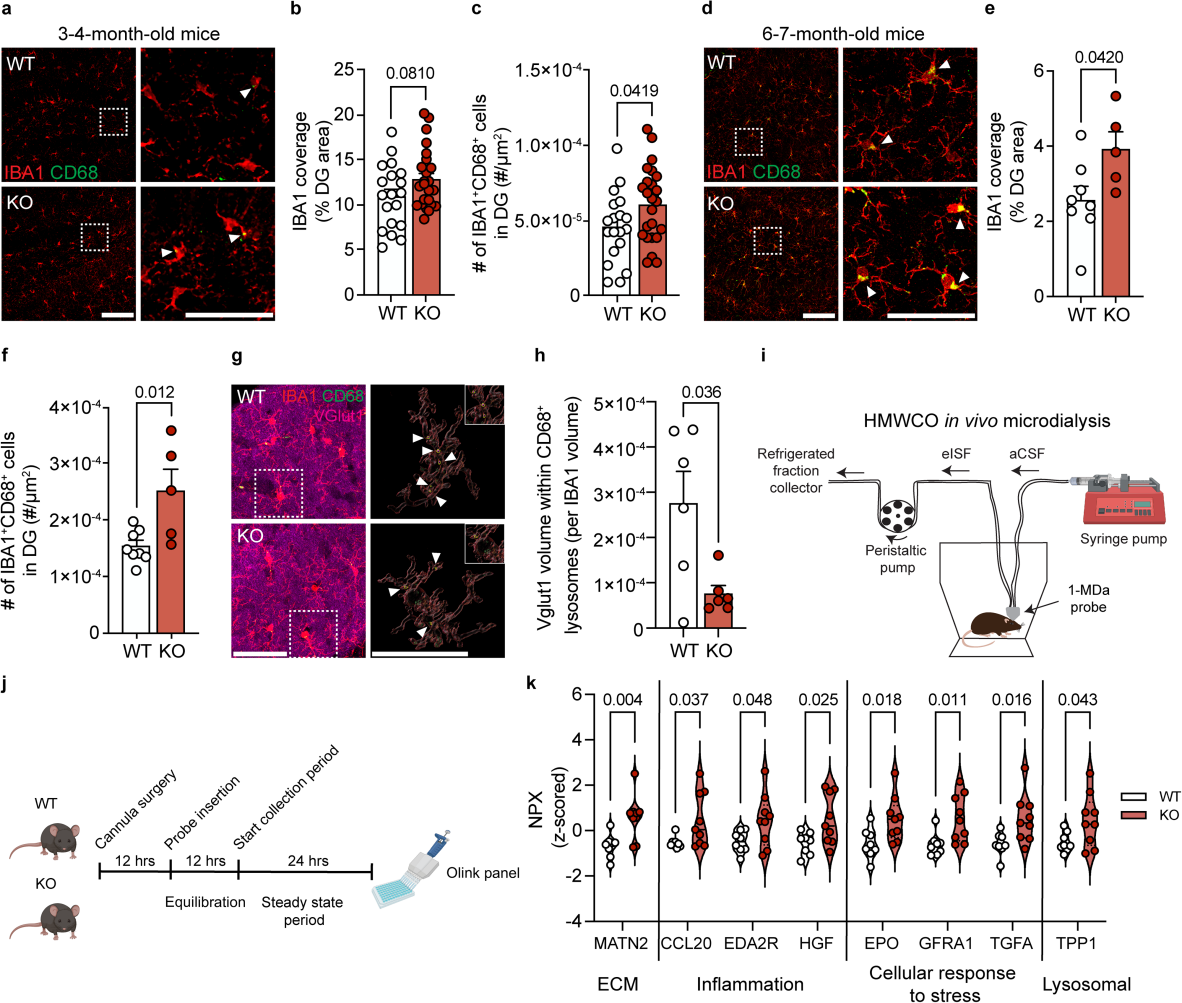
TIMP2 regulates microglial state and brain extracellular composition in vivo. **a** Representative confocal images from DG of 3–4-month-old TIMP2 KO and WT mice stained with anti-IBA1 and anti-CD68 antibodies (left; scale bar = 100 μm) and magnified inset (right; scale bar = 50 μm; arrowheads indicate IBA1^+^CD68^+^ cells) with corresponding **b** quantification of thresholded DG area covered by IBA1^+^ staining and **c** the number of IBA1^+^CD68^+^ cells normalized to DG area; mean ± SEM; *N* = 19 WT (9 female, 10 male), 24 KO (13 female, 11 male). **d** Representative confocal images of DG of 6–7-month-old TIMP2 KO and WT mice stained with anti-IBA1 and anti-CD68 antibodies (left; scale bar = 100 μm) and magnified inset (right; scale bar = 50 μm); arrowheads indicate IBA1^+^CD68^+^ cells) with corresponding **e** quantification of thresholded DG area covered by IBA1^+^ staining and **f** the number of IBA1^+^CD68^+^ cells normalized to DG area; *N* = 8 WT, 5 KO female mice; mean ± SEM. **g** Representative confocal images (left) of DG of 6–7-month-old TIMP2 KO and WT mice stained with anti-IBA1, anti-CD68, and anti-Vglut1 antibodies (scale bars = 50 µm), with Imaris-based reconstruction (right) of insetted microglial volume (red), containing engulfed Vglut1^+^ puncta (purple with yellow outlines indicated by white arrowheads) within microglial lysosomes (green) (scale bar = 50 µm). **h** Quantification of the total volume of Vglut^+^ signal within CD68^+^ volume, normalized to microglia volume. Values represent *N* = 6 mice per group; mean ± SEM. **i** Schematic diagram of the high molecular-weight cut-off (HMWCO,1-MDa) in vivo microdialysis method to dialyze hippocampal ISF proteins and corresponding **j** timeline of microdialysis experiments to evaluate steady state levels of hippocampal ISF proteins in 2–3-month-old WT (*N* = 8) and TIMP2 KO (*N* = 9) female mice with corresponding **k** brain ISF protein measurements, represented as z-scored NPX values for significant proteins (median with quartiles). Two-tailed Student’s *t*-test used in panels (**b**, **c**, **e**, **f**), with Welch’s correction in (**h**, **k**); Source data provided as Source Data file. Created in BioRender: Philippi, S. (2026) https://BioRender.com/38xw96n.

To evaluate the extent to which the putative immune activation-related pathways identified in our profiling of KO and WT microglia may reflect microglial phenotypes in vivo, we next sought to characterize microglial reactivity in KO and WT dentate gyrus of the adult hippocampus, a relevant region based on previous work demonstrating its role in regulating plasticity processes^16,26^. We first evaluated co-staining for IBA1 and the phagolysosomal-associated marker CD68 in this region in TIMP2 KO and WT mice. As early as 3–4 months of age, KO mice exhibited a modest increase that did not reach statistical significance in the percentage of dentate gyrus (DG) covered by IBA1 staining relative to WT (Fig. 2a, b), perhaps reflecting subtle, early changes in microglial morphology. While levels were modestly low given the early age, we detected significantly more microglia co-expressing CD68 in KO dentate gyrus compared to that from WT mice (Fig. 2a, c), as well as a higher, albeit more variable, percentage area covered by CD68 in KO mice (Supplementary Fig. 2a, b). TIMP2 deletion did not affect the total number of microglia (Supplementary Fig. 2a, c).

We next examined if these changes persist or worsen at later ages, given the well-established age-dependent changes in microglia that occur in the hippocampus^34^. At 6–7 months of age, we found that while the total number of microglia was not affected by TIMP2 deletion (Supplementary Fig. 2d, e), the percentage of DG area occupied by IBA1 immunoreactivity was significantly higher in the DG of TIMP2 KO compared to WT DG (Fig. 2d, e). To further characterize this change, we performed Imaris-based morphology analyses on microglia from TIMP2 KO and WT mice. KO microglia exhibited increased surface area/volume, increased oblate ellipticity, and unchanged branch intersections (Supplementary Fig. 2f–k), suggesting overall a modest shift from resting towards reactive and pro-inflammatory microglia^35–37^, and consistent with the increased IBA1^+^ coverage in the hippocampus we detected. We also detected an elevation in the number of IBA1^+^CD68^+^ cells in the DG of KO mice compared to WT, suggesting an altered activation state (Fig. 2d, f).

To examine whether loss of TIMP2 is associated with changes in microglial function, we performed confocal imaging of sections stained with antibodies for IBA1, CD68, and synaptic marker VGLUT1, followed by Imaris-3D reconstruction, to visualize lysosome-associated synaptic puncta within individual microglia. TIMP2 deletion was associated with significantly reduced volume of VGLUT1^+^ puncta within microglia lysosomes in the DG relative to WT controls (Fig. 2g, h), pointing to impaired phagocytic function. In inflammatory contexts, microglia release cytokines and other factors into the extracellular space, affecting the cellular microenvironment of the brain. To further probe altered function in KO hippocampus, we next evaluated how deletion of TIMP2 alters the extracellular milieu. Conventional tissue-based assessment of cytokines and chemokines do not accurately reflect the composition of the brain interstitial fluid (ISF) given loss of compartmental barriers and effects of extraction methods on sensitivity. This approach is also limited to postmortem sampling, which may not sufficiently capture the dynamics of immune changes. To circumvent these issues, we employed high molecular-weight cut-off (HMWCO; 1-MDa) in vivo microdialysis to sample the hippocampal ISF of awake and freely moving mice (Fig. 2i)^38,39^. This technique has only been applied rarely to measure brain immune proteins^39^, which are typically present in the brain ISF at very low levels. To confirm this method’s potential to probe immune-related proteins in the brain ISF, we administered a single systemic injection of lipopolysaccharide (LPS; 5 mg/kg), an established pro-inflammatory stimulus^40^, as a proof-of-concept approach in a 5XFAD model of amyloid pathology that should exhibit high baseline cytokine and chemokine levels in the brain. Following microdialysis probe insertion into the hippocampus and a subsequent equilibration period to allow levels of ISF proteins to stabilize, as previously described^41,42^, samples were collected over a baseline period before LPS treatment. ISF samples were collected 10 h following LPS, and pre- and post-LPS samples were quantified using an exploratory proximity extension assay (PEA)-based panel of cytokines, chemokines, and related proteins (Supplementary Fig. 2l, m). We measured a number of expected pro-inflammatory cytokines and chemokines that were significantly elevated in hippocampal ISF following LPS treatment compared to the baseline period, including IL1β, CCL5, TNF, IL6, as well as anti-inflammatory protein IL-10, which has been shown to be released by microglia following LPS as a means to resolve the inflammatory response^43^ (Supplementary Fig. 2m). Having successfully dialyzed and detected immune-related proteins using this method, we sought to assess the impact of TIMP2 deletion on the hippocampal extracellular milieu. 2–3-month-old WT and KO mice were subjected to the HMWCO in vivo microdialysis paradigm to sample hippocampal ISF during a stable, steady state period of 24 h for measurement of proteins in pooled ISF samples (Fig. 2j). Interestingly, deletion of TIMP2 increased a number of proteins associated with inflammation in the hippocampal ISF (Fig. 2k), including chemokine CCL20 that we had found to be elevated following LPS stimulation (Supplementary Fig. 2m), and EDA2R, a member of the tumor necrosis factor receptor superfamily whose levels correlate with increased age^44^. EDA2R cytokine signaling contributes to inflammation through activation of the NF-κB pathway. TIMP2 deletion also increased hippocampal ISF levels of HGF, a pleiotropic cytokine that modulates the inflammatory response through NF-κB signaling that is also elevated in CSF of AD subjects^45,46^. Several proteins known to be upregulated under neurotoxic conditions as a means to limit damage were also found to be upregulated in TIMP2 KO ISF, including EPO, GFRA1, TGFA^47,48^, as was the lysosomal serine protease TPP1 that is associated with phagocytic function^49,50^. As expected^26^, we also found that loss of TIMP2 increases hippocampal ISF levels of Matrilin-2 (MATN2), an ECM protein that signals through TLR4 to induce proinflammatory genes in macrophages to promote axonal damage^51^. Together, these changes in the hippocampal extracellular environment may reflect a more pro-inflammatory, debris-rich environment relative to that in the WT hippocampus.

### Microglial TIMP2 regulates markers associated with activation and senescence

**Fig. 3:**
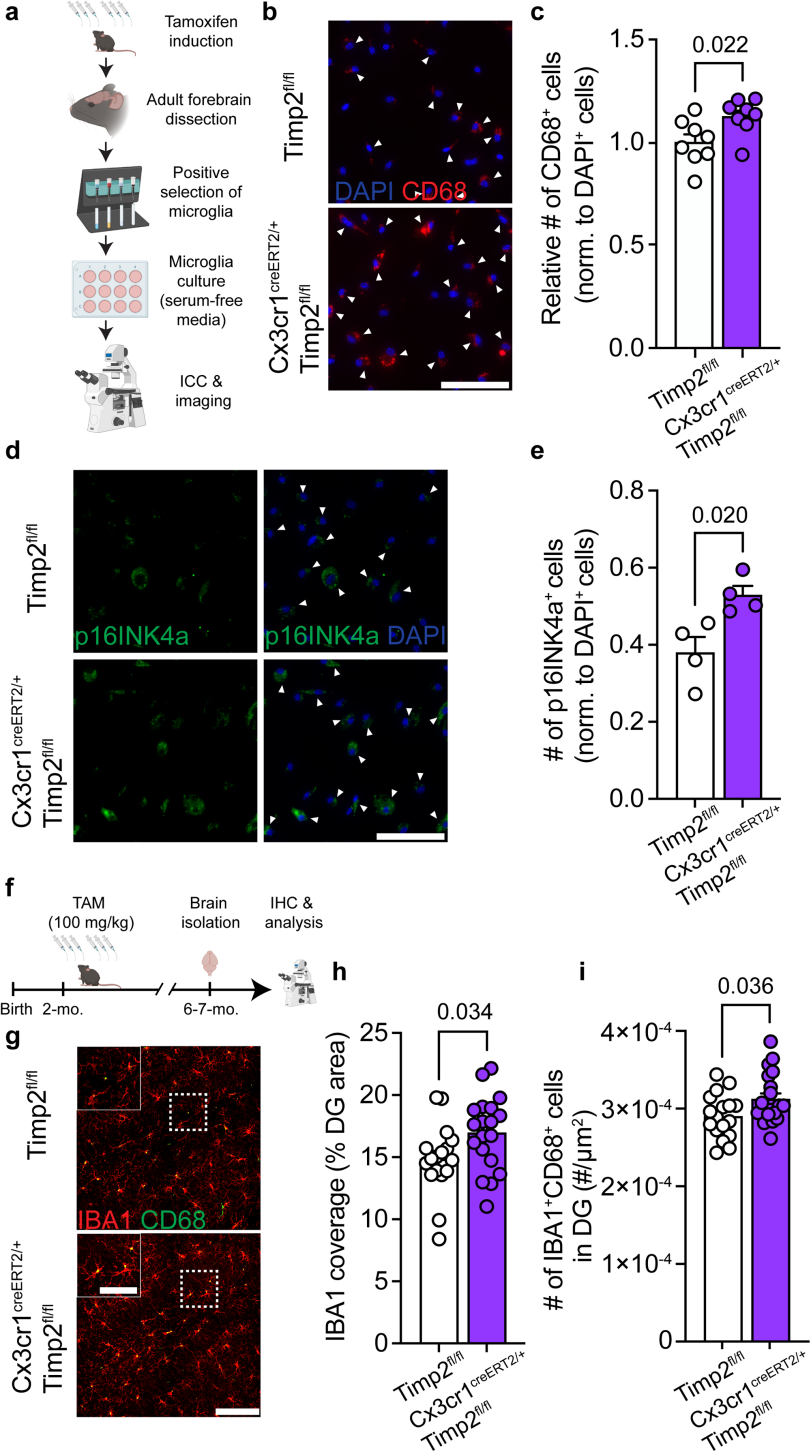
Removal of microglial TIMP2 increases activation- and senescence-associated phenotypes. **a** Schematic diagram of tamoxifen-inducible microglial deletion of TIMP2 in mice and subsequent isolation of adult microglia, culturing, and imaging. **b** Representative images from primary microglia cultures of Timp2^fl/fl^ and Cx3cr1^CreERT2/+^;Timp2^fl/fl^ littermates stained by ICC with DAPI and anti-CD68 antibody (arrowheads indicate CD68^+^ cells; scale bar = 100 μm) with corresponding **c** quantification of the number of CD68^+^ cells normalized to total cell number (DAPI^+^) (*N* = 8 mice per group; two independent experiments combined; 5–6-month-old mice; sex-matched; mean ± SEM). **d** Representative images from primary microglia cultures of Timp2^fl/fl^ and Cx3cr1^CreERT2/+^;Timp2^fl/fl^ littermates stained by ICC with DAPI and anti-p16INK4a antibody (arrowheads indicate p16INK4a^+^ cells; scale bar = 100 μm) with corresponding **e** quantification of the number of p16INK4a^+^ cells normalized to total number of cells (DAPI^+^) (*N* = 4 mice per group, 7-month-old mice, sex-matched; mean ± SEM). **f** Timeline of Cre induction paradigm with tamoxifen in Timp2^fl/fl^ control and Cx3cr1^CreERT2/+^;Timp2^fl/fl^ mice and downstream processing of tissue. **g** Representative confocal images of DG of 6–7-month-old Timp2^fl/fl^ (*N* = 17) and Cx3cr1^CreERT2/+^;Timp2^fl/fl^ (*N* = 19) sex-matched mice stained with anti-IBA1 and anti-CD68 antibodies (scale bar = 100 μm and magnified inset, scale bar = 50 μm), with corresponding **h** quantification of DG area covered by anti-IBA1 staining and **i** the number of IBA1^+^CD68^+^ cells normalized to area. Mean ± SEM; two-tailed Student’s *t*-test. Source data provided as Source Data file. Created in BioRender: Philippi, S. (2026) https://BioRender.com/jlz74wj.

Our findings suggest that loss of TIMP2 alters microglial state and contributes to an inflammatory extracellular environment, yet it remains unclear whether these differences are mediated by the pool of TIMP2 we found is produced by microglia (Fig. 1b, c, l, m). To explore the contribution of microglial sources of TIMP2 to the microglial phenotypes associated with global TIMP2 deletion, we used a *Timp2*^fl/fl^ model we generated^26^ and crossbred them with *Cx3cr1*^CreERT2/+^ mice^52^ to inducibly delete *Timp2* in *Cx3cr1*-expressing cells (e.g., microglia). Using a previously established adult microglia isolation paradigm^53^ (Fig. 3a) that successfully yielded pure adult microglia in culture (Supplementary Fig. 3a–c), we first induced Cre-mediated recombination and then confirmed efficient deletion of microglial TIMP2 (Supplementary Fig. 3d, e). To examine how TIMP2 deletion in microglia affects expression of a canonical phagolysosomal marker, we imaged primary adult microglia from 5–6-month-old mice and examined CD68 staining. Targeting microglial expression of TIMP2 significantly increased the number of cells expressing CD68 (Fig. 3b, c). Given the association between deleterious microglial phenotypes and senescence^54,55^ and hints of this from bulk RNAseq (Supplementary Fig. 1k), we also quantified the number of primary microglia from 7-month-old mice expressing senescent marker p16INK4a. This experiment revealed a greater number of p16INK4a^+^ microglia from *Cx3cr1*^CreERT2/+^;*Timp2*^fl/fl^ mice compared to that observed from *Timp2*^fl/fl^ control mice (Fig. 3d, e), Other senescence-associated markers, including p21 and senescence-associated β-galactosidase (SA-β-gal), were altered in a similar fashion (Supplementary Fig. 3f–i). We next examined the number of IBA1^+^CD68^+^ cells in the DG of 6–7 month-old *Cx3cr1*^CreERT2/+^;*Timp2*^fl/fl^ mice or *Timp2*^fl/fl^ control mice that had received tamoxifen (Fig. 3f). We observed an increase in the extent of IBA1 staining covering the DG, an increase in the number of CD68^+^ microglia (Fig. 3g–i), and an increase in the percentage of DG area stained by CD68 (Supplementary Fig. 3j–l), with no changes in total IBA1+ cell number. These findings suggest that removal of the microglial pool of TIMP2 leads to microglial changes that are consistent with those observed in the setting of global TIMP2 deletion.

### Microglia contribute to extracellular pools of TIMP2 and its removal modifies myelin phagocytosis

**Fig. 4:**
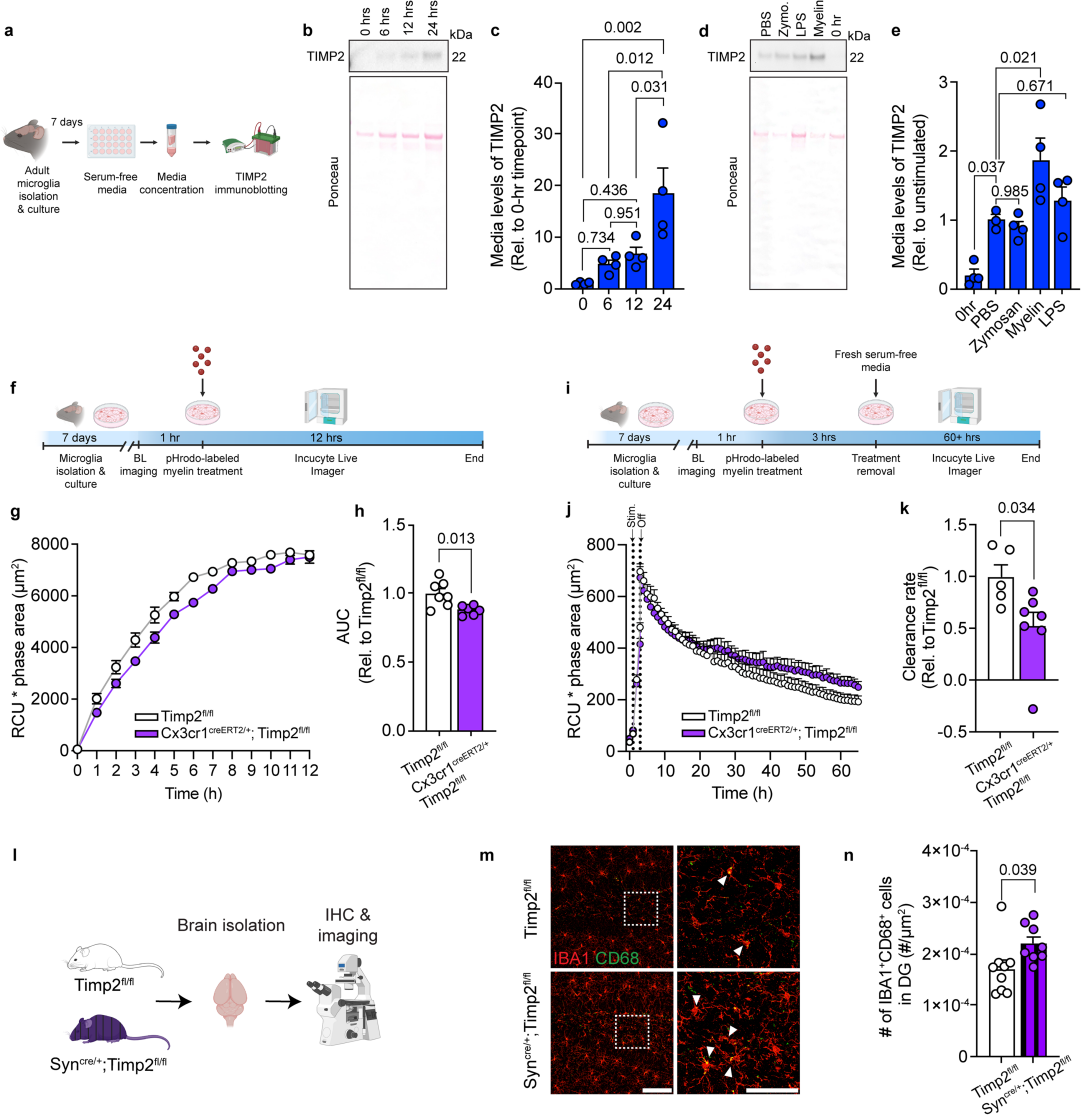
TIMP2 is released by microglia and alters microglial response to myelin. **a** A schematic diagram showing the timeline of primary microglia culturing from adult mice and media assay for TIMP2 immunoblotting experiments. **b** Representative TIMP2 immunoblotting from conditioned serum-free media from primary microglia isolated from 2-month-old WT mice, including Ponceau stain (bottom), with corresponding **c** TIMP2 band intensity quantification (*N* = 4 mice per timepoint; 4 independent experiments combined, with a full timecourse for each experiment; sex-matched; mean ± SEM). **d** Representative TIMP2 immunoblotting from conditioned serum-free media from primary microglia isolated from 2-month-old WT mice at baseline (0 h with no treatment) or 12 h following treatment with either PBS, zymosan (10 µg/mL), myelin (10 µg/mL), or LPS (100 ng/mL), with corresponding **e** TIMP2 band intensity quantification (*N* = 4 mice per treatment (*N* = 3 mice for PBS); 4 independent experiments combined, with all treatments representing an experiment; sex-matched; mean ± SEM). **f** Schematic diagram of treatment of adult primary microglia with pHrodo-labeled myelin in serum-free media and subsequent Incucyte imaging to assess uptake. **g** Timecourse from an experiment with total integrated density (red object mean intensity [RCU, red, *x* average phase object area (cell, μm^2^)]) of pHrodo-labeled myelin signal in primary microglia cultured from tamoxifen-treated 5–7-month-old Cx3cr1^CreERT2/+^;Timp2^fl/fl^ and Timp2^fl/fl^ control mice, with **h** uptake quantification by area under the curve (AUC; *N* = 7 TIMP2^fl/fl^, *N* = 6 Cx3cr1^CreERT2/+^;Timp2^fl/fl^ sex-matched mice; 2 independent experiments combined; mean ± SEM). **i** Schematic diagram of the treatment of adult primary microglia with pHrodo-labeled myelin in serum-free media to assess clearance. **j** Timecourse from an experiment with total integrated density (RCU [red calibrated units]) *x* average phase object area (cell, μm^2^)) of pHrodo-labeled myelin signal in primary microglia cultured from 5–7-month-old tamoxifen-treated Cx3cr1^CreERT2/+^;Timp2^fl/fl^ and Timp2^fl/fl^ control mice, with **k** clearance rate quantification (from slopes of regressions), represented relative to Timp2^fl/fl^ control (*N* = 5 TIMP2^fl/fl^, *N* = 7 Cx3cr1^CreERT2/+^;Timp2^fl/fl^ sex-matched mice; 2 independent experiments combined; mean ± SEM). **l** Schematic diagram of neuron-specific TIMP2 deletion using Syn^Cre/+^;Timp2^fl/fl^ and Timp2^fl/fl^ mice for confocal imaging experiments. **m** Representative confocal images of DG from 2–3-month-old Syn^Cre/+^;Timp2^fl/fl^ (*N* = 8) and Timp2^fl/fl^ (*N* = 9) female mice stained with anti-IBA1 and anti-CD68 antibodies (left; scale bar = 100 μm) and magnified inset (right; scale bar = 50 μm; white arrowheads indicate IBA1^+^CD68^+^ cells) with corresponding **n** quantification of IBA1^+^CD68^+^ cell number normalized to DG area. Mean ± SEM; One-way ANOVA with Tukey’s post hoc test (**c**); One-way ANOVA with Dunnett’s post hoc test (**e**); Two-tailed Student’s *t*-test (**h**, **k**, **n**). Source data provided as Source Data file. Created in BioRender: Philippi, S. (2026) https://BioRender.com/2fr7zt2.

TIMP2 is present at high levels in the extracellular space of the hippocampus^26^, likely reflecting contributions from various sources, including from neurons and microglia. Our data show that microglia express TIMP2 at both transcript and protein levels, yet it remains unclear whether microglia release TIMP2, where it can potentially alter microglial response to pathological debris, and whether targeting microglial TIMP2 affects associated phenotypes. To explore these questions, we isolated and cultured adult WT microglia from 2-month-old mice to examine TIMP2 release into the media across various timepoints in serum-free media, which was necessary given the high levels of endogenous TIMP2 present in plasma/serum, especially at early life stages^16^ (Fig. 4a). Immunoblotting analysis on media from these cultures revealed that microglia release TIMP2 over time (Fig. 4b, c). We next asked whether levels of TIMP2 released into the media are modulated by exposure to various biological substrates at established doses^56^ (zymosan, 10 µg/mL; LPS, 100 ng/mL), including substrates commonly encountered within the brain (myelin, 10 µg/mL). 12 h following stimulation with these substrates, we measured TIMP2 levels in the conditioned media by immunoblotting. While zymosan stimulation did not change TIMP2 levels in the media relative to unstimulated conditions, LPS slightly increased levels, though this did not reach statistical significance, and myelin stimulation robustly increased (~two-fold) TIMP2 release relative to unstimulated conditions (Fig. 4d, e).

Given that the brain accumulates abundant myelin debris with age^57^, the increase in TIMP2 in response to myelin stimulation motivated us to examine whether microglial TIMP2 modulates cellular response to myelin. We isolated adult microglia from 5–7-month-old tamoxifen-treated *Cx3cr1*^CreERT2/+^;*Timp2*^fl/fl^ mice and *Timp2*^fl/fl^ control mice and performed a myelin phagocytosis assay in which myelin was conjugated with pHrodo red, a pH-sensitive dye that fluoresces in the acidic endosomal/lysosomal compartment that was monitored over time using a live-cell imaging system (Fig. 4f)^56^. ‘Area under the curve’ analysis revealed significantly lower uptake of myelin in *Cx3cr1*^CreERT2/+^;*Timp2*^fl/fl^ microglia over a 12-h period compared to the uptake observed in control microglia (Fig. 4g, h), whereas pHrodo-labeled zymosan had no significant effect (Supplementary Fig. 4a–c). We next assessed the rate of myelin clearance by treating new cultures from the treated groups of mice with myelin, followed by withdrawal of the treatment with fresh media to measure the rate of myelin elimination (Fig. 4i). We observed a lower rate of myelin clearance in *Cx3cr1*^CreERT2/+^;*Timp2*^fl/fl^ microglia relative to *Timp2*^fl/fl^ control microglia (Fig. 4j, k). Together, these results suggest that deletion of microglial TIMP2 impairs microglial phagocytosis of myelin.

We previously reported that neuron-derived TIMP2 released into the brain extracellular space is critical for hippocampal plasticity^26^. We next asked whether this additional extracellular source from neurons can regulate microglial state by creating *Syn*^Cre/+^;*Timp2*^fl/fl^ and *Timp2*^fl/fl^ mice to target neuronal TIMP2 expression^26^ and then performing confocal imaging to evaluate microglia using IBA1 and CD68 markers (Fig. 4l). Similar to our findings demonstrating that targeting the microglial pool of TIMP2 affects microglial state (Fig. 3i), neuronal TIMP2 deletion significantly increases the number of IBA1^+^CD68^+^ cells in the DG (Fig. 4m, n), while the total number of microglia was not affected by TIMP2 deletion (Supplementary Fig. 4d, e). Together, our findings indicate that microglia are responsive to the modulation of extracellular levels of TIMP2, regardless of whether the source is from microglia or neurons.

### Systemic TIMP2 treatment reduces age-associated microglial phenotypes and modulates microglial state

**Fig. 5:**
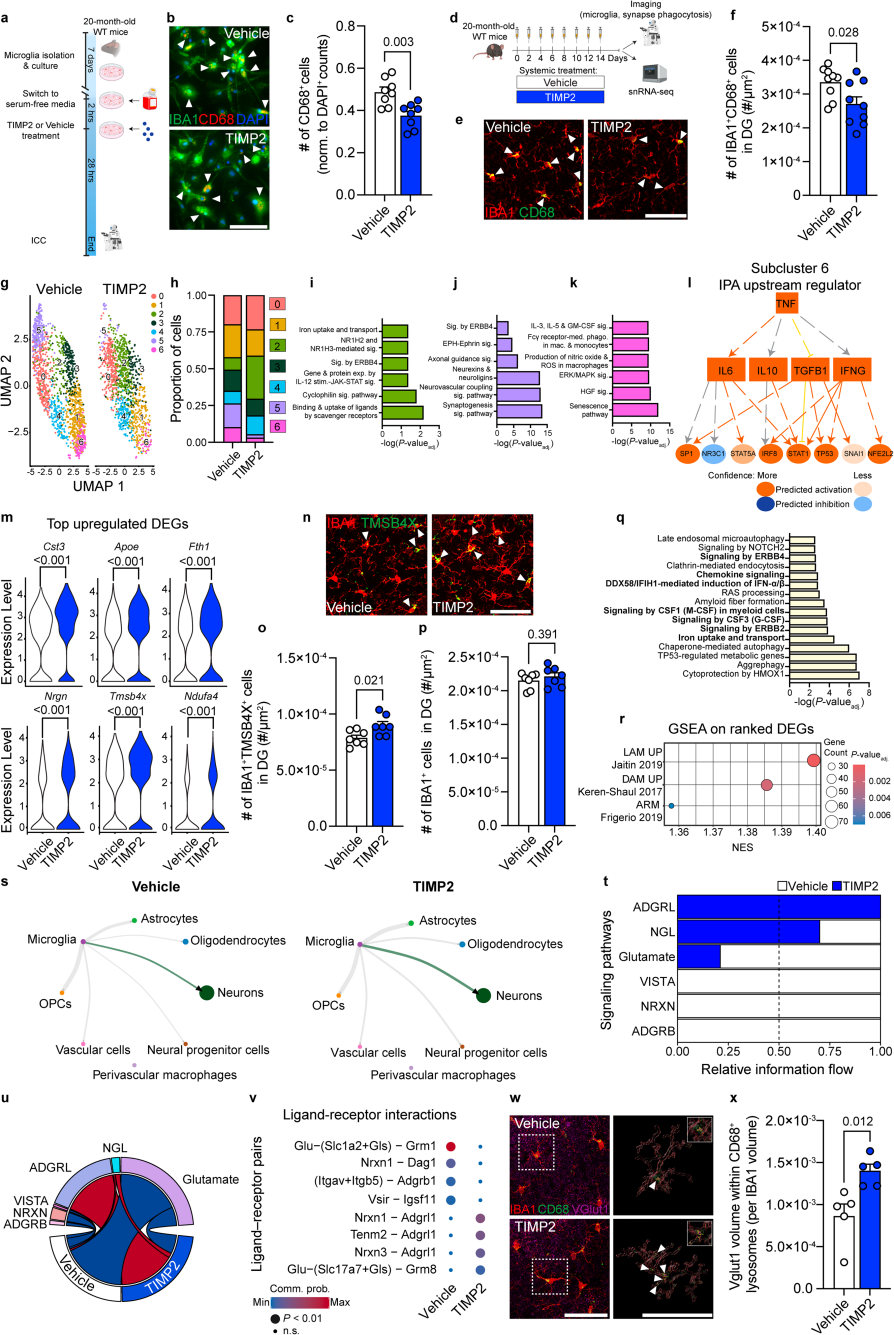
Youth-associated TIMP2 alters microglia in aged mice. **a** A schematic of the experimental timeline of microglial isolation from 20-month-old WT mice, followed by incubation with TIMP2 or vehicle. **b** Representative images from cultures of aged WT primary microglia stained with anti-IBA1 and anti-CD68 antibodies by ICC (arrowheads indicate IBA1^+^CD68^+^ cells; scale bar = 100 μm), with corresponding **c** quantification of the number of CD68^+^ cells normalized to total cell number (*N* = 8 mice per treatment group, 2 independent experiments combined; sex-matched; mean ± SEM). **d** Schematic diagram showing the paradigm of systemic treatment with recombinant TIMP2 or vehicle in 20-month-old WT mice, followed by downstream analyses. **e** Representative confocal images of DG from 20-month-old WT mice treated systemically with TIMP2 or vehicle, stained with anti-IBA1 and anti-CD68 antibodies (scale bar = 100 μm; white arrowheads indicate IBA1^+^CD68^+^ cells), with corresponding **f** quantification of the number of IBA1^+^CD68^+^ cells normalized to DG area (*N* = 9 male mice per group; mean ± SEM). **g** Uniform Manifold Approximation and Projection (UMAP) plot of hippocampal microglial nuclei (2831) isolated from 20-month-old WT mice treated systemically with vehicle or TIMP2 (*N* = 4 male mice per group combined as 2 samples per group). **h** Proportion of nuclei across microglial subclusters for each treatment group with corresponding **i** significant (*P*-value_adj._ < 0.05; Benjamini-Hochberg) canonical IPA pathways for marker genes significantly upregulated in subcluster 2 (from one-tailed Wilcoxon Rank Sum *P*_adj_-values (Bonferroni-adjusted)), **j** subcluster 5, and **k** subcluster 6. **l** IPA upstream regulator network based on subcluster 6 marker genes, with predicted relationships for target genes. **m** Violin plots of normalized gene expression values from selected top DEGs (*P*-values from DESeq2, two-sided Wald test, Benjamini-Hochberg-adjusted). **n** Representative confocal images of DG from 20-month-old male mice treated with TIMP2 or vehicle and stained with anti-IBA1 and anti-TMSB4X antibodies (scale bar = 50 µm; arrowheads indicate IBA1^+^TMSB4X^+^ cells), with **o** quantification of the number of IBA1^+^TMSB4X^+^ cells, normalized to DG area, and **p** total number of IBA1^+^ cells normalized to DG area; *N* = 7 mice per group; mean ± SEM. **q** Significant canonical pathways from IPA on microglial DEGs (*P*-value_adj._ < 0.05, Benjamini-Hochberg) from TIMP2-treated vs. vehicle-treated aged mice. **r** Dot plot from GSEA performed on ranked gene lists derived from microglia DEGs obtained from a two-tailed Wald test. Significantly enriched gene sets derived from published transcriptomic states are shown with corresponding Normalized Enrichment Score (NES), with point size representing the number of overlapping genes and color indicating *P*-value_adj_ (Benjamini-Hochberg). **s** Cell-Chat communication network between microglia and all cell types, with line thickness indicating number of interactions from microglia (“sender”) to other cell types (“receiver”), highlighting 'microglia-neurons' in green. **t** Relative information flow chart showing relative contributions for each microglia-neuron signaling pathway for vehicle (white) and TIMP2 (blue) groups, with **u** Circos diagram depicting share of mean communication probabilities by group (TIMP2 vs. Vehicle); “red” or “blue” reflects net difference in probability being higher or lower with TIMP2 treatment, respectively. **v** Bubble plot of ligand-receptor pairs for altered pathways in “u”, with size reflecting significance (*P* < 0.01; n.s. denotes *P* > 0.05, computed from one-sided permutation test) and color reflecting communication probability magnitude for TIMP2 and Vehicle. **w** Representative confocal images (left) of DG from 20-month-old WT mice treated systemically with TIMP2 or vehicle, stained with anti-IBA1, anti-CD68, and anti-Vglut1 antibodies (scale bar = 50 μm), with Imaris-based reconstruction (right) of insetted microglial volume (red), containing engulfed Vglut1^+^ puncta (purple with yellow outlines indicated by white arrowheads) within microglial lysosomes (green) (scale bar = 50 μm). **x** Quantification of the volume of engulfed Vglut1^+^ signal within CD68^+^ volume, normalized to microglia volume. Values represent *N* = 5 mice per group; mean ± SEM; two-tailed Student’s *t*-test in panels (**c**, **f**, **o**, **p**, **x**). Source data provided as Source Data file. Created in BioRender: Philippi, S. (2026) https://BioRender.com/osbivoa.

Given our data suggesting that extracellular sources of TIMP2 regulate microglial state in vivo, we next asked whether extracellular application of TIMP2 to aged primary microglia could directly affect microglial phenotypes observed in our KO models, independent of regulation from other cell types. We first isolated microglia from 20-month-old WT mice and evaluated whether application of TIMP2 altered the number of IBA1^+^CD68^+^ cells in the culture (Fig. 5a). Treating aged primary microglia with TIMP2 reduced the number of CD68^+^ cells relative to control conditions (Fig. 5b, c), suggesting that TIMP2 can directly alter microglial state independent of its known effects on other CNS cell types^24,26,58^. Based on our previous results demonstrating that TIMP2 enters the brain from blood following systemic injection^16^, we next asked whether systemic treatments with TIMP2 in aged mice can regulate microglial state and function. Using the same dosing paradigm we previously showed improves learning and memory in aged mice (Fig. 5d)^16^, we injected aged WT mice systemically with TIMP2 (50 μg/kg), and isolated brains to evaluate changes in microglia after ~2.5 weeks of injections. Systemic TIMP2 treatment reversed the consistent increase in microglial activation observed in our KO models, as reflected by the decreased number of IBA1^+^CD68^+^ cells in the DG of TIMP2-treated aged mice compared to vehicle-treated aged mice (Fig. 5e, f). We also detected a decreased percentage of DG area occupied by CD68 staining, whereas the treatment did not affect the total number of IBA1^+^ cells (Supplementary Fig. 5a–c). Changes in morphology were modest, with increased branch intersections, a slight increase in prolate ellipticity, and no changes in volume, consistent with a mixed remodeling phenotype that is suggestive of a protective, responsive state^59–61^. (Supplementary Fig. 5d–g).

To characterize how aged microglia respond to TIMP2 treatment at the transcriptomic level, we applied a single-nuclei RNA-sequencing (snRNA-seq) pipeline on hippocampi isolated from a new cohort of vehicle- and TIMP2-treated 20-month-old mice, based on an established method^62^. Following quality-control measures to remove ambient RNA, doublets, and poor-quality nuclei, we obtained 60,436 nuclei for downstream analysis in which nuclei from each sample were integrated and clustered using uniform manifold approximation and projection (UMAP) (Supplementary Fig. 5h). Combining the use of ScType for automated cell-type identification^63^ and thorough interrogation of top marker genes for each cluster, we identified CNS cell types expected for the hippocampus (Supplementary Fig. 5h, i). We next subclustered cells identified as microglia (2831 nuclei; Fig. 5g) and noted significant shifts in the proportion of cells in subclusters 2, 5, and 6 in mice treated systemically with TIMP2 compared to vehicle-treated mice (Fig. 5h, Supplementary Fig. 5j). To identify what processes characterize these TIMP2-shifted subclusters, we performed IPA on the significant genes defining these subclusters and examined expression levels of top marker genes. TIMP2 treatment increased the proportion of microglia in subcluster 2, a cluster characterized by the highest expression of *Apoe*, a gene that regulates phagocytosis and lipid uptake^29,64^ (Supplementary Fig. 5k), as well as by iron handling (“uptake and transport”) and immune response processes (“cyclophilin signaling”) (Fig. 5i). TIMP2 treatment shifted subcluster 1, albeit modestly (Fig. 5h, Supplementary Fig. 5j), which was characterized by genes relating to phagosome formation and signaling in myeloid cells (Supplementary Fig. 5l). TIMP2 treatment significantly reduced the proportion of microglia in subcluster 5 (Fig. 5h, Supplementary Fig. 5j), a cluster defined by “axon guidance signaling”, “synaptogenesis signaling”, and “neurexins and neuroligins” (Fig. 5j). This subcluster, along with 2 other microglial subclusters with neuron-associated genes, also had highly enriched expression of *Gria1* (Supplementary Fig. 5k), a gene linked to microglial-neuron interactions^65,66^. Notably, TIMP2 treatment resulted in a significant decrease in proportion of subcluster 6 microglia (Fig. 5h, Supplementary Fig. 5j), which were defined by pathways associated with production of “nitric oxide and reactive oxygen species (ROS) in macrophages”, “senescence”, “Fcγ receptor-mediated phagocytosis”, “IL-3, IL-5, and GM-CSF signaling”, and “HGF signaling” (Fig. 5k). Using the top subcluster 6 marker *Tlr7* (Supplementary Fig. 5k), we further confirmed that TIMP2 treatment decreased the number of TLR7-expressing microglia in DG, while not changing total IBA1+ cell counts (Supplementary Fig. 5m–o). Further analysis of subcluster 6 revealed “TNF” as a top upstream regulator (Fig. 5l), activating proinflammatory^67–69^ and senescence-associated^70^ pathways through IL6, IFNG, STAT1/STAT5A, IRF8, and TP53, as well as IL10 as a counter-regulatory response protein^71^, together suggesting that TIMP2 treatment of aged mice reduces the proportion of microglia associated with a pro-inflammatory and senescent-like state.

Given these shifts in microglial subclusters following TIMP2 treatment, we next aimed to compare overall microglial gene expression changes following TIMP2 treatment. We found that TIMP2 treatment resulted in differentially expressed genes (DEGs; 196 upregulated, 68 downregulated) in the overall microglia cluster. *Cst3* and *Apoe* were the two most significantly upregulated genes (Fig. 5m). *Apoe* is a well-characterized regulator of the DAM transcriptional state^29^ and is upregulated in microglia phagocytosing apoptotic neurons^64^, while *Cst3* is localized in the lysosome, where it regulates lysosomal homeostasis^72^. We also observed a change in ferroptosis-associated *Fth1*^73^ and genes linked to metabolism and inflammation, including *Ndufa4*^74^ and *Tmsb4x*, which negatively regulates NFκB signaling^75^ (Fig. 5m). Consistent with some of these changes, we detected significantly higher numbers of microglia in DG expressing TMSB4X in aged mice treated with TIMP2 compared to control (Fig. 5n–p). Pathway analysis on the overall DEGs reflected changes in cytokine/chemokine and interferon signaling, as well as altered uptake processes (ferroptosis), and potential changes in microglia-neuron interactions (ERBB4 and ERBB2 signaling) (Fig. 5q). We performed pre-ranked Gene Set Enrichment Analysis (GSEA)^76^ to determine if genes, ranked by signed fold-change*-log_10_*P*-value_adj_, were enriched within gene sets previously associated with different microglial states, including disease-associated microglia (DAM)^29^, lipid-associated macrophages (LAM)^77^, which was identified as a protective state in adipose tissue of mice on a high-fat diet^77^, lipid-droplet-accumulating microglia (LDAM)^12^, activated response microglia (ARM)^78^, interferon-related^79^ and homeostatic microglia^78^. We found that TIMP2 treatment resulted in positive enrichment in LAM, DAM, and ARM gene sets in microglia (Fig. 5r, Supplementary Fig. 5p), supporting a putative enrichment in genes associated with protective response to damage. WGCNA performed on the microglial cluster genes highlighted two significant modules: a downregulated brown module associated with pathways that include “cell activation” and “regulation of immune cell process” (Supplementary Fig. 5q, r), and an upregulated module associated with “cellular homeostasis”, “projection morphogenesis”, and “fiber organization” pathways (Supplementary Fig. 5q, s). We next used CellChat to explore TIMP2-mediated changes in communication between microglia and other CNS cell types. Focusing on microglia-neuron communication, TIMP2 treatment was associated with an increased number of ligand-receptor interactions (Fig. 5s) compared to control. Further analysis showed altered information flow (relative communication probabilities) for immune-, synapse-, and adhesion-related pathways, with increased information flow after TIMP2 treatment in ADGRL and NGL pathways, and reduced flow for Glutamate, VISTA, NRXN, and ADGRB pathways (Fig. 5t). Given the large TIMP2-mediated changes in communication probabilities, especially for ADGRL and Glutamate pathways (Fig. 5u), we extracted ligand-receptor pairs for these pathways, revealing significantly altered interaction probabilities for Glu-Grm1 and Nrxn1-Adgrl1 pairs associated with these pathways (Fig. 5v). Overall, these results are suggestive of altered interactions in several microglial pathways affecting neurotoxicity^80^ and synapse homeostasis^81^, as well as pathways for ADGRL, part of the larger family of adhesion GPCRs linked to phagocytosis^82,83^.

### Systemic TIMP2 treatment restores phagocytic capacity in aged microglia

Phagocytic capacity of microglia is diminished in aging and in proinflammatory states^8,9,12,84^, and targeting this process may facilitate effective clearance of dead cells or debris^9,10,85^. Given the TIMP2-induced changes observed in microglial phagocytosis-related pathways and in putative microglial-synapse interactions in snRNA-seq experiments, we evaluated how systemic TIMP2 treatment of 20-month-old mice affects the ability of microglia to phagocytose synaptic material in the DG. We hypothesized that TIMP2 treatment elevates microglial phagocytosis in aged DG, especially given our results showing that *Timp2* deletion reduced myelin phagocytosis in vitro (Fig. 4g, h) and reduced the number of VGLUT1 puncta within microglial lysosomes in the hippocampus (Fig. 2g, h). To evaluate microglial phagocytosis in aged mice following TIMP2 or vehicle treatment (Fig. 5d), we performed confocal imaging of sections stained with antibodies for IBA1, CD68, and synaptic marker VGLUT1, followed by Imaris 3D reconstruction to visualize lysosome-associated synaptic puncta within microglia. We found that TIMP2 treatment increased the volume of VGLUT1^+^ puncta within microglia lysosomes in the DG relative to vehicle treatment (Fig. 5w, x), reflecting enhanced phagocytic function of a process typically diminished with aging^8,12^. Supporting this result using a differing physiological substrate (Supplementary Fig. 5t), we found significantly reduced phagocytosis of pHrodo-labeled myelin by primary microglia from aged mice treated with TIMP2 compared to control (Supplementary Fig. 5u, v).

## Discussion

We define a role for the youth-associated protein TIMP2 in regulating microglial state in the context of aging pathology and in normal brain homeostasis. Microglial- and neuronal-specific deletion of TIMP2 yielded deleterious phenotypes of microglia in the hippocampus, effects that were more modest than the larger effect seen with global deletion, as expected, given the differing contributions to TIMP2 levels. TIMP2 also appeared to modulate phagocytic uptake and clearance of physiological substrates by microglia, including myelin, while regulating the inflammatory state of these cells. Given that various sources of expression contribute to the brain extracellular pool of TIMP2, we explored how systemic supplementation with TIMP2 in aged mice affects microglia, which reversed many phenotypes associated with TIMP2 removal, including changes in microglial state and impaired phagocytosis, while highlighting transcriptomic shifts associated with deleterious microglial phenotypes. Our results highlight that microglia are sensitive to extracellular levels of TIMP2, and that increasing TIMP2 promotes a shift toward a more protective state that may counteract age-associated pathology.

Previous studies suggested that exposure to the systemic environment may affect microglia^18,86^ and that these cells can take up proteins originating in plasma^21,22^, yet little is known of the effects of such proteins on microglia. Specifically, recent work found that labeled plasma proteins are found within microglia of healthy brains in a process that appears to involve a specialized subset of microglia characterized by antigen presentation, as well as high metabolic and phagocytic activity^22^, though it remains unclear whether these processes diverge in the aged brain. A further recent study identified PF4 as a platelet factor that modulates microglial state, though it may not penetrate the brain appreciably and instead acts on peripheral T cells to indirectly modulate the CNS^18,87^, highlighting that microglia likely respond to blood-borne factors through a variety of mechanisms. In the case of TIMP2, previous work found that labeled TIMP2 enters the brain after peripheral delivery in aged mice^16^. Our current data examining the impact of neuronal, microglial, and systemic sources of TIMP2 on microglial phenotypes collectively support the concept that extracellular pools of TIMP2 regulate microglial function.

A growing literature has highlighted roles for TIMP2 in regulating overall hippocampal function by modulating synaptic plasticity mechanisms^16,26^, raising the possibility that some of the effects observed in microglia may reflect, in part, its action on neurons. While this remains a possibility, we find that aged primary microglia treated with TIMP2 recapitulate phenotypes observed in the intact brains of aged mice treated systemically with TIMP2, suggesting that microglia are directly responsive to TIMP2. Based on work showing that interactions between ECM and microglia alter spine dynamics^27^, additional investigation can clarify how the relationship between TIMP2 and microglial function affects ECM homeostasis and whether these changes are directly linked or indirectly affected by microglia. Our Cell-Chat data revealed altered microglia-neuron interactions, including signaling changes related to inflammation and synapse integrity^80,81^, as well as altered communication in adhesion GPCR pathways linked to changes in phagocytosis^82,83^, further supporting a role for TIMP2 in intercellular interactions. In terms of molecular targets for TIMP2, a recent study using a modified TIMP2 construct lacking MMP inhibitory activity was still sufficient to enhance hippocampal plasticity phenotypes, arguing for non-canonical targets for TIMP2 activity^24^ beyond classical interactions with MMPs. Given the many distinct interactions for TIMP2 activity that have been identified^25^ and the diverse cellular targets, future studies should focus on delineating molecular targets for its specific CNS functions.

To further examine how TIMP2 modulates microglial state, we applied a combination of in vivo and in vitro approaches across several ages, which revealed a consistent phenotype of heightened microglial activation in the setting of TIMP2 deficiency that is reversed with TIMP2 supplementation. To characterize the local extracellular environment, we applied an in vivo microdialysis method that permitted sensitive assessment of immune-related proteins, which revealed changes in extracellular factors related to inflammation, lysosomal activity, and cellular stress responses, reflecting an altered neuroinflammatory environment. Interestingly, WGCNA of microglial gene expression revealed opposing changes in broadly similar pathways following TIMP2 deletion versus supplementation models, suggesting shared mechanisms related to activation and structural dynamics, though specific TIMP2-dependent mechanisms across varying contexts remain to be defined. Our transcriptomic analysis of aged microglia from TIMP2-treated mice largely corroborated these roles of TIMP2 as a driver of protective microglial states. In microglia of aged mice treated with TIMP2, we find enrichments in sets of genes resembling the LAM^77^, DAM^29^, and ARM^78^ states associated with protective macrophage/microglia function. DAM and ARM states are both associated with protective responses to aging and AD pathology^29,78^ while being characterized by elevated *Apoe* expression, a top upregulated gene in TIMP2-treated microglia in our dataset. Several studies have demonstrated that these states, and induction of genes associated with these states, are necessary for microglia to appropriately respond to damage^29,64,78,88,89^. We also found significant enrichment in the LAM geneset in TIMP2-treated microglia, which has been argued to be a protective myeloid state in adipose tissue of mice on a high-fat diet^77^. These macrophages are involved in phagocytosis, lipid catabolism, and energy metabolism^77^, largely fitting with our observed upregulation of genes related to phagocytosis, metabolism, and modulation of inflammatory response in microglia from TIMP2-treated mice. The enrichment in genes associated with LAM, DAM, and ARM states with TIMP2 treatment may reflect a shared change in microglial response to lipid-rich damage, including the myelin debris encountered by aged microglia, possibly a protective response to aging that is bolstered with TIMP2 treatment. Corroborating the protective effects, we see a corresponding loss of microglial subpopulations characterized by deleterious pathways in microglia from TIMP2-treated mice, including senescence, production of nitric oxide and ROS, and pro-inflammatory programs driven by TNF. A previous in vitro study using the BV2 cell line suggested that TIMP2 may have anti-inflammatory roles, including production of IL10 and reduction of pro-inflammatory cytokines following LPS stimulation^90^, supporting our in vivo observations in microglia.

A consistent phenotype we observed across experiments modulating TIMP2 levels is the shift in microglial state and changes in phagocytic capacity. We show that removing TIMP2 increases the number of microglia expressing the lysosomal-associated marker CD68, while reducing uptake and removal of myelin, together supporting that TIMP2 regulates how physiological material is removed from the extracellular space. Interestingly, by sampling the brain extracellular space of mice lacking TIMP2, we detected elevations in the lysosomal serine protease TPP1, perhaps reflecting a response to aberrant debris removal by microglia, a phenomenon observed under conditions of lysosomal stress^91,92^. Conversely, TIMP2 treatment of aged mice reduces microglial CD68 and increases phagocytic capacity—a function typically diminished with age and under proinflammatory conditions^8,9,84^. These data dovetail with our transcriptomic data from treated aged mice that reveal upregulation of genes involved in phagocytic processes and metabolism and a shift away from proinflammatory microglial states, perhaps reflecting a more efficient endolysosomal capacity. In addition to the benefit of clearing debris to protect the local brain environment, the process of phagocytosis itself can also inhibit proinflammatory responses^93^, though future work will need to identify the directionality of these changes following treatment with TIMP2 and related factors and the extent to which phagocytosis of varied types of debris is affected and under what contexts, including injury and other models. While microglial CD68 is sometimes interpreted as a proxy for phagocytosis, its level does not always directly correlate with levels of phagocytosis in myeloid cells, as one study found that macrophage phagocytosis was normal in CD68 KO mice^94^. Others have reported increased CD68 alongside reduced phagocytic clearance^95^, similar to our current results, and a recent study showed elevated CD68 in lipid-accumulating microglia with reduced phagocytosis^12^. These scenarios possibly involve the delayed ability of microglia to handle excess debris, where CD68 is upregulated in a compensatory process. Together, this work suggests that TIMP2 modulates the ability of microglia to remove extracellular material, enabling them to more effectively engage in phagocytosis while reducing the inflammatory environment in the setting of age-related pathology.

Our findings demonstrate that TIMP2 modulates microglial state, enhancing phagocytic function and dampening inflammatory activation in the aged brain. By promoting a more protective set of microglial phenotypes, TIMP2 may help counteract the accumulation of debris and chronic inflammation that characterize the aged CNS environment. Given that genetic risk for AD is enriched in genes involved in microglial phagocytosis and endolysosomal function^96^, future studies should explore how TIMP2 influences these pathways in contexts of aging and age-related neurodegenerative conditions. Together, our work positions TIMP2 as a promising candidate for mitigating microglial dysfunction and preserving brain health across the lifespan.

## Methods

### Animals

Animal procedures, care, and handling were performed in accordance with the NIH Guide for Care and Use of Laboratory Animals and with approval from the Icahn School of Medicine at Mount Sinai Institutional Animal Care and Use Committee (#2017-0296). Mice were housed in a temperature- and humidity-controlled facility on a 12-h light:dark cycle, and mice were given ad libitum food and water access. All mice were maintained on a C57Bl/6J background. “Global” TIMP2 −/− (KO) mice were purchased from Jackson Laboratory (#008120), and 5XFAD mice were purchased from MMRRC and subsequently maintained in-house alongside WT littermates. *Timp2*^fl/fl^ mice were generated as previously described^26^ and crossbred with *Cx3cr1*^CreERT2/+*Litt*^ mice or Syn^Cre/+^ lines (Jackson) to permit microglial^52^ and neuronal deletion^26^ of TIMP2, respectively. To induce conditional deletion of microglial *Timp2*, 2-month-old mice were administered 100 mg/kg tamoxifen (Sigma Aldrich Fine Chemicals Biosciences, T5648) in corn oil (Sigma, C8267) 6 times by oral gavage, with at least 48-h separation between gavages^88^. Cre-negative *Timp2*^fl/fl^ littermates were also given tamoxifen as controls. Aged WT C57Bl/6 mice were obtained from the National Institute on Aging aged rodent colony. Male and female mice were used unless otherwise indicated.

### Early postnatal microglia isolation

Using an established protocol^97^, we dissected forebrain from pups (P5-P8) and manually homogenized the tissue by Dounce homogenizer (VWR, KT885300-0002), prior to debris removal using a 20% Percoll gradient (GE Healthcare, 17-0891-02). Microglia were positively selected using CD11b microbeads (Miltenyi Biotec, 130-049-601), LS columns (Miltenyi Biotec, 130-042-401), and a QuadroMACS Separator according to manufacturer instructions. For western blot analysis, cell pellets were collected and frozen at −80 °C until use. For ICC and bulk RNAseq experiments, cells were manually counted with a hemacytometer and Trypan blue staining to assess cell viability. Cells were plated at a density of 200,000 microglia/well in 96-well poly-d-lysine-coated plates (Corning, 354461) in Dulbecco’s modified Eagle’s medium (DMEM) supplemented with 10% FBS (Sigma, F4135, Sigma) and 1% penicillin–streptomycin (Gibco, 15140). Microglia were recovered for a minimum of 2 days in a tissue culture incubator before experiments.

### Adult microglia isolation

Following an established protocol^53^, mice were perfused with ice-cold PBS, cerebellum and olfactory bulbs were removed, and the remaining brain tissue was enzymatically dissociated using the Neural Tissue Dissociation Kit (P) (Miltenyi Biotech, 130-092-628) according to manufacturer instructions. During incubation in the enzymatic buffer, large, medium, and small fire-polished Pasteur pipettes were used to mechanically dissociate tissue. Following filtration and centrifugation, samples were incubated with CD11b microbeads (Miltenyi Biotec 130-049-601) and subsequently separated using LS columns (Miltenyi Biotec 130-042-401) and QuadroMACS Separator using the manufacturer’s instructions. Cells were plated on imaging plates (Cellvis, P96-1.5H-N) or poly-L-Lysine-coated glass coverslips (Electron Microscopy Sciences, 72292-04) in DMEM supplemented with 10% FBS and 1% penicillin–streptomycin. Microglia were allowed to recover for 7 days in a tissue culture incubator before changing media to serum-free conditions for experiments. Wells were excluded from analysis if cultures did not meet viability or quality control criteria (e.g., excessive debris).

### In vitro microglial phagocytosis assays

Following a 7-day recovery period post-isolation, conditioned media from primary microglial cultures was removed and replaced with serum-free media before measurements in the IncuCyte S3 live-cell analysis system (Sartorius) that contained a tissue culture incubator at 37 ºC/5% CO2 for baseline imaging. Cells were treated with pHrodo-labeled myelin^56^ (10 µg/mL) or zymosan (10 µg/mL), generously provided by Dr. Alison Goate’s laboratory, and imaged at various timepoints at 20x using phase contrast and red fluorescent signal. For myelin clearance experiments, following a 3-h incubation with pHrodo-labeled myelin, cells were washed with PBS, and fresh serum-free media was added that did not contain myelin. Total integrated density was calculated by multiplying the average red object mean intensity (RCU, red calibrated units) by the average phase object area (cell, μm^2^) and plotted over time^98^. For uptake and clearance experiments, two independent experiments were conducted with 3 technical replicates per animal, which were averaged.

### In vitro microglial stimulation

To assess TIMP2 secretion with various substrates, cultures of adult WT microglia were stimulated with various substrates in serum-free media at the following concentrations: zymosan 10 µg/mL, myelin 10 µg/mL, LPS 100 ng/mL, or PBS control. 12 h following stimulation, conditioned media were collected and concentrated for immunoblotting. For experiments examining activation of primary microglia from aged mice following TIMP2 treatment, cell media was replaced with serum-free media 2 h prior to incubation with TIMP2 (2 µg/mL, R&D Systems, 6304-TM-010) or vehicle (PBS) for 28 h, after which cells were fixed and stained to evaluate markers.

### Bulk RNA-sequencing of early postnatal microglia

Forebrains from early postnatal mice were pooled by genotype, and then microglia were isolated and plated in 12-well poly-d-lysine-coated plates (Corning, 354470) at a density of 600,000 cells per well, 2 wells per genotype. After 72 h, trypsin was used to remove cells prior to centrifugation for 5 min at 1230 x *g*, followed by resuspension in PBS, and re-centrifugation. Pellets were stored at −80 °C before further processing and sequencing by Genewiz (Azenta), with each sample representing independent isolations from pooled pups. RNA was extracted, and quality was measured using TapeStation Analysis Software 3.2 (Agilent), and concentration was determined using Qubit assay, with all samples exhibiting RNA Integrity Number (RIN) = 10. Sequencing libraries were prepared with poly(A) selection and sequenced using Illumina HiSeq (2 x 150 bp paired-end) according to manufacturer protocols. Reads were mapped to the Mus musculus GRCm38 genome. DESeq2 (version 1.46.0) was used for differential expression analysis, modeling batch as a covariate, and genes with adjusted *P* < 0.05 (using Benjamini-Hochberg procedure) were analyzed by Over-representation analysis of Gene Ontology (GO) terms^33^ (https://www.gsea-msigdb.org/gsea/index.jsp; MSigDB 2025.1; FDR < 0.05 for pathways). All genes, except those not mapping to known Entrez identifiers, were ranked by Wald statistic values for ranked GSEA using gseGO from the R clusterprofiler package^32^. Enrichment for microglial senescence genes was examined with the ranked list using default parameters for pre-ranked GSEA against established senescence gene sets filtered for myeloid/microglial genes (expressed in >10% myeloid/microglia from Tabula Muris^99^). Volcano plot was generated with the R package ggplot2, and plot customization was carried out using dplyr, patchwork, and ggrepel packages, and significant upregulated and downregulated DEGs (*P*_*adj*_ < 0.05) were color-coded according to direction of change. Weighted gene co-expression network analysis (WGCNA) was performed on all genes with a minimum of 10 counts, as previously described^100^, for signed networks, modeling batch as a covariate. Modules were generated to identify co-expressed and co-regulated genes according to genotype, and significant modules (*P* < 0.05) were used as input for over-representation analysis of GO terms.

### Immunoblotting

To perform western blot analysis of TIMP2 in primary microglia, cell pellets from pooled samples were lysed in RIPA buffer (Thermo Fisher) containing protease Inhibitor cocktail (Roche) for 30 min on ice, collecting lysates following a 30-min centrifugation at 15,000 x *g*. 80 μg of protein per sample was loaded on Bolt 4–12% Bis-Tris Plus Gels (Invitrogen). Conditioned media was spun at 1000 x *g* for 10 min to remove debris. Protease inhibitor cocktail was added to supernatant prior to concentration of media by centrifugation at 4000 x *g* for 40 min using Amicon Ultra-15 centrifugal filter unit (Amicon, UFC901024) before loading on 4–12% NuPAGE Bis-Tris denaturing gels (Invitrogen). Blots were probed with anti-TIMP2 (1:5000; D18B7, Cell Signaling) or anti-actin (1:10000; A5060; Sigma), and then developed as previously described^26^, with multiple blots per experiment developed in parallel where necessary. Band intensities were quantified using ImageJ software as described^42^.

### Immunocytochemistry

Following fixation with 4% paraformaldehyde for 20 min at room temperature, primary microglia were washed three times with PBS, incubated with blocking solution (10% donkey serum and 0.1% triton-X in PBS) for 1 h, and then incubated overnight at 4 °C with primary antibodies in blocking solution at the following concentrations: IBA1, 1:500, (019-19741, Wako); CD68, 1:200, (MCA1957, BioRad), TIMP2, 1:100 (AF971, R&D systems, [2-day incubation]), p16INK4a, 1:500, (MA5-17142, Invitrogen); p21, 1:500 (ab109199, Abcam); SA-β-Gal kit (SG07-10, Dojindo, used per manufacturer instructions). Cells were washed and incubated in corresponding donkey secondary antibodies at 1:200 for 1 h: anti-rabbit Alexa-Fluor 594 (A-21207, Invitrogen); anti-rat Alexa-Fluor 647 (ab150155, Abcam); anti-goat Alexa-Fluor 488 (A-11055, Invitrogen); anti-mouse DyLight 488 (SA510166, Invitrogen); anti-rabbit Alexa-Fluor 488 (A-21206, Invitrogen). Staining was visualized and imaged using the Keyence BZ-X700 microscope or Zeiss LSM780 upright confocal microscope using a 40x objective. 2–3 wells per mouse were plated, and 3–4 images per well were averaged for 3 technical replicates per mouse. The value for each mouse represents the mean of these replicates.

### Immunohistochemistry and imaging

Following injection with a ketamine (90 mg/kg) and xylazine (10 mg/kg) cocktail, mice were perfused transcardially with ice-cold 0.9% saline and approved secondary physical methods were used to ensure euthanasia prior to isolation of tissue for downstream endpoints. Brains were dissected and postfixed in 4% paraformaldehyde for 48 h and then preserved with 30% sucrose in PBS. 40-μm sections were prepared from hemibrains and sectioned using a freezing-sliding microtome and stored at −20 °C in cryoprotectant media prior to immunohistochemistry according to our previous protocol^16^. Briefly, free-floating sections were blocked in appropriate serum (10%) before incubation in primary antibody at 4 °C overnight. Primary antibodies were used at the following concentrations: IBA1, 1:500, (Wako, 019-19741); CD68, 1:200, (MCA1957, BioRad); VGLUT1, 1:2000, (135318, Synaptic Systems); CD68, 1:500, (137001, BioLegend, paired with IBA1 and VGLUT1 co-staining); TLR7, 1:1000 (NBP2, Novus Biologicals), TMSB4X, 1:500 (19850-1-AP; Proteintech). Sections were incubated with corresponding fluorescently-conjugated donkey secondary antibodies for 1 h (1:200 in TBST): anti-rabbit Alexa-Fluor 594 (A-21207, Invitrogen); anti-rat Alexa-Fluor 488 (A-21208, Invitrogen); anti-guinea pig Alexa-Fluor 647 (706605148, Jackson ImmunoResearch); anti-rabbit Alexa Fluor 555 (A-31572, Invitrogen); anti-rabbit Alexa-Fluor 647 (A-31573, Invitrogen); anti-goat Alexa-Fluor 488 (A-11055, Invitrogen); anti-goat Alexa-Fluor 647 (A-21447, Invitrogen); anti-rat Alexa-Fluor 647 (ab150155, Abcam), followed by DAPI, where indicated, for 15 min. Stained sections were mounted and coverslipped with ProLong Gold (Invitrogen).

For the various markers imaged in the dentate gyrus, 2 × 2 tile-scanning images were acquired through the entire z-plane of sections (1-μm intervals across ~40 μm) at 40x/1.4 oil DIC objective using either a Zeiss LSM780 or LSM900 upright confocal microscope. For striatum (caudate/putamen) or retrosplenial cortex, 1 × 1 images were acquired through the entire z-plane of sections at 40x/1.4 oil DIC objective using a Zeiss LSM780. For all imaging experiments, 3–4 sections per animal were imaged according to stereological principles, as previously described^16^. FIJI was used to perform thresholded “percentage area” or count analysis of cells expressing CD68, TLR7, TMSB4X and/or IBA1 across mouse experiments in a blinded fashion. The count data were normalized to the area of the analyzed ROI.

#### Imaris-based imaging and analysis

Following IHC for VGLUT1, IBA1, and CD68, images containing dentate gyrus were acquired using a 63X oil-immersion objective lens on a Zeiss LSM780 with 0.33-μm z-step size across ~20-μm z-stack (1024 × 1024 pixels). 3D reconstruction of microglia was performed using Imaris (Imaris 10.2, Oxford Instruments). Microglial surfaces were created using machine learning-based training on the IBA1 channel, which were then used to mask the CD68 channel. A surface was then created for the microglial-masked CD68 and used to mask the VGLUT1 signal. A surface for VGLUT1 signal within a microglial lysosome was created, together creating VGLUT1^+^ surfaces within a microglial lysosome. The total volume of VGLUT1 within lysosomes was normalized to the volume of the microglial surface (IBA1 channel) analyzed. Three sections per mouse were imaged, and the results for analyzed microglia per mouse were averaged to represent a value for each mouse for statistics.

#### Microglia morphology analysis

Morphology analyses were performed on microglia with completed surfaces rendered from images from 3 sections per mouse acquired on Zeiss LSM780 with 63X oil-immersion objective lens with 0.33-µm z-step size across ~20-µm z-stack (1024 × 1024 pixels). 3D reconstruction of microglia was performed using Imaris (Imaris 10.2, Oxford Instruments), creating microglial surfaces with machine learning-based training on the IBA1 channel to analyze microglia volume, surface area, and prolate or oblate ellipticity from individual microglia. Sholl analyses were performed on microglial processes traced by Imaris Filament Tracer. Filament Sholl Analysis XTension was applied to record the number of microglial processes intersecting with concentric circles (10-µm step-size), with soma selected as the seed point. Sholl data were analyzed in R Studio by a linear mixed-effect model using the number of intersections modeled as a function of experimental group and distance from soma (modeled by a quadratic polynomial). Random intercepts were included for batch, mouse, section, and cell, with hierarchical nesting (intersection ~ group * poly(radius, 2) + (1|batchID/mouseID/sectionID/cellID).

### Recombinant protein injections

For experiments in which mice were treated systemically with TIMP2, aged mice were given intraperitoneal (i.p.) injections (50 μg/kg) every other day with either vehicle (PBS) or recombinant mouse TIMP2 (R&D Systems, 6304-TM-010) for a total of 8 injections, using our established protocol, a method used previously to provide revitalization of hippocampal plasticity and cognitive function^16^.

### Nuclei Isolation and snRNA-seq

Full hippocampi were isolated, flash-frozen, and stored at −80 °C until processing. Hippocampi were thawed on ice, and nuclei were isolated using established protocols^62^. Briefly, each hippocampi was mechanically dissociated using a Dounce homogenizer (VWR) with 2 ml of EZ lysis buffer (Millipore Sigma). Samples were centrifuged, resuspended in fresh lysis buffer, and centrifuged again. Cell pellets were gently washed with 4 ml ice-cold PBS, and supernatant was removed prior to resuspending pellets in 100 μl of ice-cold PBS with 0.04% BSA (NEB) and 1 U/μL RNAse inhibitor (Millipore Sigma). Each sample was filtered through 40-μm FlowMi cell strainers (Millipore), and nuclei were counted (aided by trypan blue) to assess nuclei viability using an EVOS M7000. Two biological samples from the same treatment condition were pooled to load 20,000 nuclei total (10,000 nuclei each) for a total of *N* = 2 samples per treatment condition. Libraries were prepared and sequenced by the Single-cell & Spatial Technologies Team, Center for Advanced Genomics Technology Genomics Core (ISMMS) using the 10x Genomics Chromium platform for library preparation (V2 3’ GEX protocol) according to the manufacturer’s instructions. Briefly, cDNA was extracted from Gel-Bead in Emulsions (GEMs) obtained from the sample chip in the Chromium controller, and library quality control was performed with MiSeq Nano. Libraries were run on Illumina NovaSeq 6000 S4 Flowcell (2 × 100 nt paired-end read length) configuration, targeting 50,000 reads per cell.

Raw read data were analyzed by 10x CellRanger (v7.1.0) to align to the reference genome (Mus musculus, version mm10 2020-A). Downstream processing was performed in the R programming environment (v4.2.0 or v4.4.2). Ambient RNA was removed from samples using SoupX^101^ (v1.6.2) and manual application of a stringent contamination fraction on a per-sample basis. After initial filtering, doublets were identified and removed using DoubletFinder^102^ (v.2.04). Further filtering involved removing genes expressed in fewer than 3 cells, cells with a UMI count under 500, and cells with only 500 genes per cell to ensure we analyzed high-quality cells. The Seurat object was then log-normalized, and variable features were identified using the FindVariableFeatures function. ScaleData was then used to regress cells with more than 2% mitochondrial, ribosomal large subunit (RPL) and ribosomal small subunit (RPS) gene expression content. Using STACAS (v2.2.0), a semi-supervised data integration method, we integrated all samples^103^, which were used for cell clustering. Using Seurat (v5.2.1), integrated samples were scaled, and clusters were identified using FindNeighbors (using top 50 principal components) and FindClusters (Resolution = 0.25). To identify markers of each cluster, the RNA assay was processed using NormalizeData, FindVariableFeatures, ScaleData (regressing out > 2% mitochondria, RPS, and RPL), and FindMarkers. We determined cluster identity by assessing the top 30 markers on DropViz, further confirming identities with ScType^63^ and by examining literature-based expression of marker genes for each cell type. Clusters identified to be microglia were subclustered (using the top 15 principal components and a resolution of 0.5). Clusters suspected as containing doublets, i.e., marked by high nCount_RNA and nFeature_RNA, as well as a lack of microglial genes as cluster markers, were removed, and the remaining cells were re-clustered using the top 15 principal components and a resolution of 1. Pathways representing each subcluster were evaluated using the significantly upregulated marker genes in each subcluster (*P*_adj_ < 0.05; via FindAllMarkers) and by running these markers in Ingenuity Pathway Analysis (IPA, Qiagen) and gene ontology over-representation analysis using MSigDB^33^. To compare shifts in the proportion of subclustered cells between treatment conditions, we employed scProportionTest^104^ (v.0.0.0.9000).

Differential gene expression analysis for the microglia cluster was conducted using DESeq2, and IPA was run on significant genes (*P*_adj_ < 0.05). After removing genes not mapping to Entrez identifiers, we performed GSEA using a ranked list of all genes (ranked by signed fold-change*-log_10_*P*-value_adj_) tested in the differential expression analysis to assess enrichment in published gene sets representing characterized microglial states^76^. GSEA was performed in R using the clusterProfiler^32^ package (v4.14.6), implementing the Fast GSEA (fgsea) algorithm with default parameters^105^. The Benjamini-Hochberg correction method was applied to adjust for multiple comparisons for differential expression and pathway analyses.

To examine microglia genes that were co-regulated and co-expressed following TIMP2 treatment, WGCNA was performed using the R package hdWGCNA (v.0.4.08) for high-dimensional transcriptomic data^106^. Genes were extracted for WGCNA from microglia clusters following cell-type identification, and WGCNA was performed for signed networks for microglial genes. Significant modules (*P* < 0.05) were further analyzed for over-representation analyses using MSigDB. The R package CellChat (v.1.6.1) was utilized by accessing the mouse database with default settings for computing communication probability, filtering communication, and aggregating networks^107^. Alterations in cell interactions were assessed between microglia and neuronal clusters from the normalized expression matrix, where interactions between microglia as “sender” and neurons as “receiver” were calculated. Relative microglia-neuron information flow was calculated from summed communication probabilities, which were first trimean-averaged and adjusted for population size, for ligand-receptor pairs for each pathway. Individual ligand-receptor pair interaction probabilities were calculated from model-based estimates for interaction strength, with *P*-values derived from bootstrapping.

### 1-MDa in vivo microdialysis

Stereotaxic surgery and 1-MDa in vivo microdialysis were performed as previously described^26^. Mice were anesthetized with 2% isoflurane, and the head was shaved and fixed in a stereotaxic apparatus (Stoelting Co.). Skull position was leveled, and a hole was drilled at bregma −3.1 mm, 2.5 mm lateral to target the left caudal hippocampus. Another hole was made diagonal to the first to position an anchoring bone screw. After the meninges were removed, an AtomosLM Guide Cannula (PEG-12, Eicom) was inserted at a 12° angle, 1.2 mm below the brain surface at the target location. The cannula was secured, and the skin was closed using dental cement and surgical adhesive glue. An AtmosLM Dummy Cannula (PED-12, Eicom) was inserted into the guide cannula and secured as the animal recovered in a clean cage on a heating pad. Approximately 12 h following surgery, 1-MDa in vivo microdialysis probes (AtmosLM; Emicon) were inserted into the caudal hippocampus. To calibrate the system prior to probe insertion, inlet tubing (FEP tubing 0.65 mm OD x 0.12 mm ID, BASi) was connected to a syringe pump (KdScientific) that perfused artificial cerebrospinal fluid (aCSF) containing, in mM: 1.3 CaCl_2_, 1.2 MgSO_4_, 3 KCl, 0.4 KH_2_PO_4_, 25 NaHCO_3_, 122 NaCl, pH 7.35, at a rate of 1.2 μl/min. The outlet tubing was connected to a peristaltic pump (MAB 20, SciPro), which was carefully calibrated to obtain a pull rate between 0.9–1.1 μl/min. Probe integrity was tested, and once passing initial quality control, it was connected to the inlet and outlet ports. The push-pull system was connected to the probe and allowed to equilibrate prior to implanting in the mouse brain. The probe was secured with a cap-nut, and mice were placed in a Raturn (Stand-Alone Raturn System, BASi) and tethered by a loose collar to enable free movement while preventing tangling of microdialysis tubing. Dialysate from brain ISF was collected hourly at ~1.0 μl/min into a refrigerated fraction collector (MAB 85 Fraction Collector, SciPro) and frozen at −80 °C after collection for pooling and subsequent measurement. In microdialysis experiments involving LPS administration, mice were given a single i.p. injection of LPS (5 mg/kg in PBS), and samples were collected for an additional 10 h. For microdialysis experiments, mice were given *ad libitum* access to food and water and were kept under constant light conditions to minimize the impact of circadian protein flux, as previously described^108^.

### Protein analysis from ISF dialysates

Hippocampal ISF dialysate samples were thawed on ice and pooled for measurements. For WT and TIMP2 KO comparisons, samples representing the stable 24-h steady-state period were pooled within each mouse for subsequent measurements. For LPS experiments, the period representing a stable baseline over a 12-h period were pooled within each mouse to serve as the pre-LPS sample per mouse. Samples corresponding to hours six to ten following LPS treatment were pooled within each mouse to serve as the post-LPS treatment samples per mouse. For all experiments, 230 μl of pooled sample was concentrated approximately six-fold by centrifugation at 14,000 x *g* for 75 min at 4 °C with a concentrator unit (Amicon Ultra-0.5 Centrifugal Filter Units (MilliporeSigma). 25 μl of concentrated sample was loaded on a 96-well plate using the Olink Target Mouse Exploratory Panel, encompassing cytokines and chemokines (Olink, Uppsala, Sweden) in coordination with the Human Immune Monitoring Center (ISMMS). Data was represented as Normalized Protein Expression (NPX) unit on a log2 scale.

### Statistical analysis

Statistics were performed using GraphPad Prism version 10 (GraphPad Software) or R programming environment (version 4.4.2 or version 4.2.0) using tests (α = 0.05) described in figure legends with *P*-values indicated within figure panels. All relevant statistical tests were two-sided.

### Reporting summary

Further information on research design is available in the Nature Portfolio Reporting Summary linked to this article.

## Supplementary information

Supplementary Information

Reporting Summary

Transparent Peer Review file

## Source data

Source Data

## Data Availability

snRNAseq and bulk RNAseq data generated in this study has been deposited to the NCBI’s Gene Expression Omnibus database and are available through accession numbers GSE297916 and GSE297917, respectively. Source data are provided with this paper.
