# Supplementary material for: Youth-associated protein TIMP2 regulates microglial state and function in healthy and aged mice
Source: Nat Commun. 2026 Aug 12;17:8173. doi: 10.1038/s41467-026-74906-z (PMC13469711; doi:10.1038/s41467-026-74906-z)
Supplement: Supplementary file 1 [file 41467_2026_74906_MOESM1_ESM.pdf]

Youth-associated protein TIMP2 regulates microglial state and function in healthy and aged mice

Brittany M. Hemmer[1,2,3,4,5], Sarah M. Philippi[1,2,3,4,5], Ana Catarina Ferreira[1,2,3,4], Samuele F. Petridis[1,2,3,4], Annie Phan[1,2,3,4], Joseph M. Castellano[1,2,3,4]*

[1]Nash Family Department of Neuroscience, Friedman Brain Institute, Icahn School of Medicine at Mount Sinai, New York, NY, USA

[2]Ronald M. Loeb Center for Alzheimer's Disease, Icahn School of Medicine at Mount Sinai, New York, NY, USA

[3]Department of Neurology, Icahn School of Medicine at Mount Sinai, New York, NY, USA

[4]Black Family Stem Cell Institute, Icahn School of Medicine at Mount Sinai, New York, NY, USA

[5]Graduate School of Biomedical Sciences, Icahn School of Medicine at Mount Sinai, New York, NY, USA

*Correspondence:

Joseph M. Castellano, Ph.D.

Icahn School of Medicine at Mount Sinai

1425 Madison Avenue, Box 1065

Phone: 212-659-5913

E-mail: joseph.castellano@mssm.edu

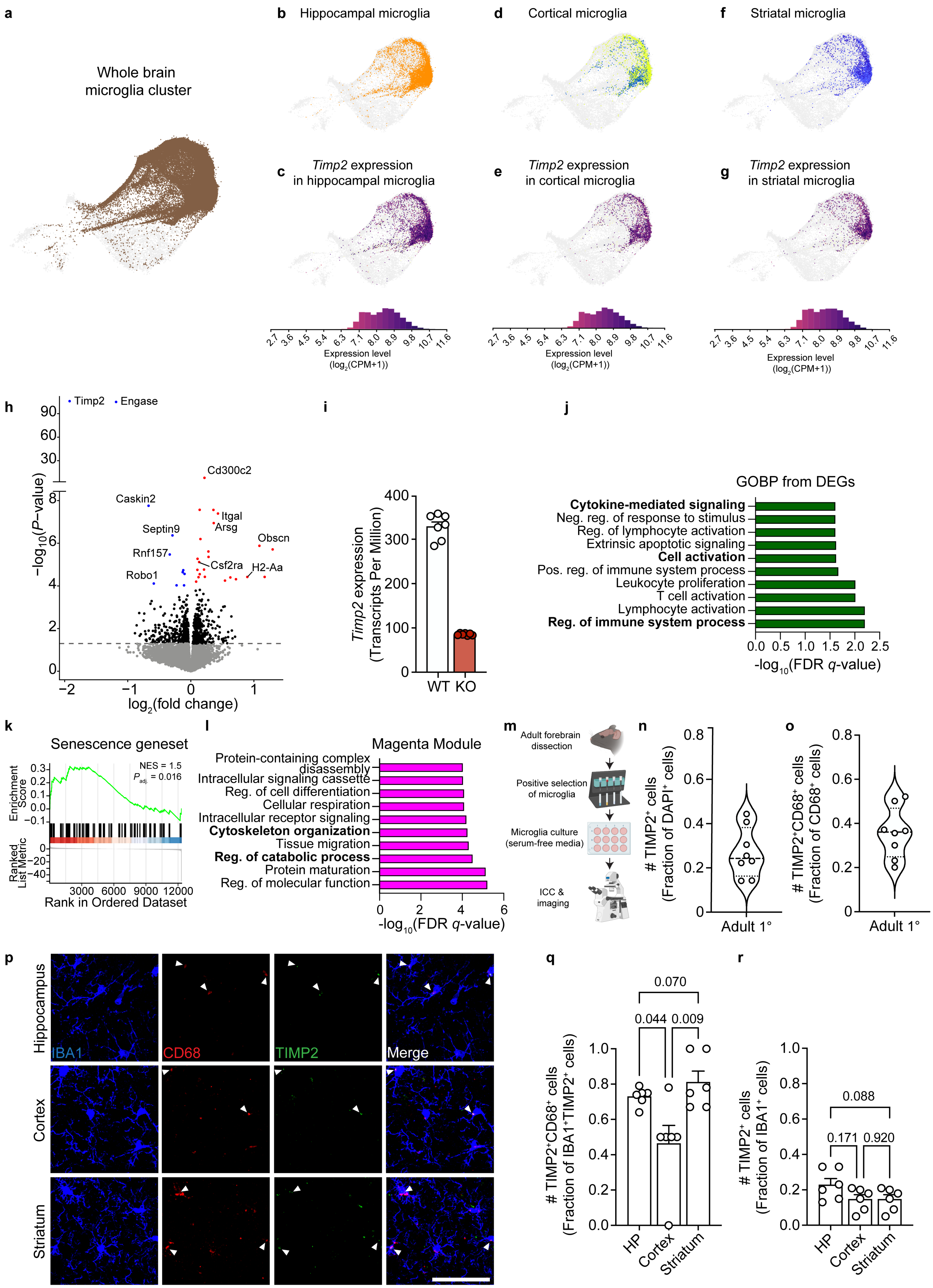

Whole brain microglia cluster
Hippocampal microglia
Cortical microglia
Striatal microglia
Timp2 expression in hippocampal microglia
Timp2 expression in cortical microglia
Timp2 expression in striatal microglia
Expression level (log2(CPM+1))
Timp2
Engase
Cd300c2
Caskin2
Itgal
Arsg
Septin9
Obscn
Rnf157
Csf2ra
H2-Aa
Robo1
−log10(P−value)
log2(fold change)
Timp2 expression (Transcripts Per Million)
WT
KO
GOBP from DEGs
Cytokine-mediated signaling
Neg. reg. of response to stimulus
Reg. of lymphocyte activation
Extrinsic apoptotic signaling
Cell activation
Pos. reg. of immune system process
Leukocyte proliferation
T cell activation
Lymphocyte activation
Reg. of immune system process
-log10(FDR q-value)
Senescence geneset
NES = 1.5
Padj = 0.016
Enrichment Score
Ranked List Metric
Rank in Ordered Dataset
Magenta Module
Protein-containing complex disassembly
Intracellular signaling cassette
Reg. of cell differentiation
Cellular respiration
Intracellular receptor signaling
Cytoskeleton organization
Tissue migration
Reg. of catabolic process
Protein maturation
Reg. of molecular function
Adult forebrain dissection
Positive selection of microglia
Microglia culture (serum-free media)
ICC & imaging
TIMP2+ cells (Fraction of DAPI+ cells)
Adult 1°
TIMP2+CD68+ cells (Fraction of CD68+ cells)
Hippocampus
Cortex
Striatum
IBA1
CD68
TIMP2
Merge
TIMP2+CD68+ cells (Fraction of IBA1+TIMP2+ cells)
0.070
0.044
0.009
HP
TIMP2+ cells (Fraction of IBA1+ cells)
0.088
0.171
0.920

Supplementary Figure 1. TIMP2 is expressed in microglia across regions and in cells with CD68 expression.

(a) Data from Allen Brain Cell scRNAseq and MERFISH Atlas from whole brain microglia (brown), with subsets corresponding to **(b)** hippocampal microglia (orange), and **(c)** *Timp2* expression within hippocampal microglia. Additional microglia subsets including **(d)** cortical microglia (green = retrosplenial cortex; blue = visual cortex), with **(e)** corresponding *Timp2* expression, and **(f)** striatal microglia (dark blue = ventral striatum; light blue = dorsal striatum), with **(g)** *Timp2* expression within striatum. **(h)** Volcano plot of bulk RNAseq DEGs from early postnatal TIMP2 KO vs. WT primary microglia (N = 7 samples per genotype, with each sample representing a pool of 5 pups (P6-P7); sex-matched; mean ± SEM), showing $\log_2$ fold-change versus $-\log_{10} P_{adj}$-value for each gene, highlighting significant DEGs ($P_{adj} < 0.05$) in red (upregulated) or blue (downregulated). **(i)** Gene expression (transcripts per million) values for *Timp2* from bulk RNAseq experiments in "**h**". **(j)** Significant GOBP pathways ($P_{adj} < 0.05$, FDR) and NES from over-representation analysis of significant ($P_{adj} < 0.05$) DEGs from "**h"** (calculated from DESeq2, two-sided Wald test, Benjamini-Hochberg-adjusted)**.** **(k)** GSEA enrichment plot for senescence gene set from ranked genes (TIMP2 KO vs WT, using two-tailed Wald statistic-ranked DEGs) with green lines representing running enrichment score, black vertical bars indicating position of genes in ranked list (upregulated in red and downregulated in blue). **(l)** Significant GOBP terms (FDR *q*-value < 0.05) from over-representation analysis on genes from significant "Magenta" module from Fig. 1e. **(m)** Schematic diagram of adult microglia isolation, culturing, and imaging, with (**n**) quantification of the number of $TIMP2^+$ cells as a fraction of $DAPI^+$ cells in WT primary microglia cultures stained by ICC, or **(o)** the number of $TIMP2^+CD68^+$ cells as a fraction of CD68+ cells (N = 8 mice, 5-6 month-old mice; sex-matched; 2 independent experiments combined; scale bar = 100 µm; violin plots of median with quartiles. **(p)** Representative confocal images of hippocampus, cortex, and striatum from 6-7-month-old mice stained with anti-IBA1, anti-CD68, and anti-TIMP2 antibodies (scale bar = 50 µm; arrowheads indicate $IBA1^+CD68^+TIMP2^+$ cells), with (**q**)

quantification of number of TIMP2$^+$CD68$^+$ cells as fraction of IBA1$^+$TIMP2$^+$ cells for each region (N = 6 mice per group) and **(r)** number of TIMP2+ cells as fraction of IBA1$^+$ cells across brain regions; 1-way ANOVA, Tukey's post test. Source data are provided as Source Data file. Created in BioRender: Philippi, S. (2026) https://BioRender.com/2bm4j2d.

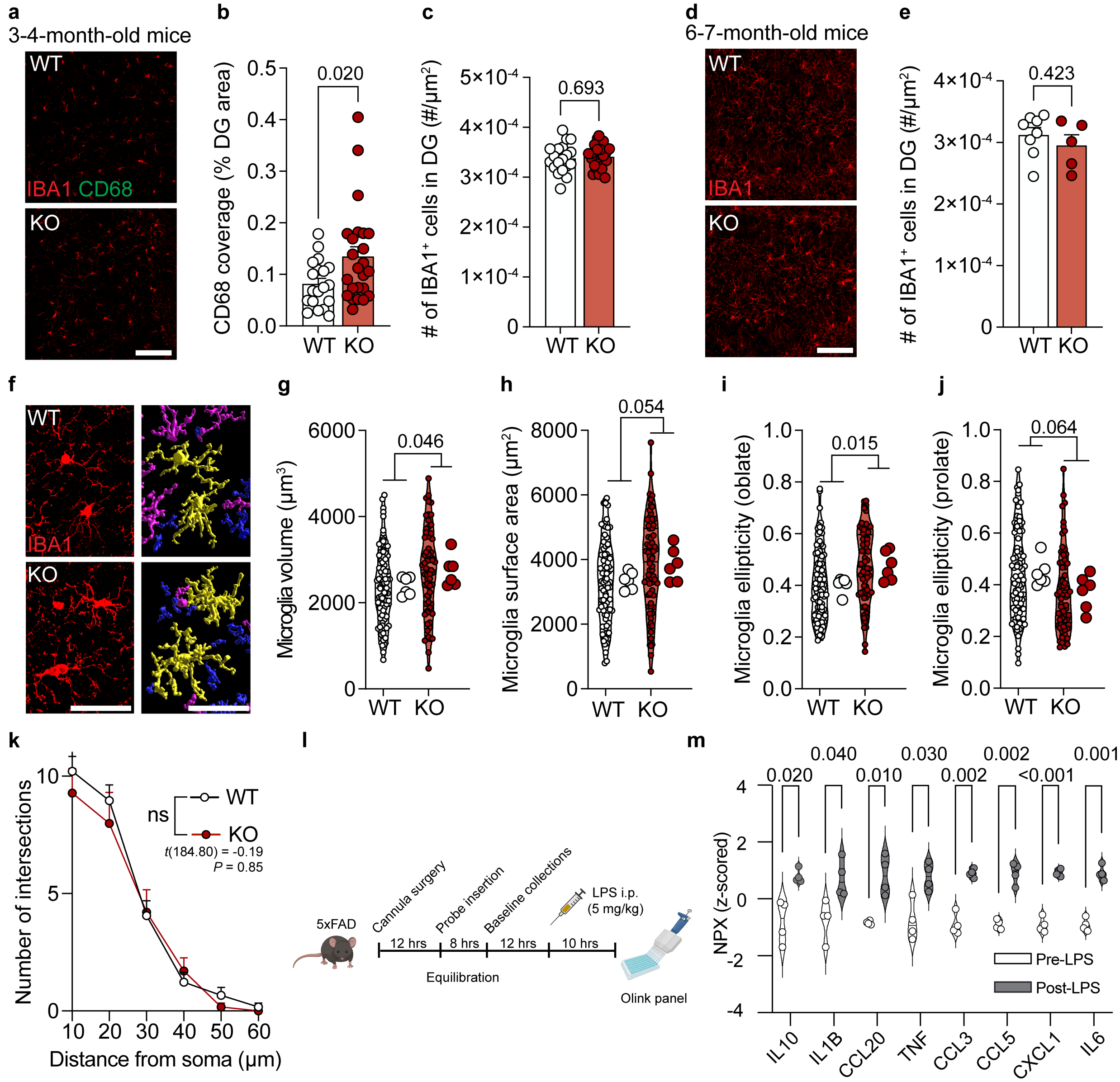
a
3-4-month-old mice
WT
IBA1 CD68
KO
b
CD68 coverage (% DG area)
0.020
WT KO
c
# of IBA1+ cells in DG (#/µm2)
0.693
WT KO
d
6-7-month-old mice
WT
IBA1
KO
e
# of IBA1+ cells in DG (#/µm2)
0.423
WT KO
f
WT
IBA1
KO
g
Microglia volume (µm3)
0.046
WT KO
h
Microglia surface area (µm2)
0.054
WT KO
i
Microglia ellipticity (oblate)
0.015
WT KO
j
Microglia ellipticity (prolate)
0.064
WT KO
k
Number of intersections
Distance from soma (µm)
ns
WT
KO
t(184.80) = -0.19
P = 0.85
l
5xFAD
Cannula surgery
Probe insertion
Baseline collections
LPS i.p.
(5 mg/kg)
12 hrs
8 hrs
12 hrs
10 hrs
Equilibration
Olink panel
m
NPX (z-scored)
0.020
0.040
0.010
0.030
0.002
0.002
<0.001
0.001
Pre-LPS
Post-LPS
IL10
IL1B
CCL20
TNF
CCL3
CCL5
CXCL1
IL6

Supplementary Figure 2. TIMP2 KO mice exhibit altered state and morphology associated with activation.

(a) Representative confocal images of DG from 3-4-month-old WT and TIMP2 KO mice stained with anti-CD68 and anti-IBA1 antibodies (scale bar = 100 μm) with **(b)** quantification of thresholded DG area covered by CD68$^+$ staining and **(c)** the number of IBA1$^+$ cells normalized to DG area (N = 19 WT (9 female, 10 male), 24 KO (13 female, 11 male). **(d)** Representative confocal images of DG from 6-7-month-old WT and TIMP2 KO mice stained with anti-IBA1 antibody (scale bar = 100 μm) with **(e)** quantification of the number of IBA1$^+$ cells normalized to DG area (N = 8 WT, 5 KO female mice; mean ± SEM). **(f)** Representative confocal staining (left; scale bar = 15 μm) of DG from 6-7-month-old TIMP2 KO and WT mice stained with anti-IBA1 antibody, with Imaris-based reconstruction (right; scale bar = 15 μm) of microglial surfaces, with corresponding quantification from TIMP2 KO and WT microglia of **(g)** volume, **(h)** surface area, **(i)** oblate and (**j**) prolate ellipticity, and **(k)** number of Sholl intersections over distance from soma (N = 6 mice per group; sex-matched; two-tailed nested *t*-test using cell (left replicates) and mouse (right replicates) as levels; linear mixed-effects model for Sholl analysis in (k). **(l)** Timeline of LPS treatment paradigm in mice undergoing high molecular-weight cut-off (HMWCO, 1-MDa) *in vivo* microdialysis (N = 4; 6-7-month-old male 5xFAD mice) with **(m)** brain ISF protein measurements, represented as z-scored NPX values for significant proteins (paired two-tailed *t*-test; median with quartiles). Two-tailed Student's *t*-test used in (b-c), (e), with Welch's correction applied in (b). Source data are provided as Source Data file. Created in BioRender: Philippi, S. (2026) https://BioRender.com/ruyfrku.

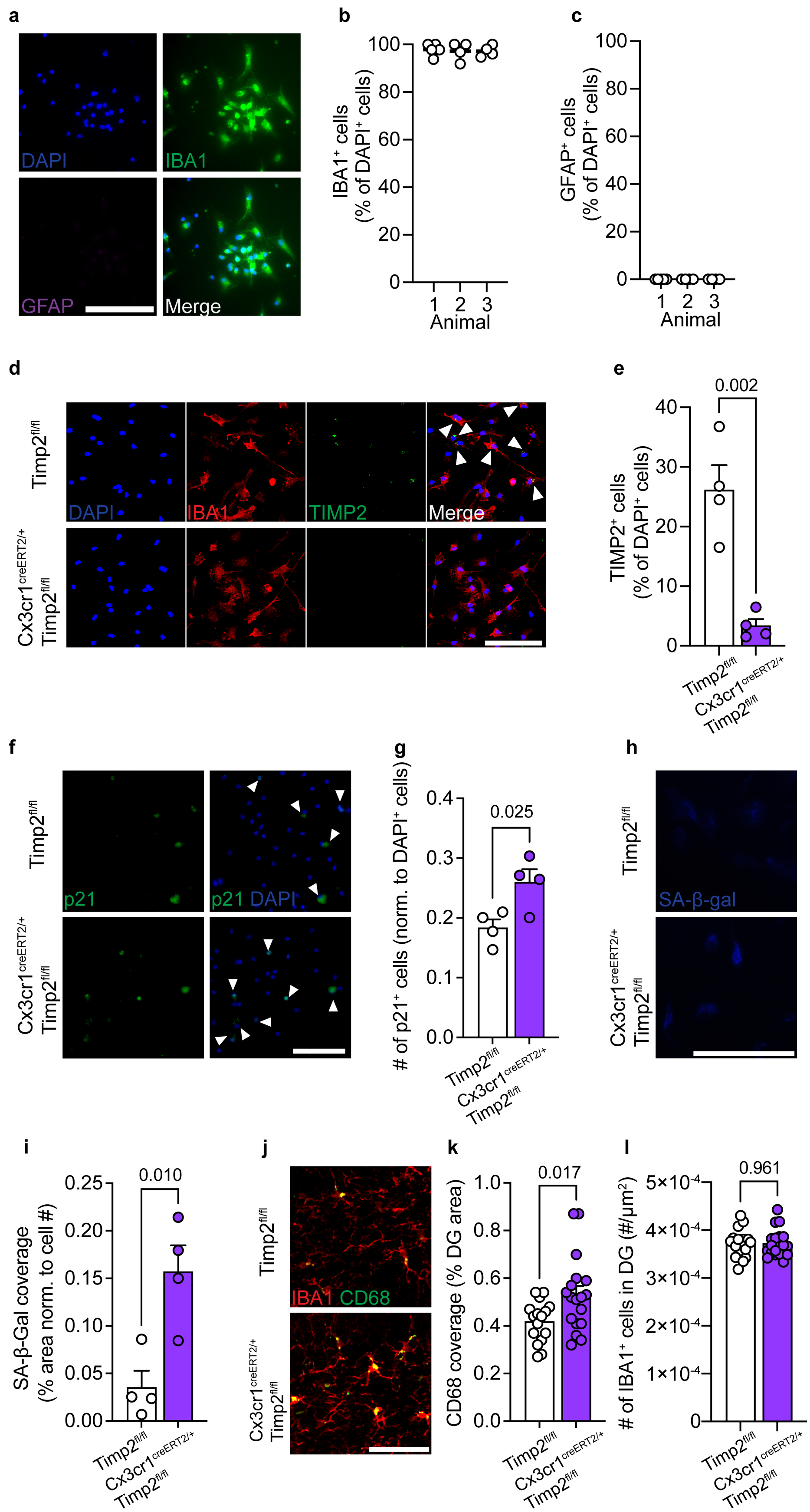
a
DAPI
IBA1
GFAP
Merge
b
IBA1+ cells (% of DAPI+ cells)
Animal
c
GFAP+ cells (% of DAPI+ cells)
Animal
d
Timp2fl/fl
Cx3cr1creERT2/+ Timp2fl/fl
DAPI
IBA1
TIMP2
Merge
e
0.002
TIMP2+ cells (% of DAPI+ cells)
Timp2fl/fl
Cx3cr1creERT2/+ Timp2fl/fl
f
p21
p21 DAPI
g
0.025
# of p21+ cells (norm. to DAPI+ cells)
h
SA-β-gal
i
0.010
SA-β-Gal coverage (% area norm. to cell #)
j
IBA1 CD68
k
0.017
CD68 coverage (% DG area)
l
0.961
# of IBA1+ cells in DG (#/μm2)

Supplementary Figure 3. Purity of primary microglia cultures and efficiency of microglia *Timp2* deletion

(a) Representative microscopy images of primary microglia cultures from WT mice stained with DAPI, anti-IBA1, and anti-GFAP antibodies (scale bar = 100 μm), with **(b)** quantification of IBA1$^+$ cells as percentage of DAPI$^+$ cells or **(c)** quantification of GFAP$^+$ cells as percentage of DAPI$^+$ cells (N = 5 replicates for mouse 1, 4 replicates each for mice 2 and 3; 9-month-old). **(d)** Representative images from primary microglia cultures from 5-month-old Timp2$^{fl/fl}$ control and Cx3cr1$^{CreERT2/+}$;Timp2$^{fl/fl}$ littermates stained by ICC with DAPI, anti-IBA1 antibody, and anti-TIMP2 antibody (arrowheads indicate TIMP2$^+$ microglia; N = 4 mice per group; sex-matched; scale bar = 100 μm), with **(e)** quantification of TIMP2$^+$ cells as percentage of DAPI$^+$ cell number (mean ± SEM). **(f)** Representative images from primary microglia cultures isolated from 10-month-old TIMP2$^{fl/fl}$ control and Cx3cr1$^{CreERT2/+}$;Timp2$^{fl/fl}$ littermates stained by ICC with DAPI and anti-p21 antibody (arrowheads indicate p21$^+$ cells; scale bar = 100 μm;), with **(g)** quantification of number of p21$^+$ cells normalized to total number of cells (DAPI$^+$) (N = 4 mice per group). **(h)** Representative images from primary microglia cultures isolated from 10-month-old TIMP2$^{fl/fl}$ control and Cx3cr1$^{CreERT2/+}$;Timp2$^{fl/fl}$ littermates stained with SA-β-gal (scale bar = 100 μm), with **(i)** SA-β-gal staining calculated as percentage of thresholded area normalized to cell number (N = 4 mice per group). **(j)** Representative confocal images of DG from 6-7-month-old TIMP2$^{fl/fl}$ control and Cx3cr1$^{CreERT2/+}$;Timp2$^{fl/fl}$ littermates stained with anti-IBA1 and anti-CD68 antibodies with **(k)** quantification of DG area covered by CD68$^+$ staining (N= 16 TIMP2$^{fl/fl}$ control, N=18 Cx3cr1$^{CreERT2/+}$;Timp2$^{fl/fl}$) and **(l)** quantification of the number of IBA1$^+$ cells per DG area (N= 17 TIMP2$^{fl/fl}$ control, N=19 Cx3cr1$^{CreERT2/+}$;Timp2$^{fl/fl}$) sex-matched mice; mean ± SEM; scale bar = 50 μm. Two-tailed Student's *t*-test. Source data are provided as Source Data file.

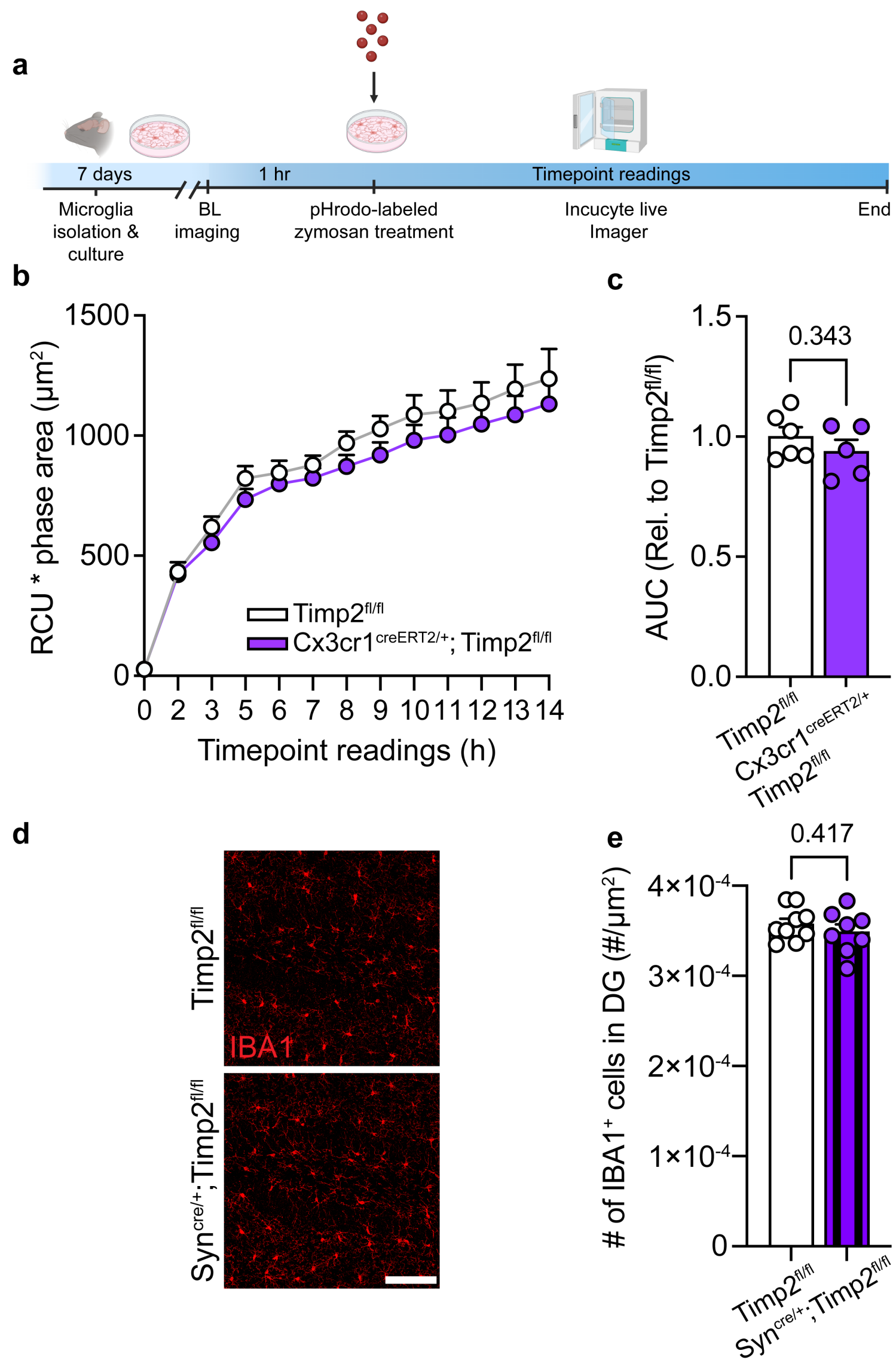
a
7 days
1 hr
Timepoint readings
Microglia isolation & culture
BL imaging
pHrodo-labeled zymosan treatment
Incucyte live Imager
End
b
RCU * phase area (μm²)
Timepoint readings (h)
Timp2fl/fl
Cx3cr1creERT2/+; Timp2fl/fl
c
0.343
AUC (Rel. to Timp2fl/fl)
Timp2fl/fl
Cx3cr1creERT2/+
Timp2fl/fl
d
Timp2fl/fl
IBA1
Syncre/+;Timp2fl/fl
e
0.417
# of IBA1+ cells in DG (#/μm²)
Timp2fl/fl
Syncre/+;Timp2fl/fl

Supplementary Figure 4. Microglial TIMP2 deletion does not affect zymosan uptake, and neuronal deletion of TIMP2 does not alter microglia number in the dentate gyrus

(a) Schematic diagram of treatment of adult primary microglia with pHrodo-labeled zymosan and subsequent Incucyte imaging to assess uptake across timepoint readings. **(b)** Timepoint readings with total integrated density (red object mean intensity [RCU, red, *x* average phage object area (cell, μm^2)]) of pHrodo-labeled zymosan signal in primary microglia cultured from tamoxifen-treated 4-5-month-old Cx3cr1$^{CreERT2/+}$;Timp2$^{fl/fl}$ and Timp2$^{fl/fl}$ control mice, with **(c)** corresponding uptake quantified using area under the curve (AUC; N = 6 Timp2$^{fl/fl}$, N = 5 Cx3cr1$^{CreERT2/+}$;Timp2$^{fl/fl}$ sex-matched mice; 2 independent experiments combined; mean ± SEM). **(d)** Representative confocal images of DG from 2-3-month-old Timp2$^{fl/fl}$ and Syn$^{Cre/+}$;Timp2$^{fl/fl}$ littermates stained with anti-IBA1 antibody with corresponding **(e)** quantification of the number of IBA1$^+$ cells normalized to DG area (N = 9 Timp2$^{fl/fl}$, N= 8 Syn$^{Cre/+}$;Timp2$^{fl/fl}$ female mice; scale bar = 100 μm; mean ± SEM). Two-tailed Student's *t*-test. Source data are provided as Source Data file. Created in BioRender: Philippi, S. (2026) https://BioRender.com/fblpd8f.

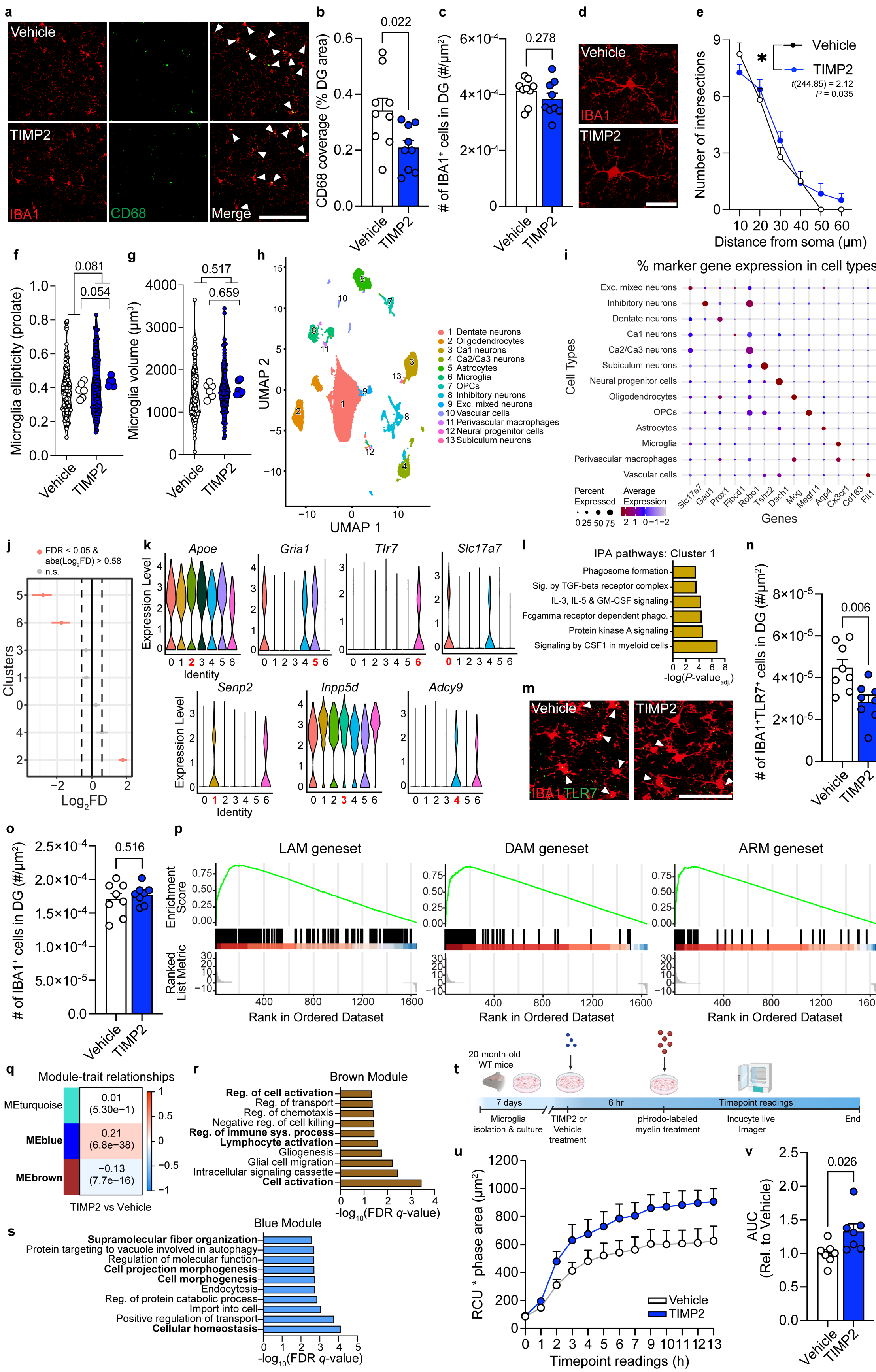
a
Vehicle
TIMP2
IBA1
CD68
Merge
b
0.022
CD68 coverage (% DG area)
Vehicle
TIMP2
c
0.278
# of IBA1⁺ cells in DG (#/µm²)
d
Vehicle
TIMP2
IBA1
e
Vehicle
TIMP2
t(244.85) = 2.12
P = 0.035
Number of intersections
Distance from soma (µm)
f
0.081
0.054
Microglia ellipticity (prolate)
g
0.517
0.659
Microglia volume (µm³)
h
UMAP 1
UMAP 2
1 Dentate neurons
2 Oligodendrocytes
3 Ca1 neurons
4 Ca2/Ca3 neurons
5 Astrocytes
6 Microglia
7 OPCs
8 Inhibitory neurons
9 Exc. mixed neurons
10 Vascular cells
11 Perivascular macrophages
12 Neural progenitor cells
13 Subiculum neurons
i
% marker gene expression in cell types
Exc. mixed neurons
Inhibitory neurons
Dentate neurons
Ca1 neurons
Ca2/Ca3 neurons
Subiculum neurons
Neural progenitor cells
Oligodendrocytes
OPCs
Astrocytes
Microglia
Perivascular macrophages
Vascular cells
Cell Types
Percent Expressed
Average Expression
Slc17a7
Gad1
Prox1
Fibcd1
Robo1
Tshz2
Dach1
Mog
Megf11
Aqp4
Cx3cr1
Cd163
Flt1
Genes
j
FDR < 0.05 & abs(Log2FD) > 0.58
n.s.
Clusters
Log2FD
k
Apoe
Gria1
Tlr7
Slc17a7
Senp2
Inpp5d
Adcy9
Expression Level
Identity
l
IPA pathways: Cluster 1
Phagosome formation
Sig. by TGF-beta receptor complex
IL-3, IL-5 & GM-CSF signaling
Fcgamma receptor dependent phago.
Protein kinase A signaling
Signaling by CSF1 in myeloid cells
-log(P-value adj.)
m
Vehicle
TIMP2
IBA1 TLR7
n
0.006
# of IBA1⁺TLR7⁺ cells in DG (#/µm²)
o
0.516
# of IBA1⁺ cells in DG (#/µm²)
p
LAM geneset
DAM geneset
ARM geneset
Enrichment Score
Ranked List Metric
Rank in Ordered Dataset
q
Module-trait relationships
MEturquoise
0.01 (5.30e−1)
MEblue
0.21 (6.8e−38)
MEbrown
−0.13 (7.7e−16)
TIMP2 vs Vehicle
r
Brown Module
Reg. of cell activation
Reg. of transport
Reg. of chemotaxis
Negative reg. of cell killing
Reg. of immune sys. process
Lymphocyte activation
Gliogenesis
Glial cell migration
Intracellular signaling cassette
Cell activation
-log10(FDR q-value)
s
Blue Module
Supramolecular fiber organization
Protein targeting to vacuole involved in autophagy
Regulation of molecular function
Cell projection morphogenesis
Cell morphogenesis
Endocytosis
Reg. of protein catabolic process
Import into cell
Positive regulation of transport
Cellular homeostasis
-log10(FDR q-value)
t
20-month-old WT mice
7 days
6 hr
Timepoint readings
Microglia isolation & culture
TIMP2 or Vehicle treatment
pHrodo-labeled myelin treatment
Incucyte live Imager
End
u
RCU * phase area (µm²)
Timepoint readings (h)
Vehicle
TIMP2
v
0.026
AUC (Rel. to Vehicle)
Vehicle
TIMP2

Supplementary Figure 5. Systemic TIMP2 treatment in aged mice alters microglial state and shifts microglial subclusters.

(a) Representative confocal images of DG from 20-month-old WT mice treated systemically with TIMP2 or vehicle, stained with anti-IBA1 and anti-CD68 antibodies (scale bar = 100 µm; arrowheads indicate $IBA1^{+}CD68^{+}$ cells), with corresponding **(b)** quantification of the percentage of DG area covered by $CD68^{+}$ staining and **(c)** quantification of the number of $IBA1^{+}$ cells normalized to DG area (N = 9 male mice per group; mean ± SEM). **(d)** Representative high-magnification confocal images anti-IBA1 antibody-stained microglia from DG of 20-month-old mice treated with vehicle or TIMP2 (scale bar = 20 µm) for Imaris-based morphology analyses, with **(e)** quantification of number of Sholl intersections over distance (µm) from soma, **(f)** microglia ellipticity (prolate) and **(g)** volume (N = 6 male mice per group; *P*-values reflect two-tailed nested *t*-tests, using cell and mouse replicates as levels, as well as standard mouse-level replicate comparison by two-tailed Student's *t*-test; linear mixed-effects model for Sholl analysis in (e)). **(h)** Uniform Manifold Approximation and Projection (UMAP) plot of hippocampal nuclei isolated from 20-month-old WT mice treated systemically with vehicle or TIMP2 (N = 4 male mice per group combined as 2 samples per group). **(i)** Dot plot of scaled expression of selected marker genes across annotated cell types. Dot size represents percentage of cells expressing the gene within each cell type, while color indicates the scaled average expression level of the gene across cells in the respective cluster. **(j)** Differences in cell proportions for each cluster between treatment conditions. Significant clusters are denoted in salmon (FDR < 0.05, Log_2 fold-enrichment > 0.58, relative to vehicle-treated mice). **(k)** Violin plots of marker genes across microglial subclusters from Fig. 5h. **(l)** Significant canonical IPA pathways (Benjamini-Hochberg-adjusted) for marker genes significantly upregulated in subcluster 1 (from one-tailed Wilcoxon Rank Sum P_{adj}-values (Bonferroni-adjusted)). **(m)** Representative confocal images of DG from 20-month-old WT mice treated systemically with TIMP2 or vehicle, stained with anti-IBA1 and anti-TLR7 antibodies (scale bar = 50 µm; arrowheads indicate $IBA1^{+}TLR7^{+}$ cells), with corresponding **(n)** quantification of

$IBA1^{+}TLR7^{+}$ cells normalized to DG area and **(o)** number of $IBA1^{+}$ cells normalized to DG area (N = 8 mice per group; males; mean ± SEM). **(p)** GSEA enrichment plots for LAM, DAM, and ARM gene sets based on the ranked list of microglial genes (TIMP2 vs. vehicle). Green lines represent running enrichment score, with vertical black bars indicating the position of genes from indicated gene sets in the ranked list and gene ranking direction indicated by color bar (red = upregulated, blue = downregulated). **(q)** Module-trait relationships plot based on high-dimensional (hd)WGCNA from snRNAseq dataset (TIMP2 vs. vehicle), with colored module assignments and corresponding membership values and module significance (P < 0.05, two-tailed Student's asymptotic *t*-test) in parentheses, with **(r)** significant GOBP terms (FDR <0.05) from over-representation analysis on significant "Brown" and (s) "Blue" modules on extracted genes. **(t)** Schematic diagram of treatment of adult primary microglia with pHrodo-labeled myelin and subsequent Incucyte imaging to assess uptake. **(u)** Timepoint readings, with total integrated density (red object mean intensity [RCU, red, *x* average phase object area (cell, μm^2)]) of pHrodo-labeled myelin signal in primary microglia cultured and treated with TIMP2 or vehicle from 20-month-old WT mice, with corresponding **(v)** uptake quantification using area under the curve (AUC; N = 7 mice per group; 2 independent experiments combined; sex-matched; mean ± SEM); Two-tailed Students *t*-test in (b-c), (n-p), (v). Source data are provided as Source Data file. Created in BioRender: Philippi, S. (2026) https://BioRender.com/13xrppj.
