# Supplementary material for: Youth-associated protein TIMP2 regulates microglial state and function in healthy and aged mice
Source: Nat Commun. 2026 Aug 12;17:8173. doi: 10.1038/s41467-026-74906-z (PMC13469711; doi:10.1038/s41467-026-74906-z)
Supplement: Supplementary file 2 [file 41467_2026_74906_MOESM2_ESM.pdf]

nature portfolio

Corresponding author(s): Joseph M. Castellano

Last updated by author(s): May 20, 2026

Reporting Summary

Nature Portfolio wishes to improve the reproducibility of the work that we publish. This form provides structure for consistency and transparency in reporting. For further information on Nature Portfolio policies, see our Editorial Policies and the Editorial Policy Checklist.

Statistics

For all statistical analyses, confirm that the following items are present in the figure legend, table legend, main text, or Methods section.

n/a	Confirmed	
☐	☒	The exact sample size (n) for each experimental group/condition, given as a discrete number and unit of measurement
☐	☒	A statement on whether measurements were taken from distinct samples or whether the same sample was measured repeatedly
☐	☒	The statistical test(s) used AND whether they are one- or two-sided<br>*Only common tests should be described solely by name; describe more complex techniques in the Methods section.*
☒	☐	A description of all covariates tested
☐	☒	A description of any assumptions or corrections, such as tests of normality and adjustment for multiple comparisons
☐	☒	A full description of the statistical parameters including central tendency (e.g. means) or other basic estimates (e.g. regression coefficient) AND variation (e.g. standard deviation) or associated estimates of uncertainty (e.g. confidence intervals)
☐	☒	For null hypothesis testing, the test statistic (e.g. F, t, r) with confidence intervals, effect sizes, degrees of freedom and P value noted<br>*Give P values as exact values whenever suitable.*
☒	☐	For Bayesian analysis, information on the choice of priors and Markov chain Monte Carlo settings
☒	☐	For hierarchical and complex designs, identification of the appropriate level for tests and full reporting of outcomes
☒	☐	Estimates of effect sizes (e.g. Cohen's d, Pearson's r), indicating how they were calculated

Our web collection on statistics for biologists contains articles on many of the points above.

Software and code

Policy information about availability of computer code

Data collection	Immunoblotting: Image lab 4.0.1 (BioRad)<br>Keyence imaging: BZ-X800 viewer (Keyence Corp)<br>Confocal imaging: Zen Black 2012 (Zeiss)
Data analysis	FIJI (version 2.14.0)<br>R (version 4.4.2 or 4.2.0)<br>WGCNA (version 1.74)<br>Prism (version 10.0.2 or 11.0)<br>Imaris (version 10.2, Oxford instruments)<br>Incucyte (version 2023 Rev2)<br>CellRanger (7.1.0)<br>STAR aligner (version 2.5.2b)<br>Seurat (version 5.2.1)<br>Doubletfinder (version 2.04)<br>SoupX (version 1.6.2)<br>STACAS (version 2.2.0)<br>scProportionTest (version 0.0.0.9000)<br>Ingenuity Pathway analysis Qiagen Inc<br>Gene ontology over-representation analysis with MSigDB

DESeq2 (version 1.38.3 or version 1.46.0, Bioconductor)
Pre-ranked GSEA using clusterProfiler (v4.14.6)
hdWGCNA (v.0.4.08)
CellChat (v.1.6.1)

For manuscripts utilizing custom algorithms or software that are central to the research but not yet described in published literature, software must be made available to editors and reviewers. We strongly encourage code deposition in a community repository (e.g. GitHub). See the Nature Portfolio guidelines for submitting code & software for further information.

Data

Policy information about availability of data

All manuscripts must include a data availability statement. This statement should provide the following information, where applicable:

- Accession codes, unique identifiers, or web links for publicly available datasets
- A description of any restrictions on data availability
- For clinical datasets or third party data, please ensure that the statement adheres to our policy

snRNAseq and bulk RNAseq data generated in this study were deposited to the NCBI's Gene Expression Omnibus database under accession number GSE297916 and GSE297917, respectively.

Research involving human participants, their data, or biological material

Policy information about studies with human participants or human data. See also policy information about sex, gender (identity/presentation), and sexual orientation and race, ethnicity and racism.

Reporting on sex and gender	This study does not include human participants.
Reporting on race, ethnicity, or other socially relevant groupings	This study does not include human participants.
Population characteristics	This study does not include human participants.
Recruitment	This study does not include human participants.
Ethics oversight	This study does not include human participants.

Note that full information on the approval of the study protocol must also be provided in the manuscript.

Field-specific reporting

Please select the one below that is the best fit for your research. If you are not sure, read the appropriate sections before making your selection.

☒ Life sciences ☐ Behavioural & social sciences ☐ Ecological, evolutionary & environmental sciences

For a reference copy of the document with all sections, see nature.com/documents/nr-reporting-summary-flat.pdf

Life sciences study design

All studies must disclose on these points even when the disclosure is negative.

Sample size	Sample sizes were based on pilot or similar previous experiments and based on 20% effect size between groups, α = 0.05, and power of 0.8.
Data exclusions	As mentioned in the methods, animals for cell culture experiments were excluded from analysis if cultures did not meet viability or quality control criteria (e.g. excessive debris). For CD68 coverage analysis in supplemental figure 3k, one animal from each group was removed based on statistical outlier identification using Grubbs test in Prism (version 10.0.2), which was due to unusual high background that affected thresholded area in sections from these animals for the anti-CD68 % area stain.
Replication	Cell culture experiments were repeated as mentioned in the figure legends. All attempts at replication were successful.
Randomization	Animals for in vivo and in vitro experiments were randomly assigned to treatment groups.
Blinding	Investigators were blinded to groups for indicated treatments, group allocation, processing samples, and data analysis.

Reporting for specific materials, systems and methods

We require information from authors about some types of materials, experimental systems and methods used in many studies. Here, indicate whether each material, system or method listed is relevant to your study. If you are not sure if a list item applies to your research, read the appropriate section before selecting a response.

Materials & experimental systems

n/a	Involved in the study
☐	☒ Antibodies
☒	☐ Eukaryotic cell lines
☒	☐ Palaeontology and archaeology
☐	☒ Animals and other organisms
☒	☐ Clinical data
☒	☐ Dual use research of concern
☒	☐ Plants

Methods

n/a	Involved in the study
☒	☐ ChIP-seq
☒	☐ Flow cytometry
☒	☐ MRI-based neuroimaging

Antibodies

Antibodies used

IBA1, (019-19741, Wako; 1:500); CD68, (MCA1957, BioRad; 1:500), TIMP2, (AF971, R&D systems; 1:100), p16INK4a, (MA5-17142, Invitrogen; 1:500), VGLUT1, (135318, Synaptic Systems; 1:2k); CD68, (137001, BioLegend, paired with IBA1 and VGLUT1 co-staining, 1:500); anti-p21, (ab109199, Abcam; 1:500); SA-β-Gal kit, (SG07-10, Dojindo); TLR7, (NBP2, Novus Biologicals; 1:1k); TMSB4X, (19850-1-AP; Proteintech; 1:500); Secondaries at 1:200, including anti-rabbit Alexa-Fluor 594 (A-21207, Invitrogen); anti-rat Alexa-Fluor 647 (ab150155, Abcam); anti-goat Alexa-Fluor 488 (A-11055, Invitrogen); anti-mouse DyLight 488 (SA510166, Invitrogen); anti-rabbit Alexa-Fluor 488 (A-21206, Invitrogen) for ICC and anti-rabbit Alexa-Fluor 594 (A-21207, Invitrogen); anti-rat Alexa-Fluor 488 (A-21208, Invitrogen); anti-guinea pig Alexa-Fluor 647 (706605148, Jackson ImmunoResearch); anti-rabbit Alexa Fluor 555 (A-31572, Invitrogen); anti-rabbit Alexa-Fluor 647 (A-31573, Invitrogen); anti-goat Alexa-Fluor 488 (A-11055, Invitrogen); anti-goat Alexa-Fluor 647 (A-21447, Invitrogen); anti-rat Alexa-Fluor 647 (ab150155, Abcam) for IHC.
For immunoblotting: anti-TIMP2 (D18B7, Cell Signaling, 1:5000), anti-actin (A5060; Sigma; 1:10k)

Validation

Antibodies validated by vendors (and in previous publications) were purchased. Validation using KO samples was performed for TIMP2 antibody, as described in figures of the manuscript.

Animals and other research organisms

Policy information about studies involving animals; ARRIVE guidelines recommended for reporting animal research, and Sex and Gender in Research

Laboratory animals

All mice were maintained on C57Bl/6J background. TIMP2 -/- (KO) mice were purchased from Jackson Laboratory, and 5xFAD mice were purchased from MMRRC and subsequently maintained in-house alongside WT littermates. Timp2fl/fl mice were generated as previously described (Ferreira et al., 2023) and crossbred with Cx3cr1CreERT2/+Litt mice or SynCre/+ lines (Jackson). Aged WT C57BL/6 mice were obtained from the National Institute on Aging aged rodent colony.

Wild animals

This study did not involve wild animals.

Reporting on sex

We did not observe sex differences; experiments were sex-matched (males and females), unless otherwise specified in figure. legends.

Field-collected samples

This study did not involve samples collected from the field.

Ethics oversight

Animal procedures, care, and handling were performed in accordance with the NIH Guide for Care and Use of Laboratory Animals and the Icahn School of Medicine at Mount Sinai Institutional Animal Care and Use Committee.

Note that full information on the approval of the study protocol must also be provided in the manuscript.

Plants

Seed stocks

This study did not involve plants.

Novel plant genotypes

This study did not involve plants.

Authentication

This study did not involve plants.
