# Supplementary material for: Youth-associated protein TIMP2 regulates microglial state and function in healthy and aged mice
Source: Nat Commun. 2026 Aug 12;17:8173. doi: 10.1038/s41467-026-74906-z (PMC13469711; doi:10.1038/s41467-026-74906-z)
Supplement: Supplementary file 3 [file 41467_2026_74906_MOESM3_ESM.pdf]

nature portfolio

Peer Review File

Youth-associated protein TIMP2 regulates microglial state and function in healthy and aged mice

Corresponding Author: Dr Joseph Castellano

This file contains all reviewer reports in order by version, followed by all author rebuttals in order by version.

Version 0:

Reviewer comments:

Reviewer #1

(Remarks to the Author)
The authors build on their previous work on TIMP2 as a protective factor against aging, here focusing on its effect on microglia. They use global, microglial, and neuronal TIMP2 KO mice to show that TIMP2 loss increases IBA1 and CD68 expression in vivo, increases inflammatory protein expression in vivo, changes gene expression in vitro, and impairs myelin phagocytosis in vitro. They then treat aged mice (20mo) with exogenous TIMP2 and show that it decreases IBA1 and CD68, alters microglial gene expression, and reduces microglial synaptic phagocytosis. The paper is well done and uses a variety of powerful methods and mouse models. The concept is important, as TIMP2 has therapeutic promise to counteract brain aging, and microglial function is a new wrinkle. My main criticism is that the TIMP2 KO mice are not shown to have a real pathological phenotype in vivo, just some mild changes in microglia markers and modest changes in protein expression. Fig. 5 is the most exciting part of the paper, as it shows improvement in some age-related microglial phenotypes, including synaptic phagocytosis. While it is unreasonable to suggest aging microglial TIMP2 KO mice to 20mo, it would be nice to see an in vivo phenotype such as synaptic phagocytosis to demonstrate that microglial TIMP2 KO has meaningful impact on brain health. Also, the effect on phagocytic function is confusing, as CD68 expression would suggest increased phagocytosis, though decrease myelin phagocytosis is shown with KO, and increase phagocytosis demonstrated with TIMP2 treatment in old mice.
Specific comments:
1. Fig. 1g-f: Does TIMP2 KO increase CD68 expression in vitro? This figure seems a bit odd.
2. TIMP2 deletion consistently increases CD68 expression, suggesting a pro-phagocytic effect. However, myelin phagocytosis is impaired. Is myelin the outlier, i.e. is there a pro-phagocytic effect of TIMP2 deletion in vitro for other molecules, such as when treated with zymosan beads, or labeled BSA? The overall effect on microglial phagocytic activity needs to be clarified. Injection of mylein into the brain, or examining micorlgial phagocytosis of myelin after injury (such as lysolecithin) would also be great, but perhaps not critical.
3. Fig. 5p,q shows a change in microglial synaptic phagocytosis with TIMP2 treatment. It would be very helpful to examine the reciprocal effect in TIMP2 KO mice. Is there an increase in synaptic phagocytosis in TIMP2 ko mice (microglial or global)? This is particularly relevant considering the authors previous paper on TIMP2 and synpases, and some comment on the relative contributions of the ECM vs. microglial mechanisms on synaptic integrity would be welcome.
4. Fig. 2h-l might be better in the Supplement, as they are just validating the method and use 5xFAD mice, which seem out of place.

Reviewer #2

(Remarks to the Author)
Youth-associated protein TIMP2 alters microglial state and function in the context of aging.

Hemmer et al.

In this manuscript, the authors lay out sets of in vivo and in vitro experiments to better understand the role of TIMP2 in the context Alzheimer's disease and its correlation to aging. By performing RNAseq and comparing WT to TIMP2-KO mice, the microglia in TIMP2-KO have an "immunologically" activated state, are pro-inflamatory and have reduced phagocytosis in normal mice. Re-administration of TIMP2 appears to reverse these phenotypes, with the authors concluding that the

presence of TIMP2 keeps microglia in a non-active state and having a more neural protective role.

Overall, this manuscript is interesting and provides additional insight regarding the role of TIMP2. The experiments are well controlled and in general well thought out.

General observations:
- The introduction seems a bit boiler-plate.
- Statistical significance: in some cases (fig 1 and 4), it is indicated that "… is slightly increased/trend to increase without reaching statistical significance". These statements should be avoided, especially when there is high variability in the samples.
- Not sure what the contribution of all the omics data is other than informative, and how it makes the story stronger. Could the authors use some of these data to examine the mechanism in more depth?

Specific observations:
Line 25: neuro-de-generative?
Line 50: AD, does this refer to Alzheimer's? If so, the segue from introduction, especially line 46-47 about the loss of newborn neurons should contain some differencs between mouse and humans.
Lines 58-61: strange sentence
Lines 71-81 reference a recent study about TIMP2 variants and cognitive ability is a preprint and has not been peer reviewed. As such, this should be mentioned in the text. Note that in the preprint, "After stratifying for Aβ status, significance did not survive FDR p-value correction for multiple comparisons, although the association was significant at nominal level and group-correction, respectively." This was done in at-risk patients of AD and not necessarily an age-related comparison.

Figure 1
Mention of pups' and adult mice age in the text, not just in legend, would be more informative for readers.
Any differences observed with sex?
At what age (pups? adult?) was the RNA-seq performed?
At which day after plating were the cells stained? The isolation process could increase the activation state. Why not directly stain adult mice in vivo, especially in the hippocampus, instead of culturing microglia isolated from adult mice?
1e: Note that the isolated microglial were cultured for several days before processing for RNAseq, and not sure if this procedure confounds the results. Also, when reading the methods, it appears that DEGs were chosen based on a P-value < 0.05. The DEGs should be chosen based on and adjusted p-valude (or FDR which is similar).

Figure 2 and Suppl Fig 2
Suppl Fig2 a,b: CD68 staining is very dim and not very convincing. Also, how was the coverage measurement (Suppl Fig2b) done and was it based on those same images? thiscould be problematic. In the same graph, it seems that only 3 mice in the KO group are higher than the rest and also was is the mean value? This result doesn't seem strong enough to really indicate increased microgliosis.
What does the ramified and ameboid morphology represent and what is the conclusion from the observation that there is no change in these types?
Since 2h and 2i provide information to set a baseline, they would fit better as supplementary material. Also, it would be important to also use LPS in WT mice as a control in this experiment.

Figure 3
What is the significance of increased p16+ cells in Cx3cr1CreERT2/+;Timp2fl/fl mice compared to ctrl?

Figure 5
Same comment as Suppl Fig2 a,b.
What about the effect of TIMP2 on the different morphologies (activated, ramified and ameboid) like in Fig.2?
Fig 5p, it would be nice to see orthogonal projections to confirm phagocytosis. In a more general way, it would be important to see functional or real-time assays to prove phagocytosis.

Major concerns:
- The assays and experiments performed are well-conducted and thought out, especially Figure 4. However, the outlook is descriptive and the question remains as to by which mechanism TIMP2 regulates microglial function.

- As the authors mentioned AD in the introduction and used 5xFAD mice in some experiments, it raises the question: does TIMP2 reduce inflammation in 5xFAD mice?

- In combination with the previous report (Ferreira et al. 2023), how does TIMP2 perform a distinct function in neurons and/or microglia?

Reviewer #3

(Remarks to the Author)
In this manuscript, Hemmer et al. build on their previous work on the youth-associated factor TIMP2, focusing on its role in regulating microglial states in adult and aged mice. TIMP2 is upregulated in reactive microglial states and in

neurodegenerative diseases. Given this connection and the well-established role of microglia in aging and age-related diseases, the research question is significant. The authors employ both a global Timp2 knockout and a microglia-specific conditional knockout to dissect the contribution of microglial-derived versus neuronal TIMP2 in modulating microglial gene expression and phagocytic function. Their use of microdialysis to assess cytokine changes in the extracellular space is particularly interesting. These loss-of-function experiments are accompanied by TIMP2 administration to aged mice, which reduces microglial reactivity and inflammation, and enhances synaptic engulfment—though these effects may be indirect. Overall, the study presents several interesting findings with implications for aging. That said, some of the validation experiments using cultured microglia should be complemented by histological analyses on tissue sections, and the microglial state changes in KO mice should be characterized more thoroughly. Please see below for specific comments:

Major:
1. The authors should demonstrate TIMP2 expression in microglia from different brain regions. Currently, only hippocampal microglia are shown (Supplemental Fig. 1c).
2. The quantification of microglial morphology appears somewhat arbitrary and the images used are of low-resolution (Supplemental Fig. 2f, g). The authors should include direct measurements of soma size, branch number, etc., from tissue sections.
3. Expression of TIMP2 was only validated in cultured microglia, which do not fully represent endogenous microglia. Validation by IHC or RNA in situ on brain sections is necessary. Additionally, the nature of the KO allele should be clarified —does it result in a null mutation? Why are some transcripts still detectable in the global KO mice (Figure 1d)?
4. The overlap between TIMP2 and CD68 signals was only shown in cultured microglia (Figure 1f, g). This should be replicated using IHC or RNA in situ in vivo. The percentage of TIMP2+ and TIMP2+/CD68+ microglia in vivo, and any regional differences, should be quantified and reported.
5. What is the magnitude of CD68 upregulation in CX3CR1-CreER conditional KO mice compared to the global KO? Does microglia-specific deletion of TIMP2 produce a similar or milder phenotype? This should be quantified using an adequate number of animals and tissue sections.
6. Since senescent microglia are difficult to define due to the lack of specific markers, p16 staining alone is insufficient. Additional orthogonal validation is required.
7. To illustrate microglial state regulation by TIMP2, the current evidence (bulk RNA-seq from cultured microglia, IHC for CD68 and p16, and snRNA-seq after TIMP2 injection) is not sufficient. Cultured microglia may not reflect in vivo states; CD68 and p16 are nonspecific; and TIMP2 injection is a gain-of-function approach. For the bulk RNA-seq, only GO terms were discussed, with no specific gene-level analysis. The authors should perform transcriptomic profiling on microglia isolated from Timp2 KO (preferably conditional KO) mice and compare this with the bulk RNA-seq and snRNA-seq datasets.
8. Marker genes from key microglial clusters identified in the snRNA-seq analysis should be presented. Currently, only GO terms are shown. Changes in some of the key clusters should also be validated via IHC or flow cytometry.
9. Engulfment of synaptic particles was assessed only in TIMP2-injected mice. The same analysis should be performed in TIMP2 KO mice to complete the comparison.

Minor:
1. Line 118–119: If P5–P8 mice were used, they should be described as early postnatal (as opposed to neonatal), and the exact age range should be noted.
2. Line 247–248: Clarify what is meant by "time-dependent fashion." Does TIMP2 accumulate cumulatively in culture, or does secretion increase over time?
3. Line 228: The correct reference should be to Supplemental Figure 1e.
4. Figure 5i, j, k: Cluster identities should be labeled directly on the figures to aid interpretation.

Reviewer #4

(Remarks to the Author)
Summary: The goal of this manuscript is to explore how the age-rejuvenating factor, TIMP2, exerts its protective effects on the brain. This paper is a follow up to the group’s previous work to show improvements in synaptic function and learning and memory with TIMP2, but the first of its kind to show that TIMP2 plays an unappreciated role in microglial function and neuroinflammation. Using complimentary approaches including TIMP2 deletion (global or cell type specific) as well as TIMP2 treatment showed that bidirectionally modulating TIMP2 levels has profound effects on microglia, which is novel function for this protein. This study is rigorously done and insights from this work are important for aging and age-related neurodegenerative diseases. This study is particularly noteworthy given the translational value of these studies. For example, the TIMP2 treatment study in 20 mo. old mice that clearly shows TIMP2’s protective effect that is lost with aging.

While this reviewer maintains strong enthusiasm for this work, there are some minor points that need clarification:

1. Line 47, 95, 136: There has been a recent push for consistency of microglia nomenclature (PMID: 36327895), which adds some confusion to what different terms may mean. Terms like “microgliosis” or “activation” are sometimes hard to interpret, or perceived to be outdated. In the introduction, “microgliosis” or “activation” is used when describing changes in microglia due to aging. Can the authors clarify or define these terms for the reader to avoid confusion? Does aging have its own microglial phenotype that is different than disease associated phenotypes typically described in fields like Alzheimer’s? Given the lack of consensus in the field, it might be better to say “microglia” instead of “microglial state”, since it’s unclear what “microglial state” means in this paper and whether TIMP2 expression in microglia modulates its own "microglial state" since now all microglia are TIMP2+.

2. Not all TIMP2+ microglia are CD68+ and not all CD68+ microglia are TIMP2+. Do the authors know why this is? What other markers does the TIMP2+ microglia express (e.g. homeostatic v disease reactive) that may help provide understand why TIMP2 is important in the setting of aging? Can the authors speculate?

3. Fig 2: this is the global knockout, correct? Please explicitly state that.

4. Fig 2b-c: what does each circle represent on the bar graphs? It should represent each mouse but the figure legend says sample size n=9-13 but there are more circles than that. Can you clarify?

5. Line 156-157: Since there is no increase in total number of microglia, does this suggest in increase in cell size? Was this quantified?

6. Line 179: The statement “This technique has not yet been extensively applied to measure brain immune proteins” should be removed or tempered. Other groups, PMID: 27481936 for example, have used in vivo microdialysis to investigate the ISF proteome and showed enrichment for inflammatory related proteins relative to Alzhiemer’s disease and sleep deprivation.

7. Figure 3: The white arrows in panel b and d are very distracting. I’d recommend removing.

8. Line 237: Consider change “external pools” to “ISF” or “extracellular” pools.

9. Line 249: Clarify what zymosan does and why it was chosen? Are both zymosan and LPS used as an inflammatory challenge? Could the lack of effects be due to dose chosen?

10. Figure 5: Did TIMP2 treatment in vitro increase total # of Iba1+ microglia?

Version 1:

Reviewer comments:

Reviewer #1

(Remarks to the Author)
The authors have addressed my concerns. THey have provided a thoughtful response to the data showing increased CD68 and decreased myelin phagocytosis, and and tried phagocytosis assays with another substrate, and examined synpatic phagocytosis in KO mice. I think the paper is strong and provide some unique insights into TIMP2 as a potential mediator/preventer of age-related microglial dysfunction.

Reviewer #2

(Remarks to the Author)
This reviewer has no further comments. The authors have done an excellent job addressing all comments from the initial review, and the manuscript is now clear, rigorous, and presents a very strong and cohesive story.

Reviewer #3

(Remarks to the Author)
The authors have addressed all my concerns.

Reviewer #4

(Remarks to the Author)
The authors have been responsive to all reviewer's concerns. The manuscript has been sufficiently improved. No new concerns remain.

Open Access This Peer Review File is licensed under a Creative Commons Attribution 4.0 International License, which permits use, sharing, adaptation, distribution and reproduction in any medium or format, as long as you give appropriate credit to the original author(s) and the source, provide a link to the Creative Commons license, and indicate if changes were made.
In cases where reviewers are anonymous, credit should be given to 'Anonymous Referee' and the source.
The images or other third party material in this Peer Review File are included in the article's Creative Commons license, unless indicated otherwise in a credit line to the material. If material is not included in the article's Creative Commons license and your intended use is not permitted by statutory regulation or exceeds the permitted use, you will need to obtain permission directly from the copyright holder.
To view a copy of this license, visit https://creativecommons.org/licenses/by/4.0/

REVIEWER COMMENTS

We would like to thank all reviewers for their positive and insightful reviews of our manuscript. All reviewers highlighted the experimental rigor, powerful methods, and translational value of characterizing the novel impact of TIMP2 on microglia function in aging. We have taken every effort to revise the manuscript based on the constructive feedback provided by reviewers, and we feel the revised manuscript convincingly elucidates an unexplored role of TIMP2 in modulating microglia function relevant to detrimental aging phenotypes.

Reviewer #1 (Remarks to the Author):

The authors build on their previous work on TIMP2 as a protective factor against aging, here focusing on its effect on microglia. They use global, microglial, and neuronal TIMP2 KO mice to show that TIMP2 loss increases IBA1 and CD68 expression in vivo, increases inflammatory protein expression in vivo, changes gene expression in vitro, and impairs myelin phagocytosis in vitro. They then treat aged mice (20mo) with exogenous TIMP2 and show that it decreases IBA1 and CD68, alters microglial gene expression, and reduces microglial synaptic phagocytosis. The paper is well done and uses a variety of powerful methods and mouse models. The concept is important, as TIMP2 has therapeutic promise to counteract brain aging, and microglial function is a new wrinkle. My main criticism is that the TIMP2 KO mice are not shown to have a real pathological phenotype in vivo, just some mild changes in microglia markers and modest changes in protein expression. Fig. 5 is the most exciting part of the paper, as it shows improvement in some age-related microglial phenotypes, including synaptic phagocytosis. While it is unreasonable to suggest aging microglial TIMP2 KO mice to 20mo, it would be nice to see an in vivo phenotype such as synaptic phagocytosis to demonstrate that microglial TIMP2 KO has meaningful impact on brain health. Also, the effect on phagocytic function is confusing, as CD68 expression would suggest increased phagocytosis, though decrease myelin phagocytosis is shown with KO, and increase phagocytosis demonstrated with TIMP2 treatment in old mice.

Response: We thank the reviewer for highlighting the conceptual importance of our study and noting the technical strengths, including our use of powerful methods and multiple mouse models and for kindly stating that the manuscript was well done. The reviewer's main criticism and other minor critiques are addressed below.

Specific comments:
1. Fig. 1g-f: Does TIMP2 KO increase CD68 expression in vitro? This figure seems a bit odd.

Response: We have now revised the figure to emphasize the focus on expression of TIMP2 within microglia in WT mice (and in brain tissue) and the observation in KO microglia that certain programs may be altered with TIMP2 deletion using bulk RNAseq. To focus and balance limited resources on a number of critiques raised, we did not examine CD68 expression in an in vitro setting in TIMP2 KO microglia since we report this phenotype in vivo (**Fig. 2**), as well as in vitro using targeted deletion of TIMP2 in microglia (e.g., **Fig. 3**). TIMP2 KO mice are unfortunately difficult to produce since they must be bred as heterozygous pairings (KOs do not breed), and even heterozygous TIMP2 breeders are inefficient, leading to a small number of litters with many breeding cages. Since we must produce WT and KO littermates for such an experiment, and the mice must be generated for the same day of culturing, significant practical considerations challenge such an experiment in order to obtain enough cells to plate for culturing, staining, biological replicates, and appropriately powered quantification. However, the figure has now been re-focused to describe expression of TIMP2 in primary microglia, as well as in adult brain tissue (**Figure 1k-m**) and in several brain regions (**Supp. Fig. 1a-g, p-r**), as well as further analysis of our bulk RNAseq data (since these samples could be frozen across collections before being sequenced and analyzed together). Bulk RNAseq components of this figure were expanded by performing additional analyses (**Fig. 1d-g; Supp. Fig. 1 h, j-l**), including enrichment

analyses, WGCNA, and functional description of individual top altered genes, which together support an increase in activation in KO microglia, a phenotype supported in subsequent figures in the manuscript in vivo and in vitro with targeted TIMP2 deletion. We also use WT adult mice in **Fig. 1i-j** to illustrate the co-localization of TIMP2 with CD68, which is further corroborated using adult WT brain tissue (**Fig. 1n-o**), as an entry point to exploring TIMP2's association with lysosomal function and state. We examined changes in microglia expressing CD68 *in vivo* (**Fig. 2 and Fig. 3**) to substantiate our claim that loss of TIMP2 alters their state.

2. TIMP2 deletion consistently increases CD68 expression, suggesting a pro-phagocytic effect. However, myelin phagocytosis is impaired. Is myelin the outlier, i.e. is there a pro-phagocytic effect of TIMP2 deletion in vitro for other molecules, such as when treated with zymosan beads, or labeled BSA? The overall effect on microglial phagocytic activity needs to be clarified. Injection of mylein into the brain, or examining micorlgial phagocytosis of myelin after injury (such as lysolecithin) would also be great, but perhaps not critical.

Response: Thank you for raising this point. We agree that the overall effect of TIMP2 on microglia phagocytosis can be clarified in our manuscript. To examine whether TIMP2 deletion affects phagocytosis of other substrates, we repeated the previous assay performed for myelin, this time using pHrodo-labeled zymosan, as suggested. Adult microglia isolated from tamoxifen-treated *Cx3cr1$^{CreERT2/+}$;Timp2$^{fl/fl}$* and *Timp2$^{fl/fl}$* controls were treated with pHrodo-labeled zymosan and uptake was monitored, revealing no significant difference for this particular substrate (**Suppl. Fig. 4a-c**). Other work has shown that microglia often exhibit substrate-specific phagocytic preferences[1] and that differing substrates induce unique transcriptional states[2], supporting the notion that microglia, especially in diverse subtypes, do not respond to all substrates equally. Across our manuscript, our data are consistent across at least 2 types of KO and treatment contexts (and see response to below comment), arguing that TIMP2 affects microglial phagocytosis in certain contexts. We have added the reviewer's suggestion to further explore TIMP2's effect on microglial uptake of myelin in the setting of injury to the discussion (below).

"…future work will need to identify the directionality of these changes following treatment with TIMP2 and related factors and the extent to which phagocytosis of varied types of debris is affected and under what contexts, including injury and other models."

While CD68 is sometimes interpreted as a proxy for phagocytosis, it does not always directly correlate with levels of phagocytic activity in myeloid cells, as one study found that macrophage phagocytosis was normal in CD68 KO mice[3], and others have reported increased CD68 alongside reduced phagocytic clearance[4], including a recent study showing elevated CD68 in lipid-accumulating microglia with reduced phagocytosis[5]. Instead, changes in CD68 can reflect changes in lysosomal function[6] or autophagy[7]. Our data consistently show increased number of CD68$^+$ microglia when TIMP2 is deleted, alongside reduced phagocytosis (also see experiment addressing point #3 below). The increase in CD68$^+$ microglia with reduced phagocytosis may reflect delayed ability of lysosomes to degrade materials, consistent with reduced myelin clearance by microglia we observed (**Fig. 4j-k**). In settings of faulty phagocytosis, cells may upregulate CD68 as a compensatory response to handle cargo/debris. We also note that we observed elevated hippocampal ISF levels of lysosomal serine protease TPP1 in TIMP2 KO mice, a phenotype observed in glia from AD subjects or those with lysosomal storage disorders[8]. We have added brief discussion of some of these points in the discussion section (lines 513-522) to highlight these possibilities.

3. Fig. 5p,q shows a change in microglial synaptic phagocytosis with TIMP2 treatment. It would be very helpful to examine the reciprocal effect in TIMP2 KO mice. Is there an increase in synaptic phagocytosis in TIMP2 ko mice (microglial or global)? This is particularly relevant considering the authors previous paper on TIMP2 and synpases, and some comment on the relative contributions of the ECM vs. microglial mechanisms on synaptic integrity would be welcome.

Response: Thank you for this excellent suggestion. We have followed the reviewer's suggestion to examine microglial synapse phagocytosis in TIMP2 KO mice in order to address the main criticism stated by the reviewer to examine another phenotype in KO mice. Interestingly, using the same approach utilized for TIMP2 treatment, we found that microglia in TIMP2 KO dentate gyrus have significantly less Vglut1^{+} puncta within lysosomes relative to microglia in WT mice (**Fig. 2g-h**). Combined with our finding that TIMP2 treatment restores phagocytic capacity of aged microglia, these data support our overall claim that TIMP2 affects microglial phagocytosis of synaptic puncta in a reciprocal fashion. This observation also supports our in vitro data in which we observe diminished phagocytic uptake or clearance (for myelin) in microglia lacking TIMP2. We reported previously that dendritic spine complexity in the DG is significantly reduced in the setting of TIMP2 deletion[9], which could reflect a separate mechanism in which TIMP2 directly affects ECM accumulation to affect spine dynamics and homeostasis. Healthy microglia in the setting of higher TIMP2 levels and in a less inflammatory environment may also remove certain types of synapses more efficiently, better supporting overall spine complexity. There is a growing literature arguing that ECM and microglia interact to affect spine dynamics[10], raising the possibility of an indirect mechanism by which neurons are altered by microglial behavior on ECM within the microenvironment, though we hope to further explore these relationships and roles for TIMP2 in future work. We have included these possibilities and our speculation as a comment, as suggested by the reviewer, in the discussion (lines 455-461).

4. Fig. 2h-I might be better in the Supplement, as they are just validating the method and use 5xFAD mice, which seem out of place.

Response: We thank the reviewer for this suggestion, as we agree these particular mice are meant only for validation and not a main focus of the paper. We have moved the LPS HMWCO *in vivo* microdialysis validation experiment to the supplement, as suggested, which can now be found in **Supplementary Figure 2l-m**.

Reviewer #2 (Remarks to the Author):

Youth-associated protein TIMP2 alters microglial state and function in the context of aging.

Hemmer et al.

In this manuscript, the authors lay out sets of in vivo and in vitro experiments to better understand the role of TIMP2 in the context Alzheimer's disease and its correlation to aging. By performing RNAseq and comparing WT to TIMP2-KO mice, the microglia in TIMP2-KO have an "immunologically" activated state, are pro-inflamatory and have reduced phagocytosis in normal mice. Re-administration of TIMP2 appears to reverse these phenotypes, with the authors concluding that the presence of TIMP2 keeps microglia in a non-active state and having a more neural protective role.

Overall, this manuscript is interesting and provides additional insight regarding the role of TIMP2. The experiments are well controlled and in general well thought out.

Response: We appreciate the reviewer noting that the manuscript is "interesting, provides additional insights, is well-controlled and is well thought out". We have addressed the concerns as outlined below.

General observations:
- The introduction seems a bit boiler-plate.

Response: We appreciate this comment and have revised the introduction to provide additional nuance for the project, as highlighted throughout the introduction in yellow.

- Statistical significance: in some cases (fig 1 and 4), it is indicated that "… is slightly increased/trend to increase without reaching statistical significance". These statements should be avoided, especially when there is high variability in the samples.

Response: We appreciate that this can add confusion. We have revised instances of trending effects to clarify that these effects did not reach statistical significance or have removed other instances entirely.

- Not sure what the contribution of all the omics data is other than informative, and how it makes the story stronger. Could the authors use some of these data to examine the mechanism in more depth?

Response: We thank the reviewer for this point and agree that enhancing our analysis of RNAseq data will provide more insights into the mechanism of TIMP2-induced microglial alterations driving relevant phenotypes. Given our findings that TIMP2 treatment affects microglial phagocytosis of synaptic puncta (**Fig. 5w-x**) we aimed to examine if microglial-neuronal interactions are altered following TIMP2 treatment. As such, we performed Cell-Chat analyses on our snRNAseq dataset and found that hippocampal microglia increase their number of interactions with neurons relative to vehicle-treated microglia (**Fig. 5s**). We further probed what microglial-neuronal signaling pathways were altered between groups and found that TIMP2 treatment downregulated glutamate signaling pathways and associated ligand-receptor pairs, while increasing communication probability for ADGRL signaling[11,12] (**Fig. 5t-v**), an adhesion GPCR linked to signaling pathways altered in phagocytosis. In deleterious contexts, microglia have been shown to exhibit perturbed glutamate signaling leading to neurotoxicity[13] and changes in synapses[14]. These analyses point to interesting potential mechanisms involving microglial-neuronal interactions to be explored in future directions.

Specific observations:
Line 25: neuro-de-generative?

Response: Thank you for catching this typo, which we have now corrected to "neurodegenerative" (line 25).

Line 50: AD, does this refer to Alzheimer's? If so, the segue from introduction, especially line 46-47 about the loss of newborn neurons should contain some differencs between mouse and humans.

Response: Yes, AD in the Intro referred to Alzheimer's disease (which we now define at first appearance), but we have revised this segue in revision of the Introduction to more broadly discuss neurodegeneration and aging. We have focused on changes to the immune environment in aging and AD, as opposed to loss of newborn neurons that we had included earlier, to focus on the predominant phenotypes discussed throughout the paper.

Lines 58-61: strange sentence

Response: We have revised this sentence to enhance readability (lines 55-60).

Lines 71-81 reference a recent study about TIMP2 variants and cognitive ability is a preprint and has not been peer reviewed. As such, this should be mentioned in the text. Note that in the preprint, "After stratifying for Aβ status, significance did not survive FDR p-value correction for multiple comparisons, although the association was significant at nominal level and group-correction, respectively." This was done in at-risk patients of AD and not necessarily an age-related comparison.

Response: Since our initial submission, the cited study has been published in *Neurobiology of Aging*. We have adjusted the citation to reflect this. We have also revised the text (lines 77-78) to qualify that this study was conducted on aged individuals at increased risk for developing AD.

Figure 1
: Mention of pups' and adult mice age in the text, not just in legend, would be more informative for readers.

Response: We agree this would be more informative readers and have incorporated age further into the text and ensured this is clear in all figure legends.

: Any differences observed with sex?

Response: For experiments involving early postnatal microglia in Figure 1, samples from both sexes had to be pooled, making it impossible to dissect any effects of sex. However, we took careful measures to sex-match in this figure and wherever feasible in adult mice across the manuscript, and we did not observe sex differences, which is consistent with our previous work in adult mice lacking TIMP2 in other CNS phenotypes[9].

: At what age (pups? adult?) was the RNA-seq performed?

Response: RNA-seq was performed using pups P6-P7. Beyond inclusion in the figure legend, we have added these details to the main text to clarify this point, as seen in line 120.

: At which day after plating were the cells stained? The isolation process could increase the activation state. Why not directly stain adult mice in vivo, especially in the hippocampus, instead of culturing microglia isolated from adult mice?

Response: Some isolation methods can affect activation state, so we have taken careful steps in our experimental methods for isolation to minimize the impact of the isolation process on activation state, including performing *in vitro* isolations on ice, which significantly minimizes this activation[15]. Additionally, functional experiments were performed according to established protocols in which adult microglia are plated and allowed to recover for ~7 days to minimize induced activation[16,17] and imaged immediately following this recovery period. We used cell culture assays in this study to better facilitate assay of functional endpoints, and we validated findings in mouse hippocampus wherever possible. To address the reviewer's suggestion in Figure 1 that the staining from cell culture should be performed in adult hippocampus, we validated TIMP2 staining in brain tissue from adult mice. We used immunohistochemistry and confocal imaging for anti-TIMP2 and anti-CD68 staining of microglia in hippocampus, finding that ~80% of TIMP2-positive microglia are CD68-positive (**Fig. 1k-o**), which was similar to our previous in vitro results, giving us confidence in both systems and our overall conclusions.

: 1e: Note that the isolated microglial were cultured for several days before processing for RNAseq, and not sure if this procedure confounds the results. Also, when reading the methods, it appears that DEGs were chosen based on a P-value < 0.05. The DEGs should be chosen based on and adjusted p-valude (or FDR which is similar).

Response: As mentioned above, we used an *in vitro* protocol[16] that minimizes artificial activation for isolated microglia, which involves several days in culture prior to collection for RNAseq. We appreciate the reviewer's comment regarding use of nominally significant DEGs, which were used in the initial submission as input for P-adjusted enriched pathways for hypothesis generation. To address the concern, we calculated adjusted *P* values using the conventional Benjamini-Hochberg procedure (P_{adj} < 0.05; now represented as volcano plot; **Supplementary Fig. 1h**). Gene ontology over-representation pathway analyses for biological processes (BP) using these P_{adj} genes reveal very similar terms as in our initial analysis, including processes related to cytokine signaling, cell activation, and immune system processes (**Supplementary Fig. 1j)**. To further explore the dataset, we used ranked GSEA and Weighted Gene Correlation Network Analysis (WGCNA) approaches that use all genes in the dataset to examine enrichment, or modules identified in an unsupervised way using all genes and their pairwise expression correlations, respectively, to identify enriched pathways. GSEA on all genes ranked according to the Wald statistic from Deseq2 at P_{adj} < 0.05 revealed that TIMP2 KO microglia exhibit downregulated processes related to synapses and neuronal differentiation and upregulated processes involved in cell activation involved in immune response, regulation of cytokine production, and immune system processes (**Fig. 1d**) as seen in previous approaches. Our WGCNA approach identified several significant modules, including a downregulated cyan module and upregulated red module in TIMP2 KO vs WT microglia (**Fig. 1e**). Over-representation analyses on genes extracted from the cyan module revealed changes in pathways related to synapses and generation of neurons (**Fig. 1f**), while red module genes reflected enrichment in pathways that included cell activation and immune system processes in KO microglia (**Fig. 1g**). Our updated and more robust analyses have been added to the manuscript, supporting overall our claim that TIMP2 deletion is associated with processes linked to increased microglial activation, which is broadly corroborated using subsequent approaches in the manuscript across in vitro and in vivo models.

Figure 2 and Suppl Fig 2

: Suppl Fig2 a,b: CD68 staining is very dim and not very convincing. Also, how was the coverage measurement (Suppl Fig2b) done and was it based on those same images? thiscould be problematic. In the same graph, it seems that only 3 mice in the KO group are higher than the rest and also was is the mean value? This result doesn't seem strong enough to really indicate increased microgliosis.

Response: Given the young age of the mice in the figure referenced by the reviewer, low levels of CD68 are expected, as seen for this marker in other studies at similar ages[18-22], reflecting that microglia exhibit low levels of activation and may be mostly homeostatic at this age[23]. The CD68 coverage measurement

in Supp. Fig. 2b uses the same tile-scanned 40x confocal images across Fig. 2a-b and Suppl. Fig. 2a-c but applies a uniform threshold based on visible CD68 staining in the images across all mice, yielding thresholded area as a percentage of total area quantified. The mean % CD68+ coverage for WT mice is 0.082, while the KO mean is 0.1346, a result that was statistically significant. Examining the variance noted by the reviewer in Supp. Fig. 2b, the result remained statistically significant even after the unequal variance was accounted for with Welch's correction. Although the lower levels are expected for mice at this young age, the reported change is suggestive of a change in CD68 with TIMP2 deletion, along with other changes in the same age in Fig. 2, though we understand that the weaker effect may warrant caution. When we examine slightly older mice in which baseline CD68 expression is higher, as expected (**Figure 2d-f**), we see a statistically significant difference between groups that is broadly observed through other measures in the manuscript. We have altered language for improved clarity and caution in the interpretation of CD68 staining in younger mice in 3-4-month-old mice (lines 165-171).

: What does the ramified and ameboid morphology represent and what is the conclusion from the observation that there is no change in these types?

Response: We agree with the reviewer that interpretation of what these states may functionally represent is not clear in the manuscript, which somewhat reflects the somewhat mixed understanding of these links across studies in the field[24,25]. Our analysis had initially assigned microglia into these states broadly using a calculated circularity index, which tries to capture overall activation state. To improve our approach for increased rigor, we endeavored to perform a more time-intensive, detailed characterization of microglia morphology in TIMP2 WT and KO hippocampi using Imaris 3D-based reconstruction from confocal images. This is superior to our initial broad classification of microglia morphology into “ramified”, “activated” or “ameboid”, so that initial analysis has been removed. Imaris-based analysis revealed that TIMP2 KO microglia exhibit increased volume and surface area relative to WT microglia (**Supplementary Fig. 2g-h**), a phenotype linked to transition from ramified to reactive/primed and hypertrophic microglia in other conditions[26-29]. We also observed increased oblate ellipticity (**Supplementary Fig. 2i**), a flattened phenotype associated with ameboid microglia that may be associated with a pro-inflammatory state[26,27,30]. TIMP2 KO microglia also exhibit a converse decrease (though not statistically significant, P=0.06) in prolate ellipticity (**Supplementary Fig. 2i**) that is associated with more elongated, responsive microglia[31,32]. Branching Sholl intersections were not significantly different (**Supplementary Fig. 2j**). Together, these results indicate that TIMP2 broadly affects microglia morphology, and we have tempered conclusions that link morphology to microglial function (lines 175-181), as there is acknowledged difficulty with strictly interpreting function from morphology within the field.

: Since 2h and 2i provide information to set a baseline, they would fit better as supplementary material. Also, it would be important to also use LPS in WT mice as a control in this experiment.

Response: We agree with the reviewer that data originally shown as Figure 2h-i can be moved to a supplementary figure. This change can be found in line 208 and **Supplementary Fig. 2l-m**. Regarding use of a WT mouse as a control in this experiment, the purpose here was only to validate our ability to detect expected immune-related proteins in the ISF using our microdialysis approach. Since it's well established that 5XFAD mice have detectable immune-related proteins that we could easily perturb with LPS, we settled on this approach as a proof-of-concept, which we agree fits better in the supplementary material. As the proof-of-concept was sufficient to establish that that the tool could be used to measure immune proteins in the ISF, we now include this as supplementary data that support its application in WT vs KO mice in the main text. The technique, including surgeries, sampling, equipment, and O-link measurements is unfortunately costly beyond current resources, so we will explore immune challenges in WT mice in subsequent work and hope that the included data motivate others to pursue the same.

Figure 3

: What is the significance of increased p16+ cells in Cx3cr1CreERT2/+;Timp2fl/fl mice compared to ctrl?

Response: Thank you for pointing out that this was unclear in the manuscript. P16+ staining in these cells reflects staining for p16INK4a, a widely used marker of senescence that we are detecting in our primary microglia from Cx3cr1CreERT2/+;Timp2fl/fl mice as significantly elevated compared to microglia from control mice. To address the reviewer's question, we further validate this finding with other senescence-associated markers, we stained primary cultures from Cx3cr1CreERT2/+;Timp2fl/fl and control mice with markers for p21 (**Supplementary Fig. 3f-g**) and SA-beta-Gal (**Supplementary Fig. 3h-i**), which revealed that these markers are also elevated with TIMP2 deletion. Together, our data consistently show that there are significantly more microglia expressing markers associated with senescence in *Cx3cr1*$^{CreERT2/+}$;*Timp2*$^{fl/fl}$ compared to *Timp2*$^{fl/fl}$ controls, which complements the enrichment in microglial senescence pathways in bulk RNAseq (**Supplementary Fig. 1k**).

Figure 5

: Same comment as Suppl Fig2 a,b.

Response: CD68 staining presents as a somewhat punctated stain due to its labeling of phagolysosomal content. In this figure, given the advanced age of these mice[23], our staining pattern reflects what we typically see and what is observed by other groups at later ages using this marker (more staining and larger area per cell). To address the reviewer's concern of the previous image, we replaced the representative images for Fig. 5e with an improved magnification that better illustrates the staining quantified in this experiment. The broadly consistent pattern of changes we report using this marker in expected directions, depending on the modulation of TIMP2, lend further support to this observation.

: What about the effect of TIMP2 on the different morphologies (activated, ramified and ameboid) like in Fig.2?

Response: As described in our response to the reviewer's earlier comment on morphology related to **Supp. Fig. 2**, we have since removed the circularity-based approach that categorized into activated, ramified, and ameboid and replaced analyses with an Imaris-based approach. For Suppl. Fig. 5, we now applied the more advanced Imaris-based reconstruction approach to assess microglia morphology from TIMP2- or vehicle-treated mice. Using filament tracings of analyzed microglia, we performed Sholl analysis to detect broad morphological changes. A linear mixed model (Intersections ~ Group * poly(Radius,2) + (1 | MouseID/SectionID/CellID)) conducted on Sholl data from many microglia per mouse in each group revealed significant differences in the group x radius interaction in the linear component, with the curve for TIMP2-treated microglia decaying more slowly, suggesting more distal branching in TIMP2-treated mice compared to vehicle-treated mice (**Supplementary Fig. 5d-e**). Increased branching may reflect transition from more ameboid/activated states[26-29]. Analysis of microglial surfaces also revealed that TIMP2-treated microglia exhibit increased prolate ellipticity (**Supplementary Fig. 5f**), a phenotype associated with more responsive microglia under certain conditions[31,32]. Interestingly, this prolate phenotype was in opposite direction to that seen in TIMP2 KO microglia (**Supplementary Fig. 2i**). Additionally, oblate ellipticity from TIMP2 treatment also exhibited opposite direction changes as in KO mice, though it did not reach statistical significance (not shown; P=0.1). Volume was unchanged by TIMP2 treatment (**Supplementary Fig. 5g**), together reflecting modest but consistent morphological changes overall, all of which we have described in lines 324-327.

: Fig 5p, it would be nice to see orthogonal projections to confirm phagocytosis. In a more general way, it would be important to see functional or real-time assays to prove phagocytosis.

Response: Thank you for raising this important suggestion to provide additional functional demonstration of the phagocytosis changes we observed *in vivo* (in what was formerly Figure 5p; now **Figure 5w-x**). To address this, we cultured 20-month-old microglia and treated them with TIMP2 or vehicle for 6hrs, using the same paradigm used in a previous *in vitro* assay in Figure 5, and then incubated the cells with myelin to evaluate the impact of TIMP2 treatment on phagocytosis of myelin by monitoring real-time uptake by Incucyte live imaging. Our new analysis reveals that TIMP2 treatment increases microglial uptake of myelin compared to vehicle-treated microglia (**Supplementary Fig. 5t-v**), further supporting that TIMP2 increases microglial phagocytosis in this context. We have also provided the requested orthogonal projections (**Response Fig. 1**) to confirm Vglut1 puncta present within the lysosomal area of microglia, reflecting what our Imaris analyses captured as reported.

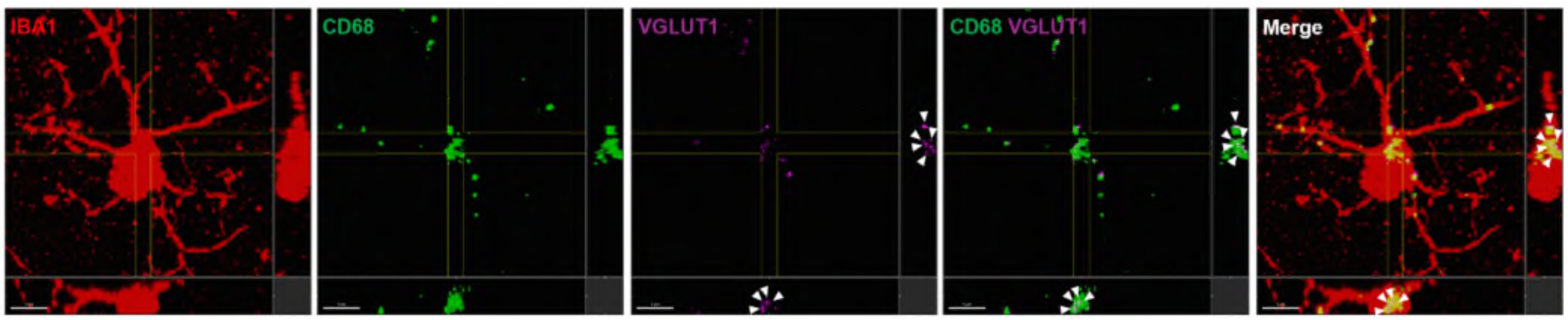

Response Fig. 1: Representative confocal image of DG of 20-month-old mice stained with anti-IBA1, anti-CD68, and anti-VGLUT1 antibodies (scale bar = 5 µm; arrowheads indicate CD68+VGLUT1+ puncta). Orthogonal projections X-Z (bottom left) and Y-Z (right) planes with lines indicating projection plane boundaries.

Major concerns:

- The assays and experiments performed are well-conducted and thought out, especially Figure 4. However, the outlook is descriptive and the question remains as to by which mechanism TIMP2 regulates microglial function.

Response: We appreciate the reviewer's assessment of our manuscript's experiment as "well-conducted and thought out". We agree that there are remaining questions to be explored. Our existing data, supported by additional suggested experiments we now include, support our claims that TIMP2 affects microglia state and phagocytosis using both deletion and supplementation approaches in differing contexts. To provide additional insight into the impact of TIMP2 on microglia, we performed Cell-Chat ligand-receptor pair analysis on our snRNAseq data (**Fig. 5s-v**), which suggested that TIMP2 treatment may decrease the probability of specific interactions between microglia and neurons related to "glutamate signaling", while increasing probability for interactions involved in "ADGRL signaling". Earlier work has shown that when microglial state is altered, glutamate signaling is perturbed, leading to neural dysfunction[13] and synapse loss[14]. ADGRL belongs to a family of adhesion GPCRs, several of which have been shown to negatively affect phagocytosis when signaling is reduced[11,12]. While unraveling precise mechanisms will be important in future work, we have provided evidence that TIMP2 regulates microglia in healthy brain and in the setting of aging, and we have provided additional discussion that speculates on future directions for further dissection of mechanism (lines 455-461).

- As the authors mentioned AD in the introduction and used 5xFAD mice in some experiments, it raises the question: does TIMP2 reduce inflammation in 5xFAD mice?

Response: We agree that looking at TIMP2 in the setting of AD pathology could be a fruitful area of future research that we are currently endeavoring to study in the lab. As the current focus in this manuscript is on the role of TIMP2 on microglial phenotypes in healthy and aged contexts, these

investigations in other pathological contexts were beyond the scope of the current work. To avoid confusion, we softened any language throughout the manuscript that overstates relevance to AD pathology, though minor elements were retained where appropriate as we feel the phenotypes identified here are relevant to AD and other neurodegenerative disorders implicated in altered brain immune function, motivating this concept as future study.

- In combination with the previous report (Ferreira et al. 2023), how does TIMP2 perform a distinct function in neurons and/or microglia?

Response: We thank the reviewer for this question. Results in Ferreira et al., 2023 focused on neuronal effects of TIMP2 on synaptic and functional outcomes, which we argue may be mediated through changes in the extracellular matrix. When considered alongside our current manuscript, our results support a model in which TIMP2 exerts both cell-type-specific effects and cell-intrinsic effects that together may affect the microenvironmental niche under study. As briefly described above, we added Cell-chat analyses that suggest altered microglial-neuronal communication following TIMP2 treatment (**Fig. 5s-v**), supporting one possibility. A growing literature also argues that ECM and microglia interact to affect neuronal function[10], raising the possibility of an indirect mechanism by which neurons are altered by microglial behavior on ECM in the microenvironment, though we hope to further explore these relationships and roles for TIMP2 in future work. We have briefly expanded on these possibilities in our discussion (lines 455-461).

Reviewer #3 (Remarks to the Author):

In this manuscript, Hemmer et al. build on their previous work on the youth-associated factor TIMP2, focusing on its role in regulating microglial states in adult and aged mice. TIMP2 is upregulated in reactive microglial states and in neurodegenerative diseases. Given this connection and the well-established role of microglia in aging and age-related diseases, the research question is significant. The authors employ both a global Timp2 knockout and a microglia-specific conditional knockout to dissect the contribution of microglial-derived versus neuronal TIMP2 in modulating microglial gene expression and phagocytic function. Their use of microdialysis to assess cytokine changes in the extracellular space is particularly interesting. These loss-of-function experiments are accompanied by TIMP2 administration to aged mice, which reduces microglial reactivity and inflammation, and enhances synaptic engulfment—though these effects may be indirect. Overall, the study presents several interesting findings with implications for aging. That said, some of the validation experiments using cultured microglia should be complemented by histological analyses on tissue sections, and the microglial state changes in KO mice should be characterized more thoroughly. Please see below for specific comments:

Response: We thank the reviewer for constructive comments and for highlighting the significance of the research question, the particularly interesting methods used, and our work's implications for aging. We have addressed the issues described below with new experiments and further characterization.

Major:
1. The authors should demonstrate TIMP2 expression in microglia from different brain regions. Currently, only hippocampal microglia are shown (Supplemental Fig. 1c).

Response: We focused on hippocampal microglia due to the susceptibility of this region to age-related decline and our previous work supporting TIMP2's role in regulating hippocampus-associated function[9,33]. In supplemental Figure 1c of the initial manuscript, we showed TIMP2 RNA expression in hippocampal microglia to reflect that focus and the manuscript's scope. While we do not expand the scope into the role of TIMP2 in microglia in other regions here, we have now included TIMP2 expression in cortex and striatum (**Supplementary Fig. 1d-g**) as requested, from the ABC atlas, while also performing immunohistochemistry and confocal imaging with quantification of the number of TIMP2+ microglia in these areas for enhanced reader interest (**Supplementary Fig. 1p-r**).

2. The quantification of microglial morphology appears somewhat arbitrary and the images used are of low-resolution (Supplemental Fig. 2f, g). The authors should include direct measurements of soma size, branch number, etc., from tissue sections.

Response: Thank you for raising this point. We agree that the morphology analysis we initially included broadly categorizes microglia state with a somewhat rudimentary approach based on circularity. In line with the reviewer's suggestion, we performed a more detailed morphology analysis using high-resolution images that replaces that used in the initial Supplementary Fig. 2f-g. Using Imaris-based 3D reconstruction of microglia from confocal images, we found that TIMP2 KO microglia exhibit significantly larger volume and surface area, a phenotype linked to transition from ramified to reactive/primed and hypertrophic microglia in other conditions[26-29]. We also observed increased oblate ellipticity (**Supplementary Fig. 2i**), a flattened phenotype associated with ameboid microglia that may be associated with a pro-inflammatory state[26,27,30]. TIMP2 KO microglia also exhibit a converse decrease (though not statistically significant, P=0.06) in prolate ellipticity (**Supplementary Fig. 2i**) that is associated with more elongated, responsive microglia[31,32]. We also analyzed branch Sholl intersections (**Supplementary Fig. 2j**), but this effect was not statistically significant. Together, these results indicate that TIMP2 broadly affects microglia morphology, and we have tempered conclusions that link morphology to microglial function (lines 175-181), as there is acknowledged difficulty with strictly interpreting function from morphology within the field[24,25]. We go on to characterize a variety of other

changes in microglia related to their function throughout the manuscript, which we feel substantiates the claims overall.

3. Expression of TIMP2 was only validated in cultured microglia, which do not fully represent endogenous microglia. Validation by IHC or RNA in situ on brain sections is necessary. Additionally, the nature of the KO allele should be clarified—does it result in a null mutation? Why are some transcripts still detectable in the global KO mice (Figure 1d)?

Response: We chose an isolation and culturing protocol that minimizes artificial changes in microglia, but we agree that showing additional validation in brain sections would be useful to fully represent endogenous microglia. In line with the reviewer's suggestion, we have now performed immunohistochemistry and confocal imaging to show microglial expression of TIMP2 in mouse hippocampus, expression that is not observed in the KO hippocampus (**Fig. 1k-l**). We also find approximately 20% of microglia in hippocampus are TIMP2+ (**Fig. 1l-m**), which is roughly similar to the proportion we identified in our *in vitro* work (**Supplementary Fig. 1n**).

The global KO line used for Figure 1d RNA levels (now **Supplementary Fig. 1i**) is a well-established conventional KO model created and described by the Soloway group[34] in which exon 1 is targeted using a conventional knockout strategy, which leads to nonsense-mediated decay of subsequent exons that are not translated. Evaluating the RNAseq data further, we did not detect exon 1 transcripts, as expected, and the very small number of residual transcripts in the remaining exons do not yield detected protein, as reflected in our previous data showing lack of any protein in KO in neurons and from brain lysate using imaging and immunoblotting with independent antibodies[9] and lack of signal in KO brain sections in microglia specifically (**Fig. 1k-l**).

4. The overlap between TIMP2 and CD68 signals was only shown in cultured microglia (Figure 1f, g). This should be replicated using IHC or RNA in situ in vivo. The percentage of TIMP2+ and TIMP2+/CD68+ microglia in vivo, and any regional differences, should be quantified and reported.

Response: Thank you for this suggestion. We agree and have extended the overlap analysis of TIMP2 and CD68 in hippocampal microglia to brain sections as suggested, revealing a similar proportion as in our previously included *in vitro* results, i.e., approximately 80% of TIMP2-positive microglia are CD68-positive (**Fig. 1n-o**). We found that approximately 20% of microglia in the hippocampus are TIMP2+ (**Fig. 1m**). While we focused our in this manuscript mostly on the hippocampus, we took the reviewer's suggestion to examine TIMP2 staining in microglia in other regions, focusing on cortex and striatum. This analysis revealed TIMP2-positive microglia in these brain regions. Interestingly, the proportion of TIMP2+ microglia that co-expressed CD68 is ~80% in both hippocampus and striatum, whereas the proportion is significantly smaller in cortex (**Supplementary Fig. 1o-p**), possibly a reflection of known heterogeneity of microglia throughout the brain[35]. However, in the 3 regions analyzed, ~20% of microglia were TIMP2+ (**Supplementary Fig. 1q**). These results have been added to the manuscript and are now described in Lines 147-155.

5. What is the magnitude of CD68 upregulation in CX3CR1-CreER conditional KO mice compared to the global KO? Does microglia-specific deletion of TIMP2 produce a similar or milder phenotype? This should be quantified using an adequate number of animals and tissue sections.

Response: Due to the broad expression of TIMP2 throughout the brain and high levels in the periphery[36], we would expect that global deletion of TIMP2 may lead to larger effect sizes compared to cell type-specific deletion in neurons or microglia. Additionally, neurons in hippocampus generally have higher levels of TIMP2 compared to microglia[37]. When we compare effect sizes for the number of CD68+ microglia number resulting from deletion across differing deletion approaches from brain sections stained with the same antibodies (using a sufficiently powered number of mice from the same number

of brain sections and the same quantification method), we find that global deletion generally leads to a stronger effect, ~1.64-fold increase vs. WT, compared to ~1.29-fold for neuron-specific KO vs. its corresponding control, and 1.1-fold increase for microglial-specific vs. its corresponding control. To determine standardized magnitudes of effect sizes for these comparisons, we calculated Cohen's d values. Global KO deletion produced the largest standardized effect (d = 1.72), with neuron-specific deletion yielding an intermediate effect (d=1.10), and microglia-specific deletion yielding the smallest effect, though still moderate (d = 0.73). The consistent ordering of effect sizes supports a greater overall impact of global deletion. This finding intuitively matches the level of TIMP2 loss across these deletion scenarios. We have briefly expanded discussion to note this good point raised by the reviewer in lines 425-428.

6. Since senescent microglia are difficult to define due to the lack of specific markers, p16 staining alone is insufficient. Additional orthogonal validation is required.

Response: We agree that additional senescent markers would be helpful for validation here. We have repeated this experiment in *Cx3cr1*$^{CreERT2/+}$;*Timp2*$^{fl/fl}$ and *Timp2*$^{fl/fl}$ control microglia, this time staining for additional markers of senescence markers P21 and SA-beta-gal. Similar to our prior result from p16 staining (Fig. 3d-e), we now report increased number of microglia positive for p21(**Supplementary Fig. 3f-g**), while also increasing staining for SA-beta-Gal (**Supplementary Fig. 3h-i**) in *Cx3cr1*$^{CreERT2/+}$;*Timp2*$^{fl/fl}$ microglia compared to controls (lines 250-252).

7. To illustrate microglial state regulation by TIMP2, the current evidence (bulk RNA-seq from cultured microglia, IHC for CD68 and p16, and snRNA-seq after TIMP2 injection) is not sufficient. Cultured microglia may not reflect in vivo states; CD68 and p16 are nonspecific; and TIMP2 injection is a gain-of-function approach. For the bulk RNA-seq, only GO terms were discussed, with no specific gene-level analysis. The authors should perform transcriptomic profiling on microglia isolated from Timp2 KO (preferably conditional KO) mice and compare this with the bulk RNA-seq and snRNA-seq datasets.

Response: We appreciate the reviewer's comment and have now performed a more in-depth analysis of the bulk RNA-seq dataset that includes ranked GSEA (**Fig. 1d**), a volcano plot showing top differentially expressed genes with over-representation GO analysis (Padj <0.05; **Supplementary Fig. 1h-j**) with gene-level description of specific top genes in the text (lines 129-131) associated with TIMP2 deletion that are involved in myeloid signaling, inflammation, and lysosomal function described in the text. These data support upregulation in sets of genes associated with cell activation involved in the immune response, regulation of cytokine production, and other immune system processes. To balance very pressing cost and time constraints associated with generating an additional snRNAseq dataset to highlight deletion-associated changes that may be converse to those seen with treatment, we performed additional analyses and comparisons across the datasets that may provide further insight into changes with TIMP2 modulation (deletion vs. treatment). We performed a signed module-trait analysis by WGCNA on our KO vs. WT microglia bulk RNAseq microglia dataset to identify significant modules of co-regulated genes (**Fig. 1e**), including a downregulated cyan module and upregulated red module in TIMP2 KO vs WT microglia. Over-representation analyses on genes extracted from the upregulated red module genes reflected enrichment in pathways that included "cell activation", "immune system processes", and pathways linked to phagocytosis, e.g., "response to lipids" in KO microglia (**Fig. 1g**). To provide a comparison with our snRNAseq TIMP2 treatment experiment, we performed a signed hdWGCNA on the microglia cluster from this dataset (**Supplementary Fig. 5q**). This analysis identified a significantly downregulated "brown" module in microglia from TIMP2-treated mice (**Supplementary Fig. 5r**), and over-representation analysis on the extracted genes from this downregulated module revealed significant enrichment in GO biological processes that included "cell activation" and other pathways involved in immune cell (lymphocyte) activation response (**Supplementary Fig. 5r**), which were notably similar terms to those in the KO RNAseq WGCNA red module that was upregulated (opposite direction). Additionally, the downregulated cyan module in the bulk RNAseq dataset (**Fig. 1f**) contains sets of genes associated with cell projection morphogenesis and process

structure/organization, whereas the significantly upregulated blue module from TIMP2 treatment in microglia also contains these related terms but in the opposing direction (**Supplementary Fig. 5s)**. Overall, our approach identifies commonality in general pathways in microglia across conditions in which TIMP2 is deleted or applied as treatment in aged mice, substantiating other data in the manuscript showing that TIMP2 affects microglial state and function across healthy and aged contexts, as reflected in other assays across the manuscript. We describe this further in the discussion, while acknowledging limitations of the approach that point to future directions related to TIMP2 regulation in microglia (lines 473-477).

8. Marker genes from key microglial clusters identified in the snRNA-seq analysis should be presented. Currently, only GO terms are shown. Changes in some of the key clusters should also be validated via IHC or flow cytometry.

Response: We have now included violin plots of specific marker genes from all microglial subclusters in **Supplementary figure 5k**. To further validate the observation of a reduced proportion of microglia in the key subcluster 6 in TIMP2-treated hippocampi, we performed IHC and confocal imaging in vehicle- and TIMP2-treated aged mice and performed quantification of the number of microglia expressing TLR7, a top marker gene for the cluster. Corroborating this top subcluster's reduced proportion, we found that the number of TLR7-positive microglia is significantly reduced in TIMP2-treated hippocampi compared to vehicle-treated mice (**Supplementary Fig. 5m-n**). Additionally, our snRNAseq results had shown one of the top significantly increased genes altered by TIMP2 treatment in microglia is *Tmsb4x* (**Fig. 5m**), a gene linked to negative regulation of NFkB signaling[38]. We performed IHC and confocal imaging for this marker as well in TIMP2-treated and vehicle-treated mice, revealing a significantly increase in TMSB4X+ microglia (**Fig. 5n-p**), as predicted from our snRNAseq analysis. These additional data validate top genes and markers altered in microglia with TIMP2 treatment, including subcluster 6, a subcluster we used to follow up on in additional imaging studies that were included.

9. Engulfment of synaptic particles was assessed only in TIMP2-injected mice. The same analysis should be performed in TIMP2 KO mice to complete the comparison.

Response: Thank you for this excellent suggestion. To address this point, we have now performed an additional experiment to examine microglial synapse engulfment in brain sections from 6-7-month-old WT and TIMP2 KO mice using the approach employed in our TIMP2 treatment experiment in aged mice described in Figure 5. We now report that microglia from TIMP2 KO mice exhibit significantly less volume of $VGLUT1^+$ puncta within microglia lysosomes compared to WT controls (**Fig. 2g-h;** line 185-189), further substantiating the overall effect of TIMP2 on phagocytosis in a bidirectional fashion when considering the effect of TIMP2 treatment in Fig. 5, while also expanding functional phenotypes observed with TIMP2 deletion in the study. This largely fits with other new data we have now added to complete another comparison, which shows opposing effects on myelin phagocytosis in primary microglia in the context of TIMP2 deletion or TIMP2 treatment (new **Supp. Fig. 5t-v;** lines 419-421) to complement data in Fig. 4 in the initial manuscript.

Minor:
1. Line 118–119: If P5–P8 mice were used, they should be described as early postnatal (as opposed to neonatal), and the exact age range should be noted.

Response: Thank you for this correction. We have now corrected this so that when referring to P5-P8 mice, we describe them as “early postnatal” throughout the manuscript. The age range for what was originally line 118-119 referenced is now indicated in the text of the manuscript in addition to the figure legends.

2. Line 247–248: Clarify what is meant by "time-dependent fashion." Does TIMP2 accumulate cumulatively in culture, or does secretion increase over time?

Response: Thank you for this great point. The use of the phrase “time-dependent fashion” may indeed be misunderstood by readers. Our study design does not allow us to distinguish between the two possibilities mentioned, so we have adjusted the language to more conservatively describe the result here of secretion in culture to avoid confusion, as seen in lines 270-271.

3. Line 228: The correct reference should be to Supplemental Figure 1e.

Response: Thank you for bringing this error to our attention, which we have now corrected.

4. Figure 5i, j, k: Cluster identities should be labeled directly on the figures to aid interpretation.

Response: We agree that this will aid interpretation and have now notated the cluster identities above the respective bar graph for ease of reference and projected on top of the subclusters with white outline (**Fig. 5g and Supplementary Fig. 5h**).

Reviewer #4 (Remarks to the Author):

Summary: The goal of this manuscript is to explore how the age-rejuvenating factor, TIMP2, exerts its protective effects on the brain. This paper is a follow up to the group's previous work to show improvements in synaptic function and learning and memory with TIMP2, but the first of its kind to show that TIMP2 plays an unappreciated role in microglial function and neuroinflammation. Using complimentary approaches including TIMP2 deletion (global or cell type specific) as well as TIMP2 treatment showed that bidirectionally modulating TIMP2 levels has profound effects on microglia, which is novel function for this protein. This study is rigorously done and insights from this work are important for aging and age-related neurodegenerative diseases. This study is particularly noteworthy given the translational value of these studies. For example, the TIMP2 treatment study in 20 mo. old mice that clearly shows TIMP2's protective effect that is lost with aging.

While this reviewer maintains strong enthusiasm for this work, there are some minor points that need clarification:

Response: We would like to thank the reviewer for noting that the study is "rigorously done, important for aging and age-related neurodegenerative disease, and noteworthy given the translational value". We have addressed the reviewer's constructive points that we feel have improved the manuscript, as outlined below.

1. Line 47, 95, 136: There has been a recent push for consistency of microglia nomenclature (PMID: 36327895), which adds some confusion to what different terms may mean. Terms like "microgliosis" or "activation" are sometimes hard to interpret, or perceived to be outdated. In the introduction, "microgliosis" or "activation" is used when describing changes in microglia due to aging. Can the authors clarify or define these terms for the reader to avoid confusion? Does aging have its own microglial phenotype that is different than disease associated phenotypes typically described in fields like Alzheimer's? Given the lack of consensus in the field, it might be better to say "microglia" instead of "microglial state", since it's unclear what "microglial state" means in this paper and whether TIMP2 expression in microglia modulates its own "microglial state" since now all microglia are TIMP2+.

Response: Thank you for this point. The reviewer is correct that these terms are difficult to interpret, and indeed there is not broad consensus for their definitions. We agree that it's important to avoid confusion and enhance characterization in the terminology. We have eliminated use of "microgliosis" given its myriad usages and have attempted to use more descriptions of specific phenotypes throughout the text rather than relying on overly simplified terminology where appropriate. While microglial activation can simplistically refer to any deviation from homeostatic microglial state characterized by coordinated changes in gene expression, morphology, and function, these definitions can be challenging and depend on context, so we have limited reliance on a single term where appropriate in favor of defining the phenotypes. Microglia encounter similar challenges (myelin debris, etc) throughout aging and AD progression, perhaps leading to similar alterations in transcriptomic states[39] and other changes, while there are likely distinct phenotypes adopted in these different pathological states. This is an area of active study that we expect will provide more clarity for the field in future work. We have adjusted unclear terms throughout the manuscript where appropriate.

2. Not all TIMP2+ microglia are CD68+ and not all CD68+ microglia are TIMP2+. Do the authors know why this is? What other markers does the TIMP2+ microglia express (e.g. homeostatic v disease reactive) that may help provide understand why TIMP2 is important in the setting of aging? Can the authors speculate?

Response: Thank you for this interesting question. We have now expanded our characterization of TIMP2+ cells co-expressing CD68 by immunohistochemistry and confocal imaging in mouse brain sections (**Fig. 1k-o**), revealing that the vast majority of TIMP2+ microglia are also CD68+ (~80%). The

remaining smaller portion may reflect a pool of TIMP2+ microglia involved in other processes beyond phagolysosomal activity; we speculate they may be involved in other functions related to TIMP2, perhaps extracellular matrix remodeling or regulation of neuron-glia signaling, or that TIMP2+ microglia not expressing CD68 may reflect intermediate (less phagolysosomal) states, particularly in aging where microglia may adopt partial or less robust programs than those that respond to AD pathology or other challenges, states that are themselves highly heterogeneous. We have briefly included speculations of TIMP2's other possible roles in microglia in our discussion (lines 455-461, 473-477), which we feel provides an improved framework for understanding the findings.

3. Fig 2: this is the global knockout, correct? Please explicitly state that.

Response: Yes, this is the global knockout. We have explicitly stated this in line 539 to clarify this.

4. Fig 2b-c: what does each circle represent on the bar graphs? It should represent each mouse but the figure legend says sample size n=9-13 but there are more circles than that. Can you clarify?

Response: Thank you for the good catch, as this should have indicated 9-13 per group per sex for a total of 19-24. Each circle does represent an individual mouse, so we have corrected the description in the legend to reflect that both sexes are included in each group (since there were no sex differences). We have thoroughly reviewed all figure legends to confirm they reflect the correct sample sizes.

5. Line 156-157: Since there is no increase in total number of microglia, does this suggest in increase in cell size? Was this quantified?

Response: Yes, the percentage area occupied by IBA1 staining is often used as a proxy for changes in cell size and/or morphology, especially since we detected no significant changes in total number of IBA1+ cells. Applying a detailed Imaris-based morphology analysis, we have now found that TIMP2 KO microglia exhibit a larger volume and surface area compared to WT microglia (**Supplementary Fig. 2f-h**), supporting the concept the reviewer raises. (We focused on quantifying the 6-7-month-old cohort as opposed to the 3-4-month-old WT and KO mice because the IBA1 coverage in this cohort was not significantly different in the younger age, as detailed in the initial submission). These data are now described in line 176-181.

6. Line 179: The statement "This technique has not yet been extensively applied to measure brain immune proteins" should be removed or tempered. Other groups, PMID: 27481936 for example, have used in vivo microdialysis to investigate the ISF proteome and showed enrichment for inflammatory related proteins relative to Alzhiemer's disease and sleep deprivation.

Response: We apologize for the oversight and our imprecise language used and agree we did not adequately reflect prior work demonstrating successful use of in vivo microdialysis to characterize brain interstitial fluid inflammatory proteins, including in AD pathology and sleep deprivation (PMID: 27481936). We have revised the text, along with including this citation in this sentence, to correct our statement to reflect that while in vivo microdialysis has been effectively applied in these contexts in PMID 27481936, the broader use of the technique for profiling of brain immune proteins remains less common relative to other prior uses of microdialysis (see line 198-200).

7. Figure 3: The white arrows in panel b and d are very distracting. I'd recommend removing.

Response: We agree that this can be distracting, but for consistency across figures, we would prefer to retain arrowheads to highlight what is being quantified where this is difficult to tell. To improve the issue, we have altered their size, which appears less distracting (**Fig.3 b,d**).

8. Line 237: Consider change “external pools” to “ISF” or “extracellular” pools.

Response: Thank you for suggesting this. Extracellular pools is indeed more accurate and specific; we have changed this line accordingly (first instance, line 260 and then throughout).

9. Line 249: Clarify what zymosan does and why it was chosen? Are both zymosan and LPS used as an inflammatory challenge? Could the lack of effects be due to dose chosen?

Response: Labeled Zymosan was chosen among several standard stimuli with which to examine phagocytosis, as it’s a β-glucan–rich cell wall preparation derived from *Saccharomyces cerevisiae* widely used as a common innate immune stimulus. The zymosan dose was chosen based on previous studies utilizing this dose and its known impact on glia that has been previously published[40] and through our own independent optimization. Myelin, zymosan, and LPS all initiate a stimulatory response in microglia, but myelin is an especially relevant substrate for the brain. We have added a citation to the dose of zymosan employed within the text when introduced (line 272).

10. Figure 5: Did TIMP2 treatment in vitro increase total # of Iba1+ microglia?

Response: We did not observe any changes in microglia number following TIMP2 treatment *in vivo*. To examine this *in vitro*, we quantified the number of cells *in vitro* and found no significant change in total cell number resulting from TIMP2 treatment (**Response Fig. 2**).

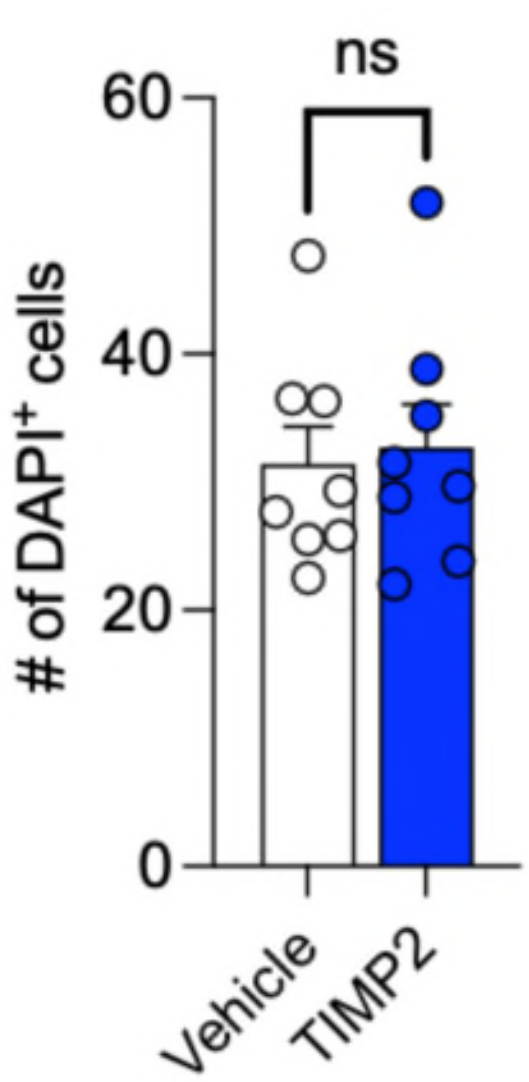

Response Fig. 2: Quantification of the number of total cells in the primary culture (determined to be ~100% microglia in purity analyses) for each treatment group, revealing that TIMP2 treatment did not affect total microglia number.

References Cited

1 Li, S., Wernersbach, I., Harms, G. S. & Schäfer, M. K. Microglia subtypes show substrate-and time-dependent phagocytosis preferences and phenotype plasticity. *Frontiers in Immunology* **13**, 945485 (2022).

2 Dolan, M.-J. *et al.* Exposure of iPSC-derived human microglia to brain substrates enables the generation and manipulation of diverse transcriptional states in vitro. *Nature immunology* **24**, 1382-1390 (2023).

3 Song, L., Lee, C. & Schindler, C. Deletion of the murine scavenger receptor CD68. *J Lipid Res* **52**, 1542-1550 (2011).

4 Wu, Y. *et al.* Microglial lysosome dysfunction contributes to white matter pathology and TDP-43 proteinopathy in GRN-associated FTD. *Cell Rep* **36**, 109581 (2021).

5 Marschallinger, J. *et al.* Lipid-droplet-accumulating microglia represent a dysfunctional and proinflammatory state in the aging brain. *Nat Neurosci* **23**, 194-208 (2020).

6 Tanaka, Y., Chambers, J. K., Matsuwaki, T., Yamanouchi, K. & Nishihara, M. Possible involvement of lysosomal dysfunction in pathological changes of the brain in aged progranulin-deficient mice. *Acta neuropathologica communications* **2**, 1-15 (2014).

7 Mazher, M., Moqidem, Y. A., Zidan, M., Sayed, A. A. & Abdellatif, A. Autophagic reprogramming of bone marrow-derived macrophages. *Immunol Res* **71**, 229-246 (2023).

8 Kida, E. *et al.* Distribution of Tripeptidyl Peptidase I in Human Tissues Under Normal and Pathological Conditions. *Journal of Neuropathology & Experimental Neurology* **60**, 280-292 (2001).

9 Ferreira, A. C. *et al.* Neuronal TIMP2 regulates hippocampus-dependent plasticity and extracellular matrix complexity. *Mol Psychiatry* **28**, 3943-3954 (2023).

10 Nguyen, P. T. *et al.* Microglial Remodeling of the Extracellular Matrix Promotes Synapse Plasticity. *Cell* **182**, 388-403 e315 (2020).

11 Zhu, B. *et al.* G-protein-coupled receptor ADGRG1 drives a protective microglial state in Alzheimer's disease through MYC activation. *Neuron* **113**, 3224-3242 e3227 (2025).

12 Park, D. *et al.* BAI1 is an engulfment receptor for apoptotic cells upstream of the ELMO/Dock180/Rac module. *Nature* **450**, 430-434 (2007).

13 Barger, S. W. & Basile, A. S. Activation of microglia by secreted amyloid precursor protein evokes release of glutamate by cystine exchange and attenuates synaptic function. *Journal of neurochemistry* **76**, 846-854 (2001).

14 Maezawa, I. & Jin, L. W. Rett syndrome microglia damage dendrites and synapses by the elevated release of glutamate. *J Neurosci* **30**, 5346-5356 (2010).

15 Bohlen, C. J., Bennett, F. C. & Bennett, M. L. Isolation and Culture of Microglia. *Curr Protoc Immunol* **125**, e70 (2019).

16 Czirr, E. *et al.* Microglial complement receptor 3 regulates brain Abeta levels through secreted proteolytic activity. *J Exp Med* **214**, 1081-1092 (2017).

17 Vijaya, A. K., Iešmantaitė, M., Mela, V., Baltriukienė, D. & Burokas, A. Microglia isolation from aging mice for cell culture: A beginner’s guide. *Frontiers in Cellular Neuroscience* **17**, 1082180 (2023).

18 Zhuang, H. *et al.* Accelerated age-related decline in hippocampal neurogenesis in mice with noise-induced hearing loss is associated with hippocampal microglial degeneration. *Aging* **12**, 19493-19519 (2020).

19 Stillman, J. M. *et al.* Lipofuscin-like autofluorescence within microglia and its impact on studying microglial engulfment. *Nat Commun* **14**, 7060 (2023).

20 Bodea, L. G. *et al.* Neurodegeneration by activation of the microglial complement-phagosome pathway. *J Neurosci* **34**, 8546-8556 (2014).

21 Safaiyan, S. *et al.* Age-related myelin degradation burdens the clearance function of microglia during aging. *Nat Neurosci* **19**, 995-998 (2016).

22 Halder, S. K. & Milner, R. Exaggerated hypoxic vascular breakdown in aged brain due to reduced microglial vasculo-protection. *Aging cell* **21**, e13720 (2022).

23 Shea, J. M. & Villeda, S. A. Microglia aging in the hippocampus advances through intermediate states that drive activation and cognitive decline. *Elife* **13** (2025).
24 Vidal-Itriago, A. *et al.* Microglia morphophysiological diversity and its implications for the CNS. *Frontiers in immunology* **13**, 997786 (2022).
25 Green, T. R. & Rowe, R. K. Quantifying microglial morphology: an insight into function. *Clinical and experimental immunology* **216**, 221-229 (2024).
26 Torres-Platas, S. G. *et al.* Morphometric characterization of microglial phenotypes in human cerebral cortex. *J Neuroinflammation* **11**, 12 (2014).
27 Ayoub, A. E. & Salm, A. K. Increased morphological diversity of microglia in the activated hypothalamic supraoptic nucleus. *J Neurosci* **23**, 7759-7766 (2003).
28 Reddaway, J., Richardson, P. E., Bevan, R. J., Stoneman, J. & Palombo, M. Microglial morphometric analysis: so many options, so little consistency. *Front Neuroinform* **17**, 1211188 (2023).
29 Nimmerjahn, A., Kirchhoff, F. & Helmchen, F. Resting microglial cells are highly dynamic surveillants of brain parenchyma in vivo. *Science* **308**, 1314-1318 (2005).
30 Szabo-Pardi, T. A., Syed, U. M., Castillo, Z. W. & Burton, M. D. Use of Integrated Optical Clearing and 2-Photon Imaging to Investigate Sex Differences in Neuroimmune Interactions After Peripheral Nerve Injury. *Front Cell Dev Biol* **9**, 624201 (2021).
31 Ziebell, J. M., Taylor, S. E., Cao, T., Harrison, J. L. & Lifshitz, J. Rod microglia: elongation, alignment, and coupling to form trains across the somatosensory cortex after experimental diffuse brain injury. *J Neuroinflammation* **9**, 247 (2012).
32 Xie, M. *et al.* Rod-shaped microglia interact with neuronal dendrites to attenuate cortical excitability during TDP-43-related neurodegeneration. *Immunity* **58**, 3113-3129 e3118 (2025).
33 Castellano, J. M. *et al.* Human umbilical cord plasma proteins revitalize hippocampal function in aged mice. *Nature* **544**, 488-492 (2017).
34 Wang, Z., Juttermann, R. & Soloway, P. D. TIMP-2 is required for efficient activation of proMMP-2 in vivo. *The Journal of biological chemistry* **275**, 26411-26415 (2000).
35 Ayata, P. *et al.* Epigenetic regulation of brain region-specific microglia clearance activity. *Nature neuroscience* **21**, 1049-1060 (2018).
36 Peeney, D. *et al.* Whole organism profiling of the Timp gene family. *Matrix Biology Plus* **18**, 100132 (2023).
37 Zhang, Y. *et al.* An RNA-sequencing transcriptome and splicing database of glia, neurons, and vascular cells of the cerebral cortex. *J Neurosci* **34**, 11929-11947 (2014).
38 Wang, M. *et al.* Thymosin beta4 reverses phenotypic polarization of glial cells and cognitive impairment via negative regulation of NF-kappaB signaling axis in APP/PS1 mice. *J Neuroinflammation* **18**, 146 (2021).
39 Keren-Shaul, H. *et al.* A Unique Microglia Type Associated with Restricting Development of Alzheimer's Disease. *Cell* **169**, 1276-1290 e1217 (2017).
40 Machlovi, S. I. *et al.* APOE4 confers transcriptomic and functional alterations to primary mouse microglia. *Neurobiol Dis* **164**, 105615 (2022).
